# Utilizing Proteolytic‐Resistant Nano‐Short Peptide Based on Naphthyl Tail‐Anchored to Combat Bacterial Infections

**DOI:** 10.1002/advs.202508854

**Published:** 2025-09-15

**Authors:** Xi Yan, Yinfeng Lyu, Yi Liu, Jianping Ren, Yue Zhao, Licong Zhang, Anshan Shan

**Affiliations:** ^1^ College of Animal Science and Technology Northeast Agricultural University Harbin 150030 The People's Republic of China

**Keywords:** anti‐enzymolysis motif, nano‐short peptide, naphthyl tail‐anchored, proteases

## Abstract

The extensive application of antimicrobial peptides (AMPs) as viable alternatives to antibiotics is constrained by their high susceptibility to enzymatic degradation by proteases. In this study, a series of potent nano‐short peptides are engineered based on the short peptide anti‐enzymolysis motif “R^D^RRP” by introducing different hydrophobic groups using different strategies. The validity of the self‐assembly system triggered by naphthyl tail anchoring is confirmed through a comprehensive analysis of the bioactivity and nanoproperties of these nano‐short peptides. The naphthyl tail‐anchored N_4_ peptide (Nal‐Nal‐Nal‐Nal‐R^D^‐R‐R‐P) could self‐assemble into nanofibers in aqueous solutions, exhibiting potent and broad‐spectrum antimicrobial activity with excellent biocompatibility (Geometric Mean of the Minimum Inhibitory Concentration (GM_MIC_) = 5.04, Geometric Mean of the Selectivity Index (GM_SI_) = 50.8) and remarkable biostability against physiological challenges (salt concentrations, serum components, and various proteases). More importantly, the low resistance propensity for N_4_ is attributed to multiple antimicrobial mechanisms combining physical membrane‐breaking and energy metabolism disruption. Its efficacy is substantiated in both *Escherichia coli* (*E. coli*) induced murine peritonitis‐sepsis and Methicillin‐Resistant *Staphylococcus aureus* (*MRSA)* mediated skin infection models in mice. In summary, these findings advance the design of AMPs with enhanced protease resistance and the development of peptide‐based nanomaterials for biomedical applications.

## Graphical abstract

Antimicrobial peptides (AMPs) show potential as alternatives to antibiotics for bacterial infections; however, their susceptibility to proteases limits wider application. In this study, N_4_ nanofibers are constructed by integrating anti‐enzymolysis motif into the self‐assembly system triggered by naphthyl‐anchored. N_4_ demonstrates excellent proteolytic resistance and proves effective against bacterial infections in peritonitis sepsis and skin wound models.

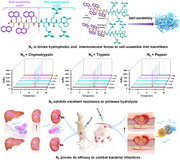

## 1 Introduction

The discovery and widespread adoption of antibiotics represent one of the most significant advancements of the 20^th^ century,^[^ ^1^, ^2^ ^]^ having profoundly advanced the medical field and raised global healthcare standards. However, the misuse of these drugs has led to the emergence and dissemination of multidrug‐resistant bacteria (MDR). This phenomenon has increased healthcare costs and patient mortality,^[^ ^3^, ^4^, ^5^ ^]^ presenting a significant challenge to global public health security. Consequently, the development of antimicrobial agents with low resistance tendency represents a promising strategy to address the issue of drug‐resistant bacteria.^[^ ^6^ ^]^

Antimicrobial peptides (AMPs), as a crucial component of the host immune system, have attracted significant interest due to their broad‐spectrum and efficient antimicrobial activity, as well as their low propensity to induce resistance.^[^ ^7^ ^]^ Although the antimicrobial mechanism of AMPs is not yet fully understood, it has been widely proposed that they act by disrupting bacterial cell membranes, inhibiting cell wall synthesis, and interfering with nucleic acid and protein synthesis.^[^ ^8^ ^]^ This multifaceted mechanism may contribute to the reduced likelihood of bacteria developing resistance to AMPs.

Although AMPs have shown promising potential as antimicrobial agents with low tendency for resistance,^[^ ^6^ ^]^ their broader application is significantly hindered by their high susceptibility to protease hydrolysis. To overcome this limitation, numerous strategies have been proposed for the innovative design of AMPs, including approaches such as the incorporation of protease inhibitors,^[^ ^9^, ^10^ ^]^ structural cyclization,^[^ ^11^, ^12^ ^]^ specialized amino acid arrangements,^[^ ^13^, ^14^ ^]^ the development of nanostructures,^[^ ^15^, ^16^ ^]^ and the substitution of non‐natural amino acids.^[^ ^17^, ^18^, ^19^, ^20^ ^]^ Notably, some of these strategies, especially those that involve the introduction of redundant structures, can increase the complexity of structure while enhancing the stability of AMPs, potentially increasing their toxicity and decreasing their activity. Cationic short peptides exhibit significant application potential, benefit from their simple structures, and have been extensively utilized as small molecular units in the novel design of AMPs.^[^ ^21^, ^22^ ^]^ Nevertheless, strategies to enhance the protease stability of cationic short peptides remain relatively unexplored. Owing to their limited number of amino acids, short peptides typically contain fewer protease cleavage sites, potentially conferring inherent resistance to protease hydrolysis. Therefore, a promising strategy for developing AMPs with enhanced resistance to protease hydrolysis involves creating a short peptide anti‐enzymolysis motif through specific modifications based on the properties of cationic amino acids. While short peptides may exhibit enhanced stability, they may not possess antimicrobial activity due to the limited number of amino acids. In recent years, nano‐delivery systems have experienced extensive development and application, primarily for their efficacy in drug delivery and sustained release, thereby enhancing the bioavailability.^[^ ^23^ ^]^ Consequently, the construction of nanostructures based on bioactive molecules facilitates adaptation to complex physiological environments and accelerates their clinical translation.^[^ ^24^ ^]^ Similarly, the booming development of nanotechnology has facilitated the design of nanostructures for innovative AMPs development.^[^ ^25^, ^26^, ^27^, ^28^ ^]^ Nanoscale engineering of AMPs offers a dual advantage: it increases local effective concentration through enrichment effects, while shielding protease recognition sites via steric hindrance.^[^ ^15^, ^29^ ^]^ This synergistic approach significantly enhances both the biological stability and efficacy of AMPs in complex physiological environments, thereby unlocking their broader application prospects. Hence, we hypothesize that nanomodification of short peptide anti‐enzymolysis motif could further enhance their stability while compensating for their limited antimicrobial activity, thereby facilitating the development of highly efficient and stable peptide‐based antimicrobial materials.

**Figure 1:**
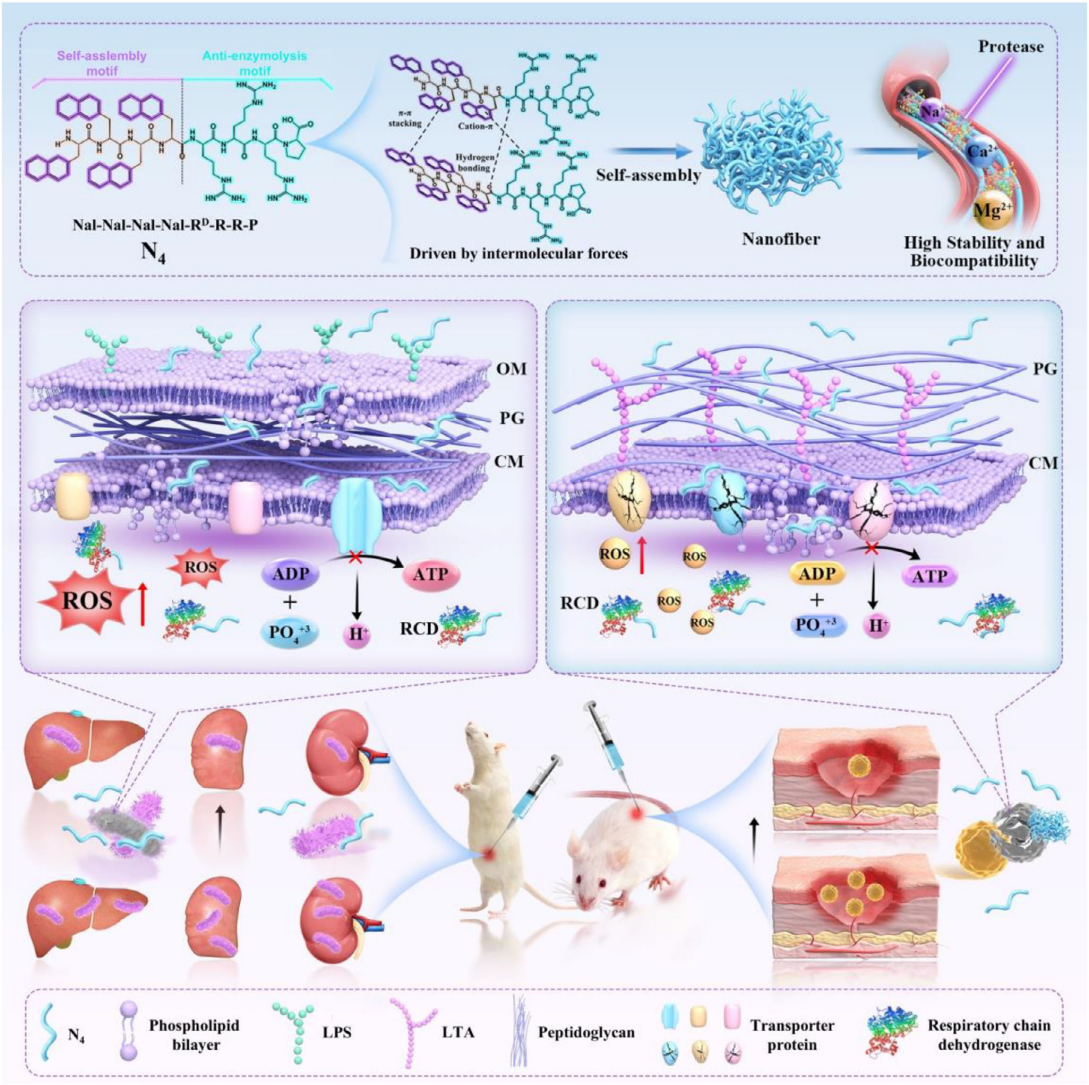
Elucidating the stability and multimodal bactericidal mechanisms (including membrane disruption and energy metabolism interference) of N_4_ nanofibers, and proving efficacy against bacterial infections in both peritonitis‐sepsis and skin wound models.

Building on prior research, this study designed the nano‐short peptides using a specific strategy. First, the short peptide anti‐enzymolysis motif was selected as a positively charged core, which was then coupled with varying numbers of alanine residues to create a template for subsequent modifications. Subsequently, the template was then modified in two ways: either the alanine was completely replaced with different saturated fatty acids or aromatic hydrophobic groups, or an aliphatic butyl or aromatic naphthyl group was anchored to the tail of the alanine sequence. The primary objective of these modifications was to introduce distinct hydrophobic groups to facilitate the nanoscale transformation of the short peptide anti‐enzymolysis motif while imparting necessary hydrophobicity. This study also systematically compared the effects of these structural modifications on the biological activity and nano‐structural properties of the short peptides under the same platform. Our findings indicated that the naphthyl tail‐anchored nano‐short peptide N_4_ could self‐assemble to form nanofiber structures in an aqueous medium. Importantly, N_4_ demonstrated excellent, broad‐spectrum antimicrobial efficacy and biocompatibility (Geometric Mean of the Minimum Inhibitory Concentration (GM_MIC_) = 5.04, Geometric Mean of the Selectivity Index (GM_SI_) = 50.8) while retaining potent antimicrobial activity in the presence of physiological salts, serum, and various proteases. Mechanistically, N_4_ was found to damage the bacterial surface by anchoring to and recognizing anionic components, which facilitated subsequent internal penetration and disruption of energy metabolism, ultimately leading to bacterial cell death. These properties suggest that N_4_ holds significant potential for treating *Escherichia coli* (*E. coli*) induced murine peritonitis‐sepsis and Methicillin‐Resistant *Staphylococcus aureus* (*MRSA)* mediated skin infection models in mice (**Figure** **1**).

## 2 Results and Discussion

### 2.1 Construction and Characterization of Nano‐Short Peptide Libraries Mediated by Hydrophobic Modification

**Figure 2:**
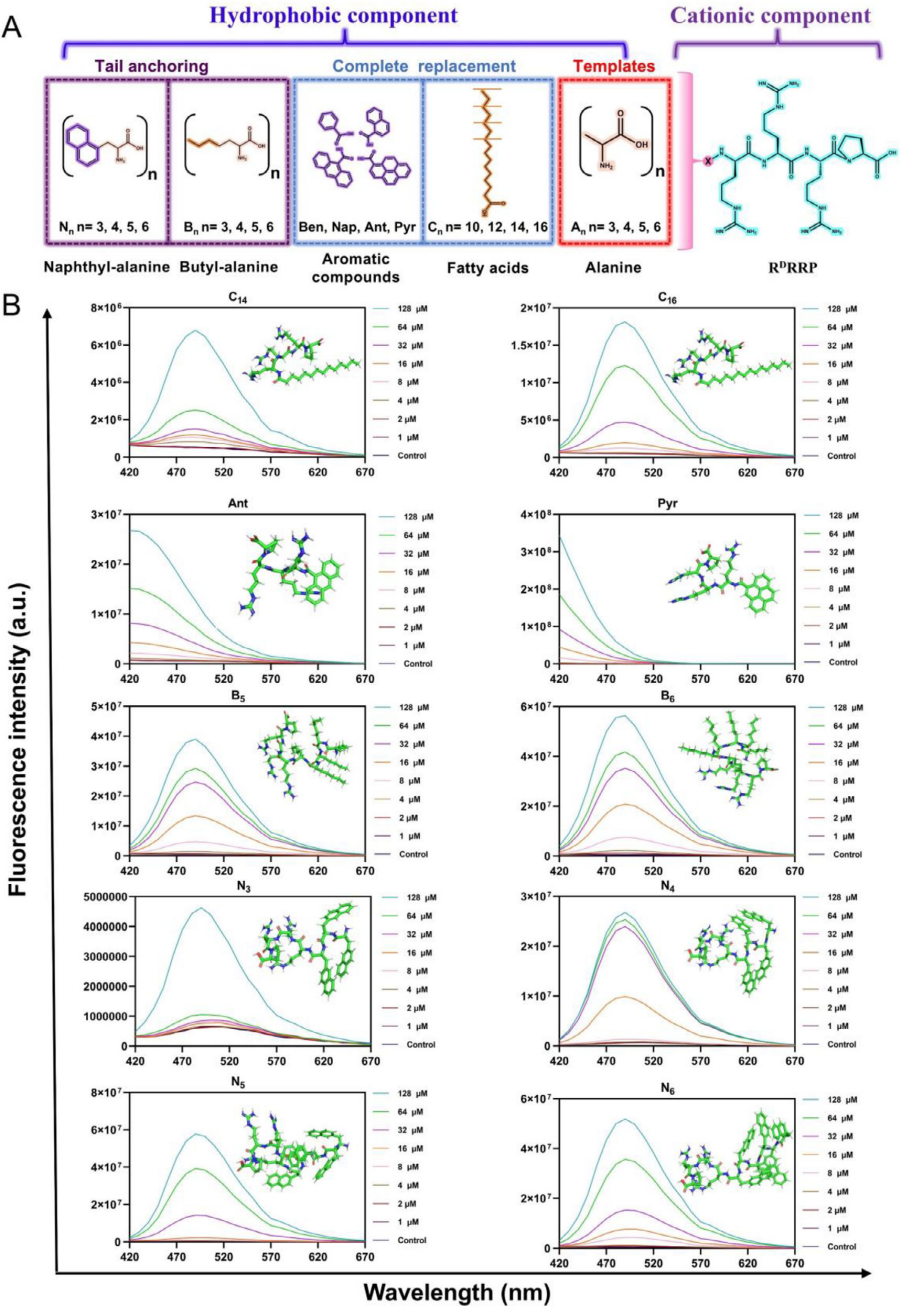
The design and characterization of nano‐short peptides. A) Design principles of nano‐short peptides. B) 1‐aniline‐8‐naphthalenesulfonate (ANS) fluorescence intensity changes and 3D structure diagram of nano‐short peptides.

In our previous study, the cationic short peptide “R^D^RRP” was successfully constructed by integrating the rational arrangement of amino acids, as well as the targeted substitution of D‐type amino acids,^[^ ^30^ ^]^ and showed excellent resistance to proteolytic hydrolysis. The “R^D^RRP” peptide consisted of four amino acids and three positive charges. Driven by the stability and strong cationic character of “R^D^RRP”, this study focused on designing novel nano‐short peptides based on it to explore broader applications. Overall, the cationic nature of AMPs allows them to initially anchor to the negatively charged components of bacterial membranes by electrostatic attraction, while their hydrophobicity confers the ability to bind to the lipophilic structural domains of bacteria, which ultimately induces membrane damage.^[^ ^31^ ^]^ Building on this principle, the cationic short peptide sequence “R^D^RRP” was identified as the positively charged core for designing nano‐short peptides. Unnatural hydrophobic groups were incorporated into the “R^D^RRP” sequence to provide the necessary hydrophobicity and facilitate nanoscale transformation. As shown in **Figure** **2**A, “R^D^RRP” was initially conjugated with varying numbers of alanine residues, creating a series of template peptides denoted as A_n_ (where *n* = 3, 4, 5, 6). Alanine was selected due to its shorter side chain, which provided a simple scaffold for incorporation of hydrophobic groups without introducing excessive structural complexity, thereby mitigating any potential risk of reducing the application potential of the resulting nano‐short peptides. Subsequently, we employed two different strategies for the nanomodification of the A_n_‐series template peptides. Strategy (i) involved the complete substitution of alanine with hydrophobic groups. The groups included saturated fatty acids of varying chain lengths (C_n_: *n* = 10, 12, 14, 16) and aromatic compounds with different numbers of benzene rings, including single (benzoic acid, Ben), double (naphthoic acid, Nap), triple (9‐anthracenecarboxylic acid, Ant), and quadruple (pyrenecarboxylic acid, Pyr) rings. While aliphatic and aromatic hydrophobic moieties are widely utilized in the design of effective AMPs, they can sometimes lead to increased toxicity or decreased activity as the fatty acid chain length or the number of benzene rings increases, which may limit the ability to design optimal nano‐short peptides. To address this, a second modification was conducted in this study: tail anchoring of alanine by hydrophobic groups. This approach involved repeated introduction of highly biocompatible hydrophobic groups into the AMP sequence to enhance the activity while avoiding the significant increase in toxicity caused by breaking the hydrophobic boundary to a certain extent. For this strategy, butyl (with shorter alkyl chains) and naphthyl (with fewer benzene rings) groups were chosen for tail anchoring to all alanine residues. As illustrated in Figure 2A, a series of B_n_ (*n* = 3, 4, 5, 6) nano‐short peptides coupled with different numbers of butyl‐alanine (2‐aminoheptanoic acid, Bua) and a series of N_n_ (*n* = 3, 4, 5, 6) nano‐short peptides coupled with different numbers of naphthyl‐alanine (3‐(1‐naphthyl)‐L‐alanine, Nal) were obtained. Overall, a series of nano‐short peptides were engineered by introducing distinct hydrophobic groups into the “R^D^RRP” anti‐enzymolysis motif using different strategies, and the potential relationship between these modifications and the biological activity and self‐assembly properties of nano‐short peptides was explored.

Subsequently, the purity and molecular weight of the synthesized nano‐short peptides were preliminarily evaluated by reversed‐phase high‐performance liquid chromatography (RP‐HPLC) and mass spectrometry (MS) (Figures S1 and S2, Supporting Information). The sequence information and key physicochemical parameters of all engineered nano‐short peptides are presented in Table S1 (Supporting Information). Hydrophobic modifications with different substituents confer varying molecular weights to the nano‐short peptides: A_n_ series (797.34‐1011.6), fatty acid substitutions (737.86‐822.5), aromatic hydrophobic groups substitutions (688.26‐812.26), B_n_ series (966.42‐1348.74), and N_n_ series (1176.48‐1768.98). The successful synthesis of these peptides was confirmed by their high purity (> 95%) and the strong agreement between their measured and theoretical molecular weights. The critical aggregation concentration (CAC) was employed to assess the aggregation tendency of the nano‐short peptides. Next, changes in fluorescence intensity of these peptides in aqueous solutions were assessed using 1‐aniline‐8‐naphthalenesulfonate (ANS) to provide an initial evaluation of their self‐assembly characteristics. The results (Figure 2B and Figure S3, Supporting Information) revealed a correlation between structural modifications and aggregation propensity, highlighting the critical role of hydrophobic groups in driving the self‐assembly behavior of these nano‐short peptides. For better structural understanding, 3D molecular modeling was employed to visualize the molecular structure of engineered nano‐short peptides (Figure 2B and Figure S3, Supporting Information). The CAC values of the nano‐short peptides was determined by linear fitting of representative fluorescence intensities (Figure S4, Supporting Information). Under the tested conditions, the CAC values for short‐peptides exhibiting nano‐structural propensity were determined as follows: C_14_ (36.91 × 10^−6^ m), C_16_ (17.92 × 10^−6^ m), Ant (11.04 × 10^−6^ m), Pyr (6.91 × 10^−6^ m), B_5_ (3.34 × 10^−6^ m), B_6_ (3.31 × 10^−6^ m), N_3_ (62.79 × 10^−6^ m), N_4_ (5.78 × 10^−6^ m), N_5_ (11.66 × 10^−6^ m), and N_6_ (6.65 × 10^−6^ m). Notably, the alanine‐based template peptides did not demonstrate significant aggregation behavior, as the hydrophobicity of alanine residues alone exhibited limited ability to drive self‐assembly in aqueous media. Following the substitution of alanine with hydrophobic groups, peptides like C_14_, C_16_, Ant, and Pyr exhibited a marked concentration‐dependent increase in ANS fluorescence intensity, indicating a greater degree of aggregation in aqueous media. Similar aggregation behavior was observed for nano‐short peptides B_5_, B_6_, and all of the N_n_‐series with naphthyl tail‐anchored modifications. Furthermore, the fluorescence intensity and CAC values serve as the indicator of the extent of aggregation. The introduction of aromatic hydrophobic groups resulted in the nano‐short peptides exhibiting ANS fluorescence spectra with relatively higher fluorescence intensities and lower CAC values, suggesting that the polycyclic structure of the aromatic groups endows them with a stronger tendency to self‐assemble. Besides hydrophobic interactions, the intermolecular forces from the cyclic structure of the aromatic hydrophobic groups contributed to the enhanced self‐assembly properties of the nano‐short peptides.^[^ ^28^, ^32^ ^]^ Overall, these findings demonstrate that certain hydrophobic modifications, including fatty acid chains longer than C_14_, aromatic systems with more than three benzene rings, sequences containing over five butyl‐alanine, or more than three naphthyl‐alanine, provide sufficient driving force to induce the peptides to form nanostructures.

### 2.2 In Vitro Biological Activity Systematic Evaluation of Nano‐Short Peptides

**Figure 3:**
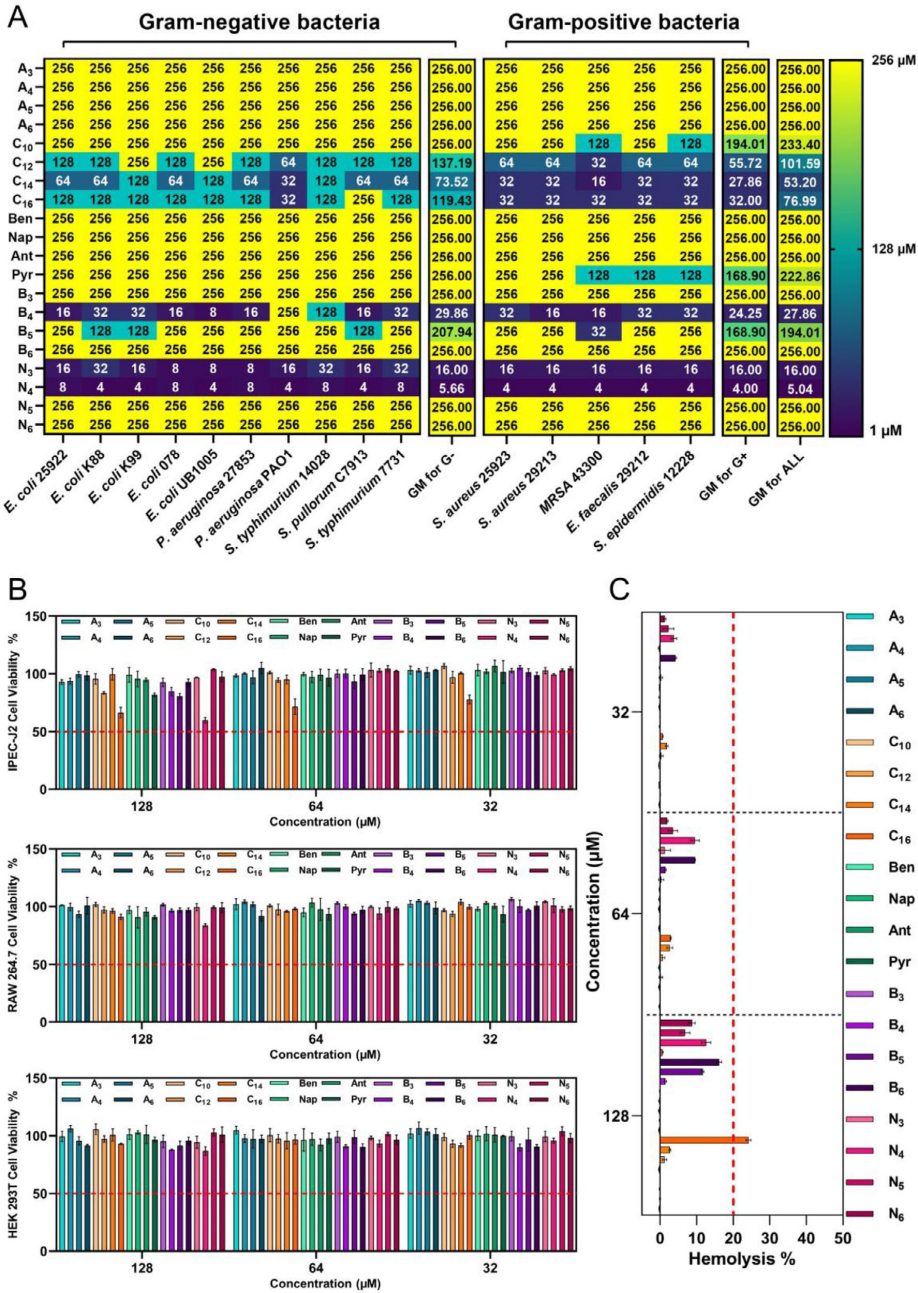
In vitro antimicrobial activity and biocompatibility of nano‐short peptides. A) Minimum inhibitory concentration (MIC) and GM_MIC_ values of nano‐short peptides against Gram‐negative bacteria and Gram‐positive bacteria. A value of 256 indicates no detectable antimicrobial activity under the test conditions (*n* = 3). B) The cytotoxicity of nano‐short peptides to IPEC‐J2, RAW264.7, and HEK 293 T cells, the baseline used for evaluation was set at 50%. C) Hemolytic activity of nano‐short peptides, and the baseline for assessment was set at 20%. Data in (B) and (C) are the mean ± SD; *n* = 3.

The minimum inhibitory concentration (MIC) was employed to evaluate the antimicrobial efficacy of short nano‐short peptides (**Figure** **3**A), with the GM_MIC_ providing a visual assessment of their antimicrobial activity against both Gram‐negative and Gram‐positive bacteria. Given that the short peptide sequence “R^D^RRP” provided a consistent positive charge across all nano‐short peptides, the terminal hydrophobic modification was crucial in determining their antimicrobial activity. The A_n_‐series template peptides did not exhibit antibacterial activity against both Gram‐negative and Gram‐positive bacteria, indicating that the shorter alkyl chains of alanine side chains yielded limited hydrophobic driving force for membrane disruption. The complete substitution of alanine with fatty acids resulted in an increase in the antibacterial activity of nano‐short peptides, demonstrating a tendency to increase and then decrease as the length of fatty acid chains was increased, with C_14_ exhibiting the most potent antibacterial effect (GM_MIC_ = 53.20 × 10^−6^ m). This suggests that exceeding a certain hydrophobicity threshold could decrease antibacterial activity. Furthermore, as hydrophobicity increased, all the Cn‐series nano‐short peptides displayed smaller GM_MIC_ values for Gram‐positive bacteria compared to Gram‐negative bacteria, indicating greater antibacterial efficacy against. This observation suggested that hydrophobicity plays a more significant role in the mechanism of action of AMPs against Gram‐positive bacteria.^[^ ^33^ ^]^ Differences in membrane architecture between Gram‐negative and Gram‐positive bacteria account for distinct charge and hydrophobicity requirements for AMPs to exert their bactericidal effects. The membrane surface of Gram‐negative bacteria is characterized by a highly negatively charged lipopolysaccharides (LPS) layer,^[^ ^34^ ^]^ accounting for increased sensitivity to AMPs with strong positive charges. Conversely, Gram‐positive bacteria have a cell wall surface rich in weakly negative lipoteichoic acid (LTA),^[^ ^35^ ^]^ suggesting that the efficacy of AMPs against Gram‐positive bacteria may be more dependent on increased hydrophobicity. Interestingly, the substitution of alanine with aromatic hydrophobic groups failed to enhance the antimicrobial activity of nano‐short peptides. Even after the introduction of pyrene groups containing four benzene rings, Pyr showed weak antimicrobial activity (GM_MIC_ = 222.86 × 10^−6^ m), suggesting that the direct introduction of an aliphatic linear hydrophobic structure in the peptide sequence may be more effective for enhancing antimicrobial activity compared to the aromatic cyclic hydrophobic structure. During the design of tail anchoring modifications for alanine, both butyl‐anchoring and aromatic naphthyl‐anchoring could enhance the antimicrobial activity of nano‐short peptides. However, the activity of these peptides, like those with fatty acid substitutions, also followed a pattern of initial enhancement followed by weakening as the number of coupled butyl‐alanine or naphthyl‐alanine increased, further confirming the presence of a hydrophobicity threshold for achieving optimal antimicrobial activity with these short nano‐short peptides. Besides the hydrophobic threshold, we propose that the self‐assembly characteristics influence the antimicrobial activity of nano‐short peptides. Moderate hydrophobicity facilitates optimal self‐assembly, potentially enhancing the local concentration of the peptides on bacterial membranes. Conversely, excessive hydrophobicity can lead to over aggregation, which may hinder the interaction between nano‐short peptides and bacteria, thereby diminishing antimicrobial activity,^[^ ^36^ ^]^ an effect observed with peptides such as Ant, Pyr, B_5_, B_6_, N_5_, and N_6_. This structure‐activity relationship accounts for the low GM_MIC_ values observed for C_14_, B_4_, and N_4_ across different modification strategies. Among nano‐short peptides tested, C_12_, C_14_, C_16_, B_4_, N_3_, and N_4_ demonstrated detectable antimicrobial activity (GM_MIC_ ≤ 128 × 10^−6^ m), with N_4_ exhibiting high efficacy against both Gram‐negative and Gram‐positive bacteria (GM_MIC_ = 5.04 × 10^−6^ m).

The potential toxicity of peptide‐based antimicrobial materials to mammalian cells represents a major challenge. To address this, the biocompatibility of nano‐short peptides was systematically evaluated by assessing their cytotoxicity against mouse macrophages (RAW 264.7), human embryonic kidney cells (HEK 293 T), and intestinal porcine epithelial cells (IPEC‐J2). Besides, their hemolytic activity against human red blood cells (HRBCs) was assessed. As shown in Figure 3B, even at the highest concentration tested (128 × 10^−6^ m), all three mammalian cell types maintained over 50% viability. The geometric mean of IC_50_ (the lowest peptide concentration required to induce at least 50% cell death) is presented in Figure S5A (Supporting Information). Although long‐chain fatty acids are often associated with increased cytotoxicity in AMPs,^[^ ^37^ ^]^ C_16_ did not exhibit high cytotoxicity in the present study. This observation may be attributed to its overall lower positive charge, indicating that the potential increase in toxicity during AMP design can be effectively regulated by the balance of hydrophobicity and positive charge. In addition, as illustrated in Figure 3C, the hemolytic activity of the nano‐short peptides demonstrated an upward trend with increasing concentration. The HC_20_ values, which represent the lowest peptide concentration required to induce 20% hemolysis of erythrocytes, were used as a baseline to quantitatively evaluate this activity (Figure S5B, Supporting Information). Notably, only C_16_ induced more than 20% hemolysis at a concentration of 128 × 10^−6^ m, which may be attributed to its excessive hydrophobicity, which facilitated deeper penetration of AMPs into the phospholipid bilayer, leading to a loss of membrane selectivity.^[^ ^38^ ^]^ To systematically quantify the selectivity of the nano‐short peptides, we calculated their selectivity index (SI) based on IC_50_, HC_20_, and GM_MIC_ values. As depicted in Figure S5C (Supporting Information), the nanomodification strategy substantially enhanced the therapeutic potential of the short peptides, with the GM_SI_ values for C_12_ (GM_SI_ = 2.52), C_14_(GM_SI_ = 4.81), C_16_ (GM_SI_ = 2.8), B_4_ (GM_SI_ = 9.19), N_3_ (GM_SI_ = 16), and N_4_ (GM_SI_ = 50.8) exhibiting more than a twofold increase compared to the A_n_ series of template peptides. Overall, tail anchoring of hydrophobic groups demonstrated superior outcomes compared to direct alanine substitution. Notably, N_3_ and N_4_ exhibited substantial application potential, suggesting that the nanoscale modification achieved via naphthyl‐tail anchoring successfully optimized the balance between biocompatibility and antibacterial efficacy.

### 2.3 Screening of Efficient Nano‐Short Peptides Under Simulated Physiological Barriers

**Figure 4:**
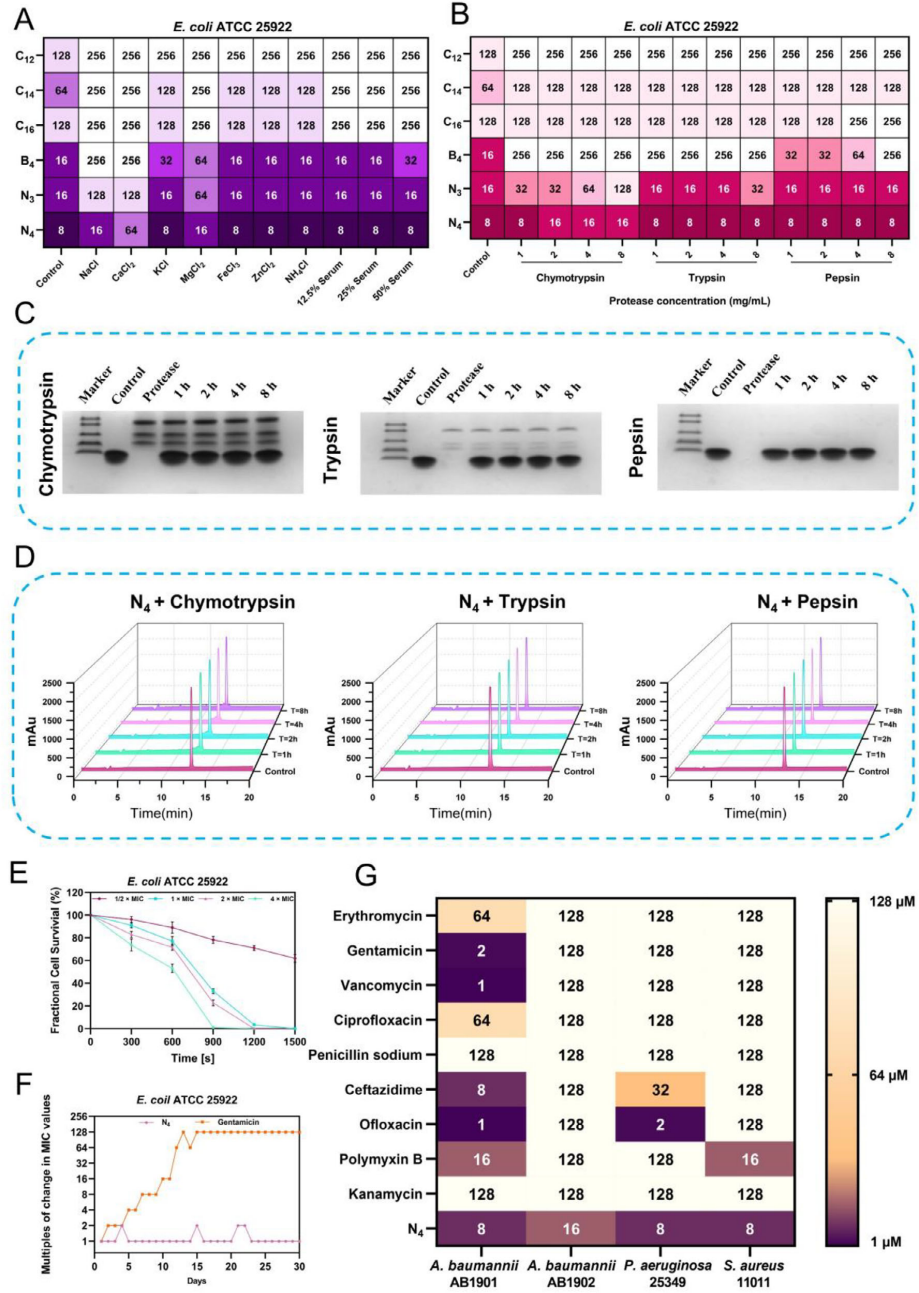
In vitro barrier penetration and antimicrobial properties of nano‐short peptides. A) The minimum inhibitory concentration (MIC) values of nano‐short peptides against *E. coli* ATCC 25922 in the presence of physiological salts and serum. A value of 256 indicates no detectable antimicrobial activity under the test conditions (*n* = 3). B) MIC values of nano‐short peptides against *E. coli* ATCC 25922 after incubation for 1 h with different concentrations (1, 2, 4, 8 mg mL^−1^) of various proteases. A value of 256 indicates no detectable antimicrobial activity under the test conditions (*n* = 3). C) Cleavage of N_4_ by various proteases. The peptides were incubated with protease for 0, 1, 2, 4, and 8 h at 37 °C, and the molecular weights of the protein markers from top to bottom were 31, 14.4, 7.8, 5.8 and 3.3 kDa. D) RP‐HPLC analysis of N_4_ after incubation with various proteases at a final concentration of 8 mg mL^−1^ for 0, 1, 2, 4, and 8 h. E) Time‐kill kinetic curves of *E. coli* ATCC 25922 after treatment with different concentrations of N_4_. Data are the mean ± SD; *n* = 3. F) The drug resistance curves of *E*. *coli* ATCC 25922 to N_4_ and gentamicin during a 30‐day continuous induction period. G) MIC values of N_4_ and antibiotics against clinical strains. A value of 128 indicates no detectable antimicrobial activity at a concentration of 64 × 10^−6^ m, a value of 1 indicates the antimicrobial activity is less than 2 × 10^−6^ m (*n* = 3).

The clinical application of antimicrobial agents is typically hindered by complex physiological conditions, such as the presence of salt ions and serum components. Therefore, the antimicrobial activity of nano‐short peptides was assessed against representative Gram‐negative bacterium *E. coli* ATCC 25922 and Gram‐positive bacterium *S. aureus* ATCC 29213 under physiological concentrations of salt ions and high concentrations of serum to initially assess their effectiveness under physiological conditions. As shown in **Figures** **4**A and S6 (Supporting Information), the antimicrobial activity of nano‐short peptides was significantly impaired by Na^+^ and Ca^2+^, while other ions yielded a weaker effect. Notably, the increased terminal hydrophobicity enhanced the stability of nano‐short peptides against salt ionic interference. Building upon these findings, it can be inferred that the increased hydrophobicity facilitated deeper penetration into the bacterial cell membrane, thereby enhancing their bactericidal efficacy.^[^ ^39^ ^]^ Furthermore, the charge‐shielding effect of Na^+^ and the competitive interaction of Ca^2+^ with peptide cations interfered with the ability of AMPs to recognize and bind to the negatively charged components of bacterial membranes, which were considered to be major factors contributing to the ion sensitivity of AMPs.^[^ ^40^, ^41^ ^]^ Increasing the net positive charge of AMPs has been identified as an effective strategy to enhance their ionic stability in saline environments.^[^ ^42^ ^]^ Although the increased hydrophobicity of nano‐short peptide sequences did not alter their overall net charge, it promoted self‐aggregation to some extent. This self‐aggregation may increase localized charge density by concentrating cationic residues, thereby counteracting salt ion interference, which conferred enhanced antimicrobial activity of nano‐short peptides at physiological concentrations of salt ions.

Based on serum stability assays (Figure 4A and Figure S6, Supporting Information), the antimicrobial efficacy of fatty acid‐substituted C_n_‐series nano‐short peptides was significantly compromised in the presence of serum. Even at the lowest serum concentration tested (12.5%), these peptides lost antimicrobial activity (MIC > 128 × 10^−6^ m), which may result from competitive binding mediated by serum albumin, which could bind to the fatty acids on peptides through hydrophobic interactions.^[^ ^43^, ^44^, ^45^ ^]^ In a serum environment, the electrostatic attraction between the nano‐short peptides (C_12_, C_14_, C_16_), which have only three positive charges, and bacteria may be weaker than the hydrophobic binding between fatty acids with serum albumin. The alanine tail‐anchored peptides, specifically B_4_, N_3_, and N_4_, retained their original antimicrobial capacity even after 4 h of incubation with various serum concentrations, showing their excellent serum stability.

This study was primarily designed to develop AMPs with enhanced resistance to protease hydrolysis. Therefore, the protease stability of the nano‐short peptides was initially evaluated by determining the MIC values against *E. coli* ATCC 25922 and *S. aureus* ATCC 29213 following 1 h of incubation with various proteases under different concentrations. As shown in Figure 4B and Figure S7 (Supporting Information), the C_n_‐series nano‐short peptides exhibited substantial protease resistance and were not severely affected by proteases, suggesting that replacing natural amino acids with fatty acids enhances the protease stability of AMPs. However, the butyl‐anchored B4 only exhibited limited resistance to low concentrations of pepsin, exhibiting complete loss of its antimicrobial activity (MIC > 128 × 10^−6^ m) at higher pepsin concentrations (8 mg mL^−1^) and even the lowest concentration (1 mg mL^−1^) of trypsin or chymotrypsin. In contrast, the naphthyl‐anchored N_3_ and N_4_ exhibited exceptional protease stability. The enhanced stability of N_3_ and N_4_ may be attributed to two potential mechanisms. First, the bulky, cyclic structure of the naphthyl group may create a steric hindrance that blocks proteases from recognizing and cleaving the peptide.^[^ ^25^ ^]^ Besides, the propensity of N_3_ and N_4_ to form nanostructures by self‐assembly could shield protease recognition sites. To systematically assess the effect of exposure time to proteases on the structural integrity of the nano‐short peptides, N_4_ was chosen for detailed analysis based on its superior antimicrobial activity across various protease conditions. The 16.5% tricine‐sodium dodecyl sulfate‐polyacrylamide gel electrophoresis (SDS‐PAGE) and RP‐HPLC images of N_4_ after exposing to various proteases (at a final concentration of 8 mg mL^−1^) for different times are shown in Figure 4C,D. Even after 8 h of protease treatment, N_4_ still maintained highly similar electrophoretic bands and RP‐HPLC chromatograms compared to untreated controls, demonstrating remarkable structural stability against protease hydrolysis.

The efficient antimicrobial activity exhibited by N_4_ in the presence of salt ions, serum, and proteases prompted us to further evaluate its clinical applicability. The bactericidal efficiency of N_4_ was initially assessed by a bactericidal kinetic assay (Figure 4E and Figure S8, Supporting Information). At a concentration of 1 × MIC, N_4_ eliminated all *E. coli* ATCC 25922 cells within 1500 s and all *S. aureus* ATCC 29213 cells within 3000 s, thereby demonstrating its potent bactericidal activity. The potential for resistance development of N_4_ was subsequently assessed through repeated passaging (Figure 4F and Figure S9, Supporting Information). After 30 successive passages at sub‐MIC concentrations, the MIC values for N_4_ against *E. coli* ATCC 25922 and *S. aureus* ATCC 29213 remained largely unchanged. In contrast, the antibiotic control, gentamicin, exhibited a 128‐fold increase in MIC values, suggesting that N_4_ exhibits a lower propensity for resistance development. In addition, as shown in Figure 4G, N_4_ demonstrated potent antimicrobial activity against clinically isolated strains, with MIC values ≤ 16 × 10^−6^ m. Moreover, it exhibited superior broad‐spectrum efficacy compared to conventional antibiotics, further demonstrating its potential application value as peptide‐based antimicrobial materials in a broader context. Based on these results, N_4_ was identified as the optimal nano‐short peptide for subsequent studies.

### 2.4 Comprehensive Investigation of the Self‐Assembly Properties of Optimal Nano‐Short Peptide N_4_

**Figure 5:**
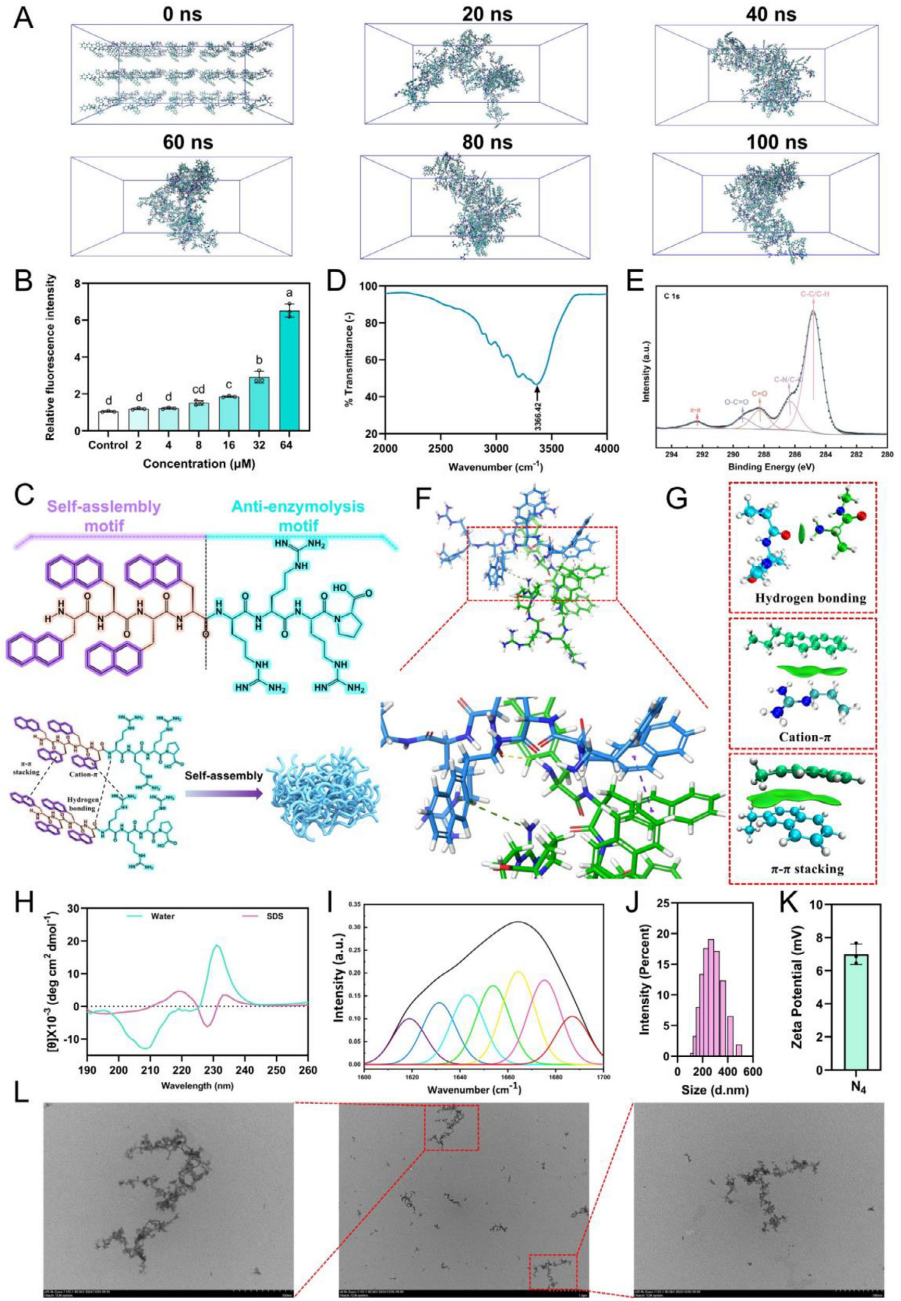
Further nano‐characterization analysis of N_4_. A) Simulated image of self‐assembly behavior of N_4_ within 100 ns obtained by molecular dynamics (MD). B) Thioflavin T (THT) fluorescence analysis of N_4_. Differences between groups were analyzed by one‐way ANOVA followed by Tukey's multiple comparisons tests. Values with different superscripts (a, b, c, and d) indicate a significant difference (*p <* 0.05). B) Tht fluorescence imaging of N_4_ at different concentrations. C) Structural formula and schematic illustration of the self‐assembly of N_4_. D) Fourier transform infrared (FTIR) spectra of N_4_. E) C 1s spectrum of N_4_ in X‐ray photoelectron spectroscopy (XPS). F) Simulated images of multiple intermolecular forces between N_4_ molecules obtained by MD. G) Independent Gradient Model based on Hirshfeld (IGMH) pictures of N_4_ molecular fragments. H) CD spectra of N_4_ in water and sodium dodecyl sulfate (SDS) solution. I) Amide I band in the FTIR spectrum of N_4_. J) Dynamic light scattering (DLS) and K) zeta potential determination measurement of N_4_. L) Transmission electron microscopy (TEM) negative staining images of N_4_. Scale bars, 500 nm and 1 µm. Data in (B), (J), and (K) are the mean ± SD; *n* = 3. N_4_ was used for detection at a concentration of 16 × 10^−6^ m in (J–L).

First, molecular dynamics (MD) simulations were conducted to visualize the self‐assembly of N_4_ in an aqueous environment. As illustrated in **Figure** **5**A, N_4_ molecules, initially dispersed, exhibited aggregation tendencies by 20 ns, ultimately reached a stable assembled state by 100 ns. Furthermore, as the simulation progressed, both the radius of gyration (Rg) and solvent‐accessible surface area (SASA) of N_4_ demonstrated a gradual decline, while the root mean square deviation (RMSD) and the number of hydrogen bonds increased and eventually stabilized (Figure S10, Supporting Information), which indicated increased cohesion between N_4_ molecules, leading to a denser and more stable assembly.^[^ ^32^ ^]^ Fluorescence analysis using 1,8‐ANS revealed that N_4_ exhibited the tendency to form oligomers at a concentration of 5.78 × 10^−6^ m (Figure S4, Supporting Information). Further nano‐characterization of N_4_ was conducted to explore its potential self‐assembly properties. Thioflavin T (ThT), a common indicator for amyloid conformation,^[^ ^46^, ^47^ ^]^ was used to assess fibril formation. N_4_ induced a significant increase in ThT fluorescence intensity at a concentration of 16 × 10^−6^ m (Figure 5B), indicating the formation of amyloid fibrils, which was further confirmed by ThT imaging of N_4_ (Figure S11, Supporting Information). Next, the relationship between the molecular composition of N_4_ and the formation of its nanofiber structure was explored. As shown in Figure 5C, the hydrophobic naphthyl‐alanine and hydrophilic short peptide anti‐enzymolysis motif “R^D^RRP” sequences were strategically distributed on both sides of the sequences in accordance with the characteristics of the surfactant structure. As the concentration of N_4_ increased, its amphiphilic structure promoted the aggregation of hydrophobic naphthyl‐alanine, providing the initial driving force for the self‐assembly of N_4_ into nanofibers. Moreover, previous studies have demonstrated that multiple intermolecular interactions collectively contribute to forming stable nanostructures of AMPs.^[^ ^48^ ^]^ As shown in Figure 5C, it can be inferred that a combination of various intermolecular forces drives the aggregation of N_4_ molecules, including π–π stacking between aromatic rings, cation‐π interactions between aromatic rings and arginine residues, and hydrogen bonding. These combined interactions drive the aggregated naphthyl‐alanine to extend in a parallel orientation along the main axis of the peptide sequence, ultimately leading to the formation of the nanofiber structure. To further validate our hypothesis, the fluorescence of N_4_ was first examined in an aqueous solution containing SDS (a hydrophobic interaction disruptor) to validate the importance of hydrophobic interactions in the self‐assembly process.^[^ ^49^ ^]^ As shown in Figure S12 (Supporting Information), the fluorescence spectra of N_4_ showed no concentration‐dependent surge, confirming the absence of molecular aggregation. This demonstrates SDS disrupts hydrophobic interactions, abolishing N4's self‐assembly capacity. Subsequently, the chemical bonding state of N_4_ was examined utilizing Fourier transform infrared spectroscopy (FTIR) and X‐ray photoelectron spectroscopy (XPS). As illustrated in Figure 5D, the FTIR spectrum of N_4_ displayed a distinct broad peak around 3400 cm^−1^, which provided evidence for water‐mediated hydrogen bonding (O‐H···N/O‐H···O).^[^ ^28^, ^50^ ^]^ Furthermore, XPS analysis revealed the presence of various C groups in N_4_, including C‐C/C‐H at 284.8 eV, C‐N/C‐O at 286.3 eV, C = O at 288.27 eV, and a π–π transition peak at 292.39 eV^[^ ^28^ ^]^ (Figure 5E). To further validate the dominant role of intermolecular forces in N_4_ self‐assembly, an integrated computational approach was employed, combining MD simulations, Independent Gradient Model based on Hirshfeld partition (IGMH) analysis, and density functional theory (DFT) calculations. The MD simulations initially revealed the above three key interactions: π–π stacking, cation‐π interactions, and hydrogen bonding (Figure 5F). Subsequent IGMH analysis visually confirmed these interactions through a color‐coded isosurface map (Figure 5G). As shown in Table S2 (Supporting Information), quantitative DFT calculations further characterized these interactions, revealing substantial binding energies for π–π stacking (−7.29 kcal mol^−1^), cation‐π interactions (−5.96 kcal mol^−1^), and hydrogen bonding (−2.77 kcal mol^−1^). Collectively, these comprehensive results provide compelling evidence for the presence of distinct intermolecular interactions in the self‐assembly behavior of N_4_. In addition to the molecular composition, the ordered secondary structure is widely thought to contribute to the formation of nanostructures.^[^ ^51^ ^]^ We preliminarily explored the secondary structure composition of N_4_ by CD spectroscopy to comprehensively reveal the self‐assembly mechanism of N_4_ (Figure 5H). In an aqueous environment, N_4_ displayed characteristic CD signals with a negative peak near 208 nm and a positive band near 195 nm, indicating the presence of a mixed *α*‐helix and *β*‐sheet conformation. We hypothesized that the helical structure conferred by the *α*‐helix conformation promotes the initial aggregation of N_4_ molecules, likely driven by hydrophobic interactions.^[^ ^48^ ^]^ Conversely, the *β*‐sheet conformation, characterized by a potentially parallel arrangement, is thought to enhance the ductility of the N_4_ molecules, thereby promoting the formation of fibrous structures. Furthermore, the naphthyl tail‐anchoring group induced a distinct positive peak near 230 nm of N_4_ in both aqueous and SDS (simulating the negatively charged bacterial surface) environments, confirming contributions of aromatic residues to the overall conformation.^[^ ^52^ ^]^ FTIR analysis offered more comprehensive and detailed secondary structure parameters, enabling systematic investigation of the potential correlation between the conformational architecture of N_4_ and its self‐assembly behavior into nanostructure formation. As shown in Figure 5I and Table S3 (Supporting Information), the secondary structure composition for N_4_ within the amide I band at different wavelengths were as follows: parallel *β*‐sheet (1618–1640 cm^−1^, 22.37%); random coil (1640–1650 cm^−1^, 14.44%); *α*‐helix (1650–1660 cm^−1^, 16.38%); *β*‐turn (1660–1670 cm^−1^, 19.30%); antiparallel *β*‐sheet (1670–1690 cm^−1^, 27.51%). Within the complex conformational composition of N_4_, both antiparallel and parallel *β*‐sheet structures were predominant, indicating that these *β*‐sheet conformations provide the primary structural support and combine various other secondary structures to synergistically promote the formation of amyloid fibril assemblies. Subsequently, we characterized the nanomorphology of N_4_ at a concentration of 16 × 10^−6^ m. Dynamic light scattering (DLS) measurements indicated that the aggregated N_4_ molecules were distributed within the size range of 100 to 600 nm (Figure 5J). Zeta potential analysis showed that their potential was 6.99 mV, confirming their net positive surface charge, which is likely essential for their binding to anionic microbial membranes through electrostatic attraction (Figure 5K). Furthermore, negative‐staining transmission electron microscopy (TEM) imaging confirmed the formation of N_4_ fiber structures (Figure 5L). Collectively, the above results demonstrate the feasibility of the self‐assembly system, which was driven by naphthyl tail anchoring.

### 2.5 Investigation of the Overall Antimicrobial Mechanism of Nano‐Short Peptides

**Figure 6:**
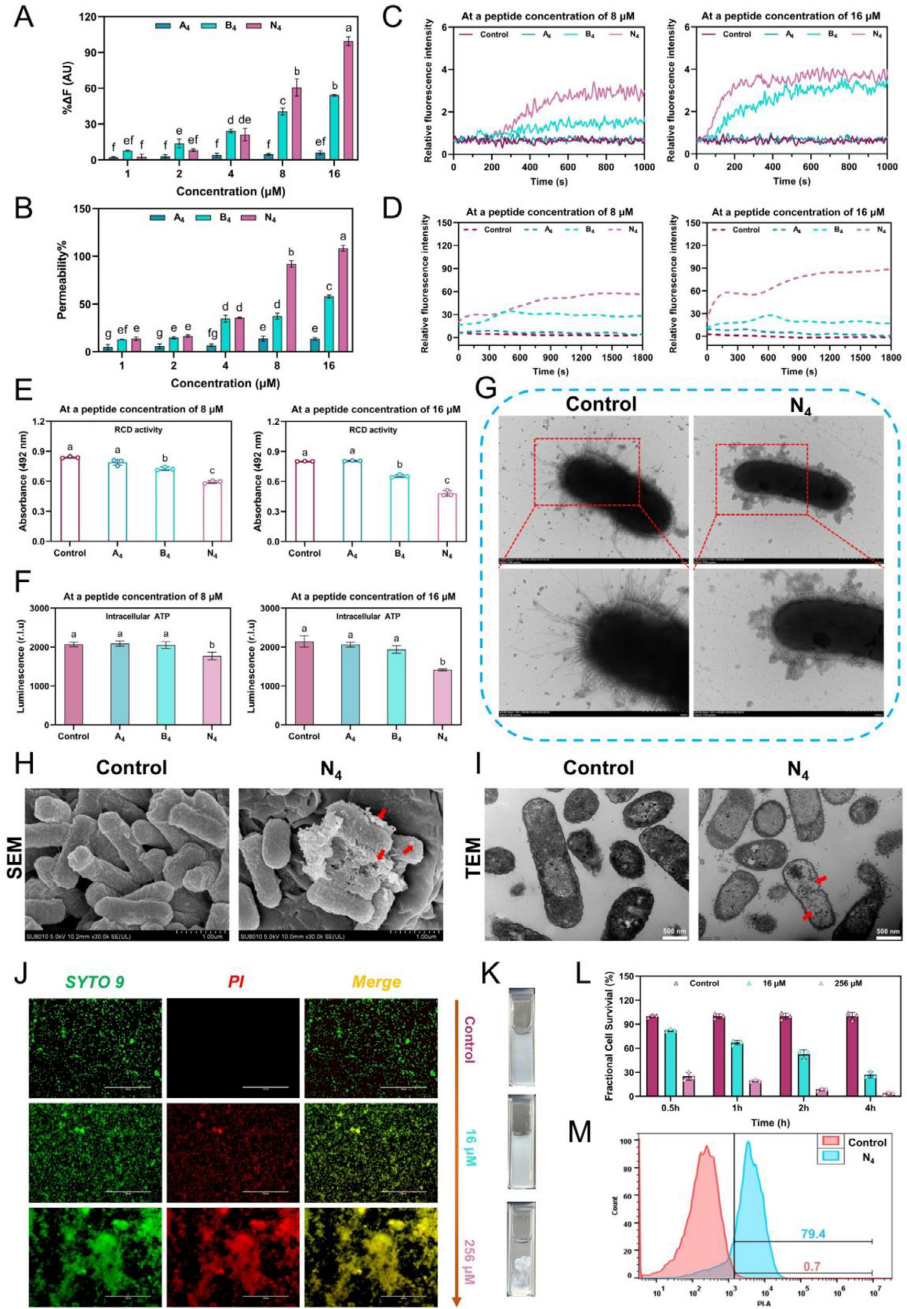
Antimicrobial mechanism of nano‐short peptides. A) Lipopolysaccharides (LPS) binding affinities of A_4_, B_4_, and N_4_. B) Effect of A_4_, B_4_, and N_4_ on the outer membrane permeability of *E. coli* ATCC 25922. C) Effect of A_4_, B_4_, and N_4_ on the cell membrane integrity of *E. coli* ATCC 25922 at different concentrations. D) Depolarization ability of A_4_, B_4_, and N_4_ on cytoplasmic membrane of *E. coli* ATCC 25922 at different concentrations. E) Inhibition on respiratory chain dehydrogenase activity of *E. coli* ATCC 25922 by A_4_, B_4_, and N_4_ at different concentrations. F) Intracellular ATP content in *E. coli* ATCC 25922 cells after treatment with different concentrations of A_4_, B_4_, and N_4_. Differences between groups in (A), (B), (E) and (F) were analyzed by one‐way ANOVA followed by Tukey's multiple comparisons tests. Values with different superscripts (a, b, c, and …g) indicate a significant difference (*p <* 0.05). Data are the mean ± SD; *n* = 3. G) Transmission electron microscopy (TEM) negative staining images of *E. coli* ATCC 25922 treated with N_4_. Scale bars, 500 nm and 1 µm. H) Scanning electron microscopy (SEM) images of *E. coli* ATCC 25922 treated with N_4_ Scale bars, 1µm. I) TEM images of *E. coli* ATCC 25922 treated with N_4_. Scale bars, 500 nm. J) Live/dead fluorescence imaging of *E. coli* ATCC 25922 after N_4_ treatment at different concentrations. Scale bars, 100 µm. K) The agglutination of *E. coli* ATCC 25922 in cuvette induced by different concentrations of N_4_. L) Bacterial content of the *E. coli* ATCC 25922 supernatant in cuvettes treated with different concentrations of N_4_. M) Flow cytometry imaging of *E. coli* ATCC 25922 after N_4_ treatment. N4 was used for detection at a concentration of 16 × 10^−6^ m in (G), (H), (I), and (M).

*Escherichia coli* ATCC 25922 and *S. aureus* ATCC 29213 were selected as representative strains to investigate the potential bactericidal mechanisms of nano‐short peptides (A_4_, B_4_, and N4), and probe the effect of butyl and naphthyl tail anchoring. Consistent with established mechanisms of cationic amphiphilic antimicrobials, the initial bacterial membrane targeting of nano‐short peptides may be achieved through electrostatic interactions with negatively charged ionic components, such as LPS or LTA, on the bacterial surface.^[^ ^53^ ^]^ As shown in **Figure** **6**A and S13A (Supporting Information), although A_4_, B_4_, and N_4_ possessed the same number of positive charge, their distinct hydrophobic group tail anchoring endowed them with different binding abilities for LPS and LTA, suggesting that hydrophobicity plays a crucial role in the recognition of negative ionic components on bacterial surfaces by peptide‐based antimicrobial materials. N_4_ and B_4_ demonstrated increased LPS‐binding capacity with higher concentrations. At a concentration of 8 × 10^−6^ m, B_4_ and N_4_ achieved 40.51% and 60.67% of polymyxin B's binding efficiency, respectively, while the corresponding values at 16 × 10^−6^ m were 54.24% and 99.47%. The enhanced affinity of N_4_ may be attributed to its self‐assembly into oligomers, which concentrated its positive charges and facilitated more effective interactions with LPS. Interestingly, B_4_ demonstrated enhanced LTA‐binding affinity relative to N_4_, with its binding capacity attaining 85.77% and 96.79% of the antibiotic control at concentrations of 8 × 10^−6^ and 16 × 10^−6^ m, respectively, while the corresponding values for N_4_ were 65.60% and 73.08%. This phenomenon was attributed to the structural differences between LPS and LTA. In contrast to the highly negatively charged LPS, LTA possessed weak anionic character,^[^ ^54^ ^]^ which may result in cationic antimicrobials binding to LTA with significantly less efficiency than LPS through electrostatic attraction. Consequently, oligomer formation by N_4_ failed to substantially enhance its LTA‐binding capacity. Furthermore, the presence of nonpolar hydrophobic glycolipid chains in LTA suggested that hydrophobic interaction may be a potential mode of binding to LTA in addition to electrostatic attraction.^[^ ^55^ ^]^ Thus, the anchoring of aliphatic butyl could have increased the lipophilicity of B_4_, thereby enhancing its ability to bind LTA through hydrophobic interactions. Utilizing the hydrophobic fluorescent probe N‐phenyl‐1‐naphthylamine (NPN), the impact of nano‐short peptides was investigated on the bacterial outer membrane of *E. coli* ATCC 25922 and the cell wall of *S. aureus* ATCC 29213.^[^ ^56^ ^]^ As demonstrated in Figure 6B and Figure S13B (Supporting Information), A_4_ exhibited limited membrane‐disrupting effects against both the outer membrane and the cell wall, likely due to its insufficient hydrophobicity. In contrast, B_4_ and N_4_ displayed concentration‐dependent permeabilization effects (1 × 10^−6^–16 × 10^−6^ m) following their binding to LPS and LTA. Compared to polymyxin B, B_4_ exhibited moderate outer membrane disruption activity against *E. coli* ATCC 25922, achieving 37.37% efficacy at 8 × 10^−6^ m and 57.94% at 16 × 10^−6^ m. In contrast, N_4_ demonstrated significantly stronger activity with 92.06% disruption at 8 × 10^−6^ m and 108.41% at 16 × 10^−6^ m. For cell wall permeabilization in *S. aureus* ATCC 29213, using ciprofloxacin as the reference, B_4_ achieved 19.49% and 37.80% permeabilization at 8 × 10^−6^ and 16 × 10^−6^ m, while the corresponding values for N_4_ were 48.48% and 110.54%, respectively. Notably, the superior disrupting efficacy of N_4_ compared to B_4_ may be attributed to the larger size of the naphthyl within the N_4_ sequence or the higher local concentration effects resulting from self‐assembly. Building upon the demonstrated efficacy of nano‐short peptides in disrupting bacterial surface barriers at concentrations of 8 and 16 × 10^−6^ m, we further investigated their membrane‐perturbing mechanisms. Since propidium iodide (PI) dyes can stain nucleic acids by entering cells through compromised membrane structures, they were employed to evaluate the impact of nano‐short peptides on bacterial cell membrane integrity.^[^ ^14^ ^]^ As shown in Figure 6C and Figure S13C (Supporting Information), B_4_ and N_4_ elicited a time‐dependent enhancement of PI dye fluorescence in *E. coli* ATCC 25922 and *S. aureus* ATCC 29213 within a 1000 s interval, suggesting progressive disruption of the bacterial membrane structure. Furthermore, compared with 8 × 10^−6^ m, a more pronounced fluorescence enhancement was induced at 16 × 10^−6^ m, thereby corroborating their dose‐dependent characteristics. Following the induction of membrane damage, the nano‐short peptides effectively permeated into the bacterial cell interior. Thus, the fluorescent probe DiSC_3_‐5 was employed to assess their impact on the bacterial cytoplasmic membrane (CM) potential. As illustrated in Figure 6D and Figure S13D (Supporting Information), A_4_ exhibited limited ability to induce CM depolarization, aligning with its limited membrane interaction properties. Conversely, B_4_ and N_4_ were found to enhance the relative fluorescence intensity of DiSC_3_‐5 in *E. coli* ATCC 25922 and *S. aureus* ATCC 29213 within 1800 s. Consistent with the findings on membrane integrity, they exhibited dose‐dependent effects, with N_4_ displaying a more pronounced depolarization‐inducing capability at equivalent concentrations. This finding underscored the ability of B_4_ and N_4_ to alter potential and disrupt ionic homeostasis by inducing CM depolarization.^[^ ^57^ ^]^ It is well‐established that the β‐galactosidase‐mediated hydrolysis of o‐nitrophenyl β‐D‐galactopyranoside (ONPG) serves as a quantitative indicator of CM integrity in *E. coli* ATCC 25922.^[^ ^58^ ^]^ As depicted in Figure S14 (Supporting Information), only N_4_ induced an increase in absorbance at 420 nm (approximately twofold at 8 × 10^−6^ m and fivefold at 16 × 10^−6^ m) within 1800 s, indicating its rapid capacity to disrupt the CM of *E. coli* ATCC 25922. In contrast, B_4_ exhibited limited membrane‐disrupting ability, likely due to its reduced penetration efficiency through the double‐membrane structure characteristic of Gram‐negative bacteria.

In addition to the direct membrane disruption, peptide‐based antimicrobial agents may exert bactericidal effects by interfering with energy metabolism.^[^ ^30^ ^]^ Given that the bacterial respiratory chain was located within the cell membrane, the disruption of cell membrane by nano‐short peptides inspired us to explore their effects on bacterial respiration. Red tetrazolium (RT) dye can be reduced to red by respiratory chain dehydrogenase (RCD).^[^ ^59^ ^]^ This assay was used to evaluate the activity of RCD in *E. coli* ATCC 25922 and *S. aureus* ATCC 29213 cells following treatment with nano‐short peptides. The significant decrease in 420 nm absorbance observed in Figure 6E and Figure S13E (Supporting Information) provided compelling evidence that B_4_ and N_4_ could effectively suppress the RCD activity of *E. coli* ATCC 25922 and *S. aureus* ATCC 29213. Specifically, at a concentration of 16 × 10^−6^ m, B_4_ reduced RCD activity to 70.75% in *E. coli* ATCC 25922 and 34.12% in *S. aureus* ATCC 29213, while N_4_ exhibited significantly stronger inhibition, decreasing RCD activity to 60.10% and 18.84% in the corresponding strains. This suggests that nano‐short peptides interfere with bacterial respiration by affecting RCD activity after acting on the cell membrane, which may induce potential disorders of energy metabolism. Prompted by the observed inhibition of bacterial respiration, we focused on the alterations in ATP content within the bacteria, given that ATP represents an important product of the respiration process. As shown in Figure 6F and Figure S13F (Supporting Information), N_4_ significantly reduced ATP production in *E. coli* ATCC 25922 and *S. aureus* ATCC 29213, while B_4_ only affected *S. aureus* ATCC 29213. At a concentration of 16 × 10^−6^ m, N_4_ decreased ATP levels to 66.07% in *E. coli* ATCC 25922 and 10.8% in *S. aureus* ATCC 29213, whereas B_4_ reduced ATP levels in *S. aureus* ATCC 29213 to 71.18%. The observed lack of efficacy of B_4_ in suppressing intracellular ATP production in *E. coli* ATCC 25922 may stem from its limited ability to induce CM permeability. Furthermore, the excessive accumulation of reactive oxygen species (ROS) in both *E. coli* ATCC 25922 and *S. aureus* ATCC 29213 cells, resulting from impaired respiration and dysregulated energy metabolism, served as a key indicator of bacterial cell death. As illustrated in Figure S15 (Supporting Information), compared with untreated bacterial samples, at a concentration of 16 × 10^−6^ m, B_4_ increased ROS content by 1.51‐fold in *E. coli* ATCC 25922 and 1.38‐fold in *S. aureus* ATCC 29213, whereas N_4_ increased it by 2.26‐fold and 1.83‐fold, respectively. Collectively, our findings suggested that A_4_ demonstrated limited antibacterial efficacy due to insufficient hydrophobicity. In contrast, B_4_ and N_4_ exhibited synergistic antibacterial effects by disrupting membrane integrity and energy metabolism, with naphthyl tail anchoring further enhancing the potency of N_4_.

Encouraged by the significant antimicrobial effect demonstrated by N_4_ at a concentration of 16 × 10^−6^ m, we visualized its direct impact on bacteria by microimaging at this concentration, aiming to further verify its multiple antimicrobial mechanisms. Negative TEM staining of N_4_‐treated *E. coli* ATCC 25922 and *S. aureus* ATCC 29213 (Figure 6G and Figure S13G, Supporting Information) revealed that N_4_ adhered to the cell surfaces as fibrous aggregates, thereby confirming a preliminary mechanism of membrane interaction. Subsequently, scanning electron microscopy (SEM) and TEM imaging provided compelling evidence for the membrane‐damaging effect of N_4_. As illustrated in Figure 6H and Figure S13H (Supporting Information), SEM analysis revealed that treatment with N_4_ at a concentration of 16 × 10^−6^ m induced wrinkles, ruptures, and irregular vesicle protrusions on the membrane surfaces of *E. coli* ATCC 25922 and *S. aureus* ATCC 29213, in contrast with the intact membrane architecture with a smooth cellular membrane surface of the control group. As shown in Figure 6I and Figure S13I (Supporting Information), TEM imaging further demonstrated that untreated control cells maintained a dense internal structure with their cellular contents intact. However, exposure to N_4_ at a concentration of 16 × 10^−6^ m resulted in compromised membrane integrity, thinning of the internal structure, and leakage of intracellular contents, resulting in the formation of cavities within *E. coli* ATCC 25922 and *S. aureus* ATCC 29213. The results of the live/dead staining analysis are shown in Figure 6J and Figure S13J (Supporting Information). Under fluorescence microscopy, the observation of chartreuse fluorescence indicated that the red signal emitted by PI had merged with the nucleic acid dye SYTO 9, thereby further confirming the membrane damage induced by N_4_. Quantitative fluorescence analysis using ImageJ determined the red (PI)/green (SYTO 9) fluorescence ratio at various N_4_ concentrations to quantify bacterial mortality rates. Specifically, treatment with 16 × 10^−6^ m N_4_ resulted in fluorescence overlap rates of 79.11% for *E. coli* ATCC 25922 and 90.74% for *S. aureus* ATCC 29213, while at 256 × 10^−6^ m, the overlap rates increased to 99.29% and 98.65%, respectively. Furthermore, *E. coli* ATCC 25922 and *S. aureus* ATCC 29213 treated with 256 × 10^−6^ m N_4_ exhibited significant fluorescence aggregation, suggesting potential bacterial agglutination. Peptide‐based nanostructures have been established to achieve effective bacterial eradication by mediating agglutination,^[^ ^32^, ^60^, ^61^ ^]^ a promising bactericidal mechanism. The phenomenon of bacterial agglutination mediated by N_4_ in the cuvette was shown in Figure 6K and Figure S13K (Supporting Information). In contrast with the homogeneous bacterial suspensions observed in both control and 16 × 10^−6^ m N_4_‐treated samples, exposure to 256 × 10^−6^ m N_4_ induced rapid sedimentation of both *E. coli* ATCC 25922 and *S. aureus* ATCC 29213 within 1 h, resulting in visibly clarified supernatants. Moreover, the decrease in supernatant bacterial load confirmed that bactericidal efficacy was enhanced at elevated N_4_ concentrations through aggregation‐mediated mechanisms (Figure 6L and Figure S13L, Supporting Information). Furthermore, as shown in Figure 6M and Figure S13M (Supporting Information), flow cytometry analysis revealed the potent membrane‐disrupting activity of N_4_, with 16 × 10^−6^ m treatment increasing PI‐positive proportions from 0.7% to 79.4% in *E. coli* ATCC 25922 and from 1.23% to 36.7% in *S. aureus* ATCC 29213.

In summary, the nano‐short peptides achieved initial binding through recognition of negatively charged components on bacterial surfaces, subsequently facilitating peptide penetration, and ultimately leading to the inhibition of energy metabolism. Notably, while both butyl and naphthyl tail‐anchored modifications effectively enhanced the membrane‐interacting efficacy of nano‐short peptides, the naphthyl‐anchored N_4_ demonstrated superior antibacterial potency, attributed to the combined effects of naphthyl's intrinsic physicochemical properties and its propensity for self‐assembly. These findings comprehensively elucidate the multiple antimicrobial mechanisms of N_4_, which may account for its reduced susceptibility to resistance development and highlight its potential as a promising peptide‐based nano‐antimicrobial agent.

### 2.6 Evaluation of In Vivo Biocompatibility and Treatment of Bacterial Infection

**Figure 7:**
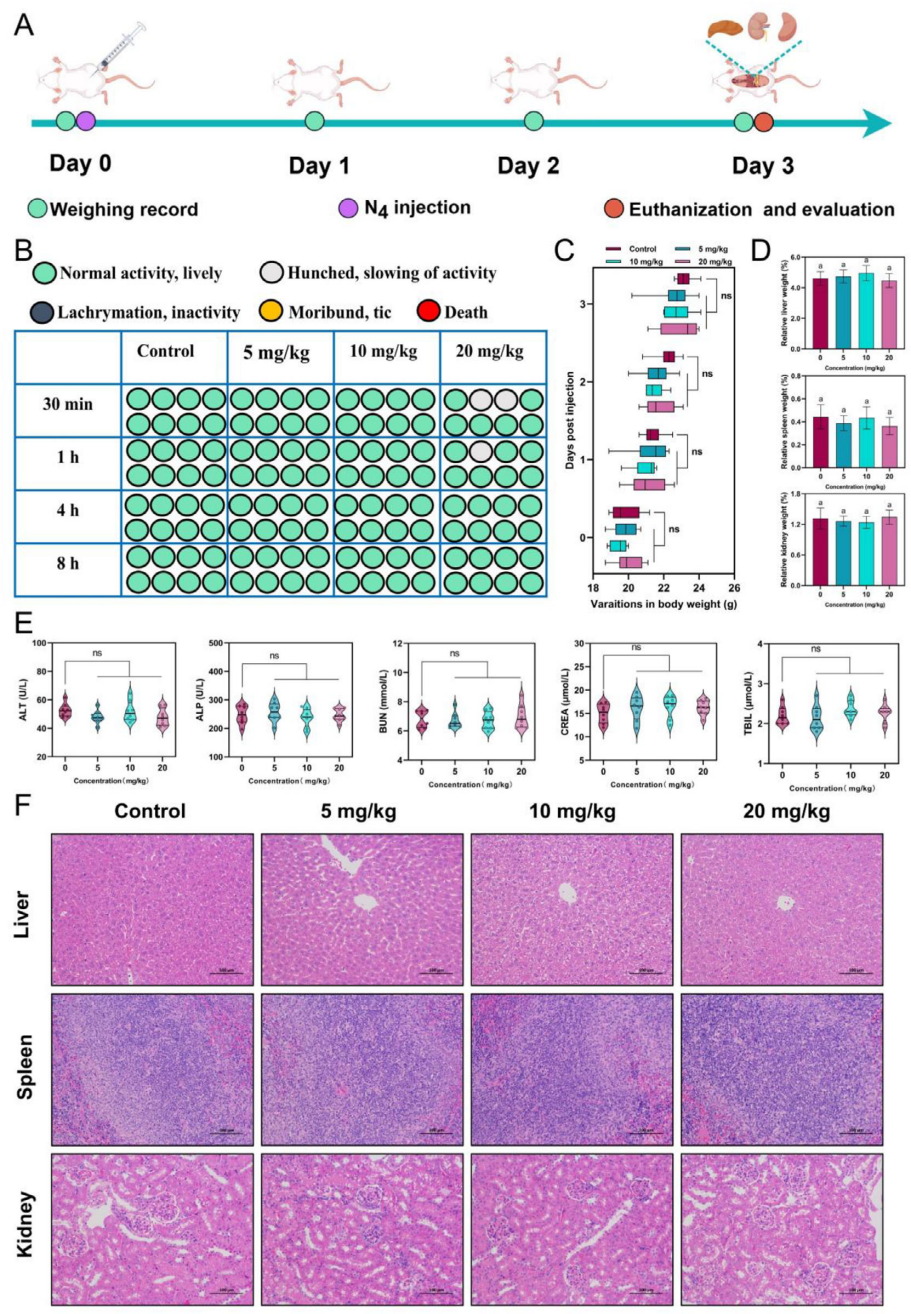
N_4_ exhibits excellent biocompatibility in vivo. A) In vivo toxicity assessment experimental workflow. B) Body condition scores of mice at 8 h after treatment with different concentrations of N_4_. C) Changes in body weight curves and D) relative organ weights of liver, spleen, and kidney in mice treated with different concentrations of N_4_ (*p >* 0.05). E) Various parameters of liver and kidney function were assessed in mice injected with N_4_ at doses of 5, 10, or 20 mg kg^−1^ and saline. Statistically, there were no significant variances between the experimental groups (*p >* 0.05). F) Histopathological H&E staining of the liver, spleen, and kidney of mice after intraperitoneal injection of different concentrations of N_4_. Scale bars, 100 µm. Differences between groups were analyzed by one‐way ANOVA followed by Tukey's multiple comparisons tests. Data are the mean ± SD; *n* = 8.

The excellent antimicrobial efficacy and multiple mechanisms exhibited by N_4_ in vitro prompted us to investigate its in vivo efficacy and assess its potential applications. Initially, the potential toxicity was evaluated through intraperitoneal injection of N_4_ at varying concentrations (5, 10, 20 mg kg^−1^) into ICR female mice (**Figure** **7**A), with saline treatment serving as the control group. The effects of different doses of N_4_ on the mice were assessed by monitoring alterations in clinical signs and body weight. As illustrated in Figure 7B, only two mice in the 20 mg kg^−1^ N_4_‐treated group displayed mild and transient symptoms, including stooping, hunching, and reduced mobility within 1 h. Furthermore, no significant differences in body weight were observed between the N_4_‐treated and control groups (*p* > 0.05) (Figure 7C). Subsequently, we conducted a comprehensive investigation into the effects of N_4_ on the relative organ weights, blood biochemical indices, and histological analysis of mouse organs to systematically evaluate its biocompatibility in vivo. As shown in Figure 7D, the relative weights of the liver, spleen, and kidney among mice treated with varying doses of N_4_ remained comparable (*p* > 0.05). Blood biochemical analysis further revealed no significant differences in the levels of alanine aminotransferase (ALT), alkaline phosphatase (ALP), blood urea nitrogen (BUN), creatinine (CREA), and total bilirubin (TBIL) between the N_4_‐treated group and the control group (*p* > 0.05), suggesting that N_4_ did not cause liver or kidney dysfunction (Figure 7E). Furthermore, as shown in Figure 7F, neatly organized liver cords, uniform distribution of red and white pulp in the spleen, and morphologically normal glomeruli and renal tubules were observed in mice treated with different doses of N_4_, which provided compelling evidence that N_4_ did not inflict histopathological damage to the liver, spleen, or kidney. Overall, the above results confirmed the excellent in vivo biocompatibility of N_4_, suggesting its potential for clinical application as a peptide‐based nano‐antimicrobial agent.

**Figure 8:**
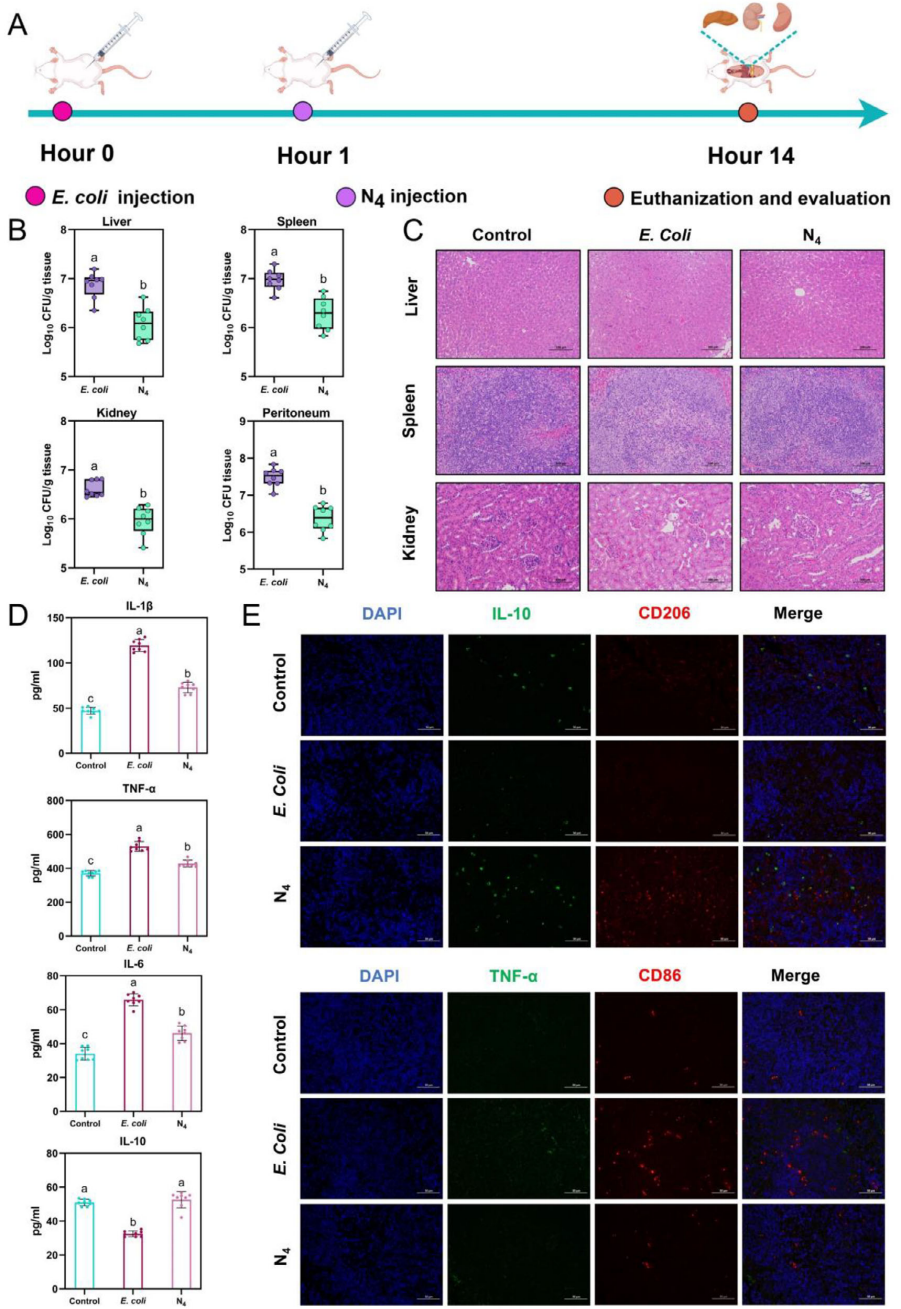
N_4_ exhibits excellent efficacy in vivo. A) In vivo efficacy assessment experimental workflow. B) Bacterial load of *E. coli* in the liver, kidney, and spleen following infection with *E. coli* and subsequent treatment with saline and N_4_. Differences between groups were analyzed using independent *t*‐tests, values with different superscripts indicate a significant difference (*p <* 0.05). Data are the mean ± SD; *n* = 8. C) Histopathological H&E staining of the liver, spleen, and kidney. Scale bars, 100 µm. D) Levels of inflammatory factors (IL‐10, IL‐1β, IL‐6, and TNF‐α) in the serum of mice from different treatment groups. Values with different superscripts (a, b, and c) indicate a significant difference (*p <* 0.05). Differences between groups were analyzed by one‐way ANOVA followed by Tukey's multiple comparisons tests. Data are the mean ± SD; *n* = 8. E) Fluorescence images of M1 macrophage biomarker (TNF‐α, CD86) and M2 macrophage biomarker (IL‐10, CD206) in spleen of mice subjected to different treatments. Scale bars, 50 µm.

Given the negligible in vivo toxicity of N_4_, its potential against in vivo bacterial infections was further explored by evaluating its effectiveness in a mouse model of *E. coli*‐induced peritonitis‐sepsis. Prior to this, the stability of N_4_ was first evaluated using serum isolated from ICR female mice. The results (Figure S16, Supporting Information) demonstrated that N_4_ maintained over 50% integrity after 4‐h incubation and retained an 8.1% undegraded fraction after 12 h, indicating its remarkable stability under physiological conditions. As illustrated in **Figure** **8**A, mice were initially treated with dilutions of *E. coli* (OD_600_ = 0.6, 100 µL) via intraperitoneal injection at 0 h. One hour after infection, the mice were subjected to different treatments (N_4_‐treated group: N_4_, 10 mg kg^−1^, 100 µL, *E. coli*‐infected group: saline, 100 µL). The control group received an equivalent volume of saline at each corresponding time point. After 14 h, the mice were euthanized, and the in vivo efficacy of N_4_ was comprehensively evaluated through bacterial quantification, analysis of serum inflammatory factors, histological analysis, and immunofluorescence analysis. The bacterial load was shown in Figure 8B: liver (6.87 log_10_ CFU g^−1^), spleen (6.97 log_10_ CFU g^−1^), kidney (6.62 log_10_ CFU g^−1^), and peritoneum (7.48 log_10_ CFU) for the *E. coli‐*infected group, while the corresponding values for the N_4_ treatment group were 6.07, 6.28, 5.96, and 6.37, respectively. N_4_ significantly reduced the bacterial load in the mouse tissues (*p* < 0.05), indicating its efficacy in combating bacterial infections in vivo. Furthermore, the effective clearance of invading bacteria by N_4_ alleviated tissue damage associated with *E. coli* infection. Histological analyses demonstrated that N_4_ treatment restored the organized structure of the hepatic portal vein, normalized the distribution of red and white pulp in the spleen, and reduced swollen renal tubules and inflammatory cell infiltration, in infected mice (Figure 8C). In addition, treatment with N_4_ exhibited potential immunomodulatory effects, characterized by significant decreased serum levels of pro‐inflammatory cytokines (IL‐1β, IL‐6, and TNF‐α) and increased levels of anti‐inflammatory cytokines IL‐10 (all *p* < 0.05) (Figure 8D). Subsequently, given the high correlation between spleen function and immune response, the spleen was selected for immunofluorescence analysis to further explore the above‐mentioned potential immunomodulatory mechanisms. As depicted in Figure 8E, immunofluorescence imaging revealed that N_4_ treatment elevated the expression of M2 macrophage markers (IL‐10 and CD206) while decreasing the expression of M1 macrophage markers (TNF‐α and CD86). This finding suggested that N_4_ could exert potential immunomodulatory effects by influencing macrophage polarization, thereby resisting bacterial invasion. Collectively, these results indicated that N_4_ could synergistically combat bacterial infections by integrating direct antibacterial activity with immunomodulatory properties.

**Figure 9:**
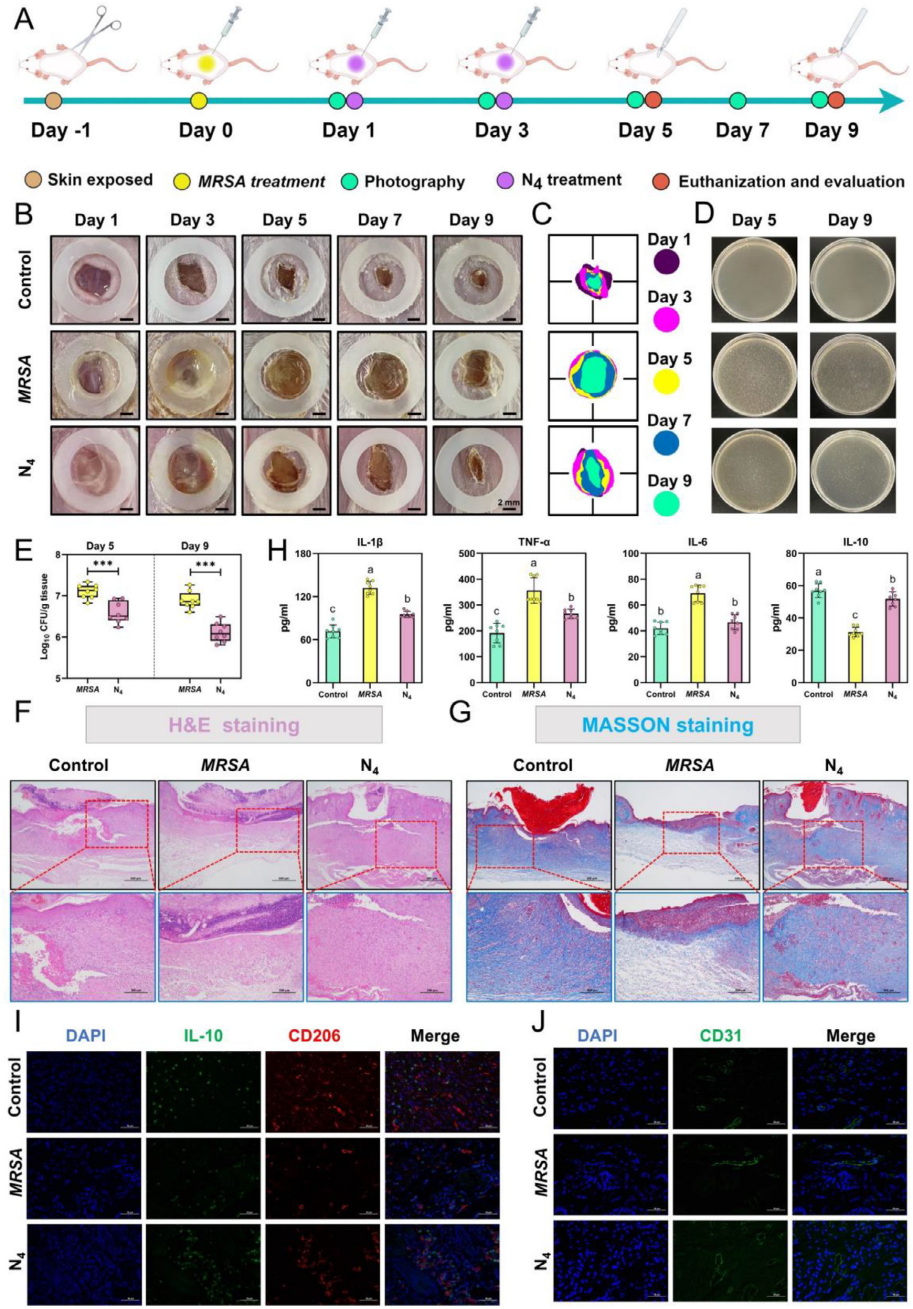
The therapeutic evaluation of N_4_ on *MRSA*‐mediated mouse skin model. A) *MRSA*‐mediated mouse skin model assessment experimental workflow. B) Images of wounds in mice subjected to different treatments within 9 days after model establishment. Scale bars, 2 mm. C) Traces of the corresponding wounds in mice after different treatments at different time points after model establishment. D) Representative pictures of bacteria isolated from the skin of infected mice in different treatment groups and E) statistics of bacterial loads. Differences between groups were analyzed using independent *t*‐tests, values with different superscripts indicate a significant difference (^*^ *p <* 0.05, ^**^ *p <* 0.01, ^***^ *p <* 0.001). Data are the mean ± SD; *n* = 8. F) Histopathological H&E staining and G) Masson staining of tissues collected from the abscess areas in different treatment groups at Day 9. Scale bars, 500 µm and 200µm. H) Levels of inflammatory factors (IL‐10, IL‐1β, IL‐6, and TNF‐α) in homogenates of mice skin tissues from different treatment groups. Values with different superscripts (a, b, and c) indicate a significant difference (*p <* 0.05). Differences between groups were analyzed by one‐way ANOVA followed by Tukey's multiple comparisons tests. Data are the mean ± SD; *n* = 8. I) Fluorescence images of M2 macrophage biomarker (IL‐10, CD206) and J) angiogenesis biomarker (CD31) in skin of mice subjected to different treatments. Scale bars, 50 µm.

To assess the broader potential application of N_4_ as a peptide‐based nano‐antimicrobial agent, we investigated its therapeutic efficacy in a MRSA‐infected wound model in mice. As shown in **Figure** **9**A, the mice underwent skin exposure treatment and acclimated for 24 h prior to wound creation. One day later (Day 0), a wound was created on the skin and inoculated dropwise with 10 µL of *MRSA* dilution (OD _600_ = 0.2), while control mice received an equal volume of saline. By Day 1 post‐infection, wounds of infected mice exhibited mucus and abscess formation, contrasting with the relatively clean wound sections observed in the control group (Figure 9B). Subsequently, infected mice received the corresponding treatments as follows: N_4_‐treated group (10 µL of N_4_, 5 mg kg^−1^, twice a day), and *MRSA*‐infected group (10 µL of saline, twice a day). By Day 3, N_4_ effectively inhibited further deterioration of wound abscesses caused by *MRSA* infection compared to the infected group (Figure 9B). The N_4_ treatment regimen was continued as previously described. By Day 5, the wounds in both the infected and N_4_‐treated mice exhibited scabbing, with a smaller scab area in the N_4_‐treated group. Figure 9C illustrates the progression of wound healing in mice 9 days post‐infection, demonstrating that N_4_ treatment effectively mitigated bacterial infection and accelerated wound healing. Additionally, N_4_ treatment led to a significant reduction in the invasive bacterial load in mouse skin tissue (*p* < 0.05), decreasing from 7.1 to 6.59 log_10_ CFU g^−1^ on Day 5 and from 6.89 to 6.12 log_10_ CFU g^−1^ on Day 9, which confirms the potent effect of N_4_ against bacterial infection. Subsequently, the potential for in vivo application of N_4_ was comprehensively evaluated through the analysis of inflammatory factors, H&E staining, Masson staining, and immunofluorescence on Day 9 post‐infection. Benefiting from the significant bactericidal effect of N_4_ on invading bacteria, histological analysis revealed reduced inflammatory infiltration and tissue damage associated with bacterial infection (Figure 9F). Masson staining further demonstrated significant collagen fiber deposition in the N_4_‐treated group compared to the *MRSA*‐infected group, indicative of the accelerated tissue healing (Figure 9G).^[^ ^62^ ^]^ In addition, as shown in Figure 9H, N_4_ treatment induced a significant decrease in the levels of pro‐inflammatory cytokines (IL‐1β, IL‐6, and TNF‐α) and a significant increase in the levels of the anti‐inflammatory cytokine IL‐10 in the supernatant fluid of the skin tissues (*p* < 0.05), suggesting its modulatory effect on inflammatory response mediated by bacterial infection. As illustrated in Figure 9I, the increased expression of M2 macrophage markers (IL‐10 and CD206) in the N_4_‐treated group demonstrated that the above modulatory effect may be achieved by inducing macrophage polarization, further underscoring the multiple mechanisms of N_4_ in combating bacterial infections in vivo. Furthermore, enhanced green fluorescence observed in the N_4_‐treated group compared with the *MRSA*‐treated group evidenced increased expression of the angiogenic marker CD31 (Figure 9J), representing increased angiogenesis and accelerated tissue repair during skin reconstruction in mice.

In conclusion, N_4_ has exhibited significant advantages in combating bacterial infections in vivo, characterized by negligible toxicity and a multifaceted mechanism that integrates direct bactericidal activity with potential immunomodulatory effects. These findings highlight its huge potential for application as a peptide‐based nano‐antimicrobial agent to address the escalating issue of bacterial drug resistance.

## 3 Conclusion

In conclusion, this study constructed a library of nano‐short peptides mediated by differential hydrophobic modifications, achieved either by executing direct replacement or tail‐anchored of alanine with unnatural hydrophobic groups. Notably, the self‐assembly system triggered by naphthyl tail‐anchored endows N_4_ with the ability to form nanofibrous structures, demonstrating broad‐spectrum antibacterial activity, high biocompatibility, and excellent tolerance to physiological conditions. Upon investigating its antibacterial mechanism, it was found that N_4_ primarily binds to negatively charged bacterial surface components and compromised the integrity of external barrier, ultimately inducing bacterial death through synergistic inhibition of energy metabolism. Furthermore, N_4_ exhibited significant efficacy in combating bacterial infections in both the *E. coli*‐induced peritonitis‐sepsis model and the *MRSA*‐mediated skin model in mice, which may be attributed to direct bacterial killing and potential immunomodulatory mechanisms. In summary, the combination of short peptide anti‐enzymolysis motif with nanosized modifications provides a promising solution to overcome the proteolytic susceptibility of AMPs, thereby advancing the development of peptide‐based nanomaterials to address the growing challenge of bacterial drug resistance.

## 4 Experimental Section

### Cell Culture and Bacterial Strains

The details of cell line and bacterial strains involved in the study are provided in the Supporting Information.

### Synthesis of Nano‐Short Peptides

Based on previous research,^[^ ^30^ ^]^ all the nano‐short peptides in this study were synthesized via solid‐phase synthesis by SynPeptide Co., Ltd. (Nanjing, China). The successful synthesis was verified by electrospray ionization mass spectrometry (ESI‐MS), with observed molecular masses matching the theoretical values. The synthesized nano‐short peptides were purified to more than 95% purity by liquid chromatography.

### Antimicrobial Activities

The MIC values of the nano‐short peptides were determined according to standard protocols.^[^ ^63^ ^]^ Bacterial strains were revived from cryopreservation and cultured in Mueller–Hinton broth (MHB) to logarithmic growth phase, then diluted to a final density of 0.5–1 × 10⁶ CFU mL^−1^. Serial dilutions of the nano‐short peptides were prepared in 0.2% bovine serum albumin (BSA) using a 96‐well plate format. Equal volumes of peptide solutions and bacterial suspensions were combined and incubated at 37 °C for 20–22 h. Bacterial growth was assessed by measuring absorbance at 492 nm, with MHB alone (negative control) and untreated bacterial suspension (positive control) included for reference. The MIC was defined as the lowest peptide concentration that completely inhibited bacterial growth (indicated by no increase in absorbance).

### Cytotoxicity Assays

The cytotoxicity of the nano‐short peptides was evaluated in three cell lines (RAW 264.7, HEK 293T, and IPEC‐J2) using the methylthiazolyldiphenyl‐tetrazolium bromide (MTT) assay.^[^ ^30^ ^]^ Cells were seeded in 96‐well plates at a density of 2 × 10⁵ cells per well, exposed to varying peptide concentrations, and incubated for 18–24 h (37 °C, 5% CO_2_). Next, 20 µL of MTT solution (0.5 mg mL^−1^) was added to each well, followed by 4 h of incubation. The supernatant was then aspirated, and formazan crystals were dissolved in 100 µL dimethyl sulfoxide (DMSO). Absorbance at 570 nm was measured using a microplate reader. Untreated cells served as the positive control, while cell‐free medium acted as the negative control. Cell viability (%) was calculated as: [(OD_570_ treated − OD_570_ negative control)/(OD_570_ positive control − OD_570_ negative control)] × 100%.

### Hemolysis Assays

The hemolytic activity of nano‐short peptides was evaluated using fresh HRBCs.^[^ ^30^ ^]^ HRBCs were resuspended with PBS (10 × 10^−3^ m, pH 7.4) by centrifugation (1000 × *g*, 10 min) and diluted to a 10% (v/v) suspension. Peptides were serially diluted in a 96‐well plate and mixed with an equal volume of HRBC suspension, followed by incubation at 37 °C for 1 h. Positive (0.1% Triton X‐100‐treated HRBCs) and negative (untreated HRBCs) controls were included. After centrifugation (1000 × *g*, 10 min), the supernatant was transferred to a new plate, and absorbance at 570 nm was measured by microplate reader (Tecan Infinite200 Pro). Hemolysis (%) was calculated as: [(OD_570_ treated − OD_570_ negative control)/(OD_570_ positive control − OD_570_ negative control)] × 100%.

### Salt and Serum Tolerance Assays

The antimicrobial stability of nano‐short peptides was assessed against model strains *E. coli* ATCC 25922 and *S. aureus* ATCC 29213 in physiological salts or serum environments.^[^ ^64^ ^]^ To examine salt tolerance, a test solution containing 0.2% BSA supplemented with physiological salt concentrations was prepared: 150 × 10^−3^ m NaCl, 4.5 × 10^−3^ m KCl, 6 × 10^−6^ m NH_4_Cl, 8 × 10^−6^ m ZnCl_2_, 1 × 10^−3^ m MgCl_2_, 2 × 10^−3^ m CaCl_2_, and 4 × 10^−6^ m FeCl_3_. The MIC values of nano‐short peptides were then determined in this solution to evaluate their antimicrobial efficacy in the presence of physiological salt ions.

The serum stability of nano‐short peptides was evaluated by incubating peptide solutions with fetal bovine serum (FBS), which was at concentrations of 25%, 50%, and 100% (diluted in 0.2% BSA solution). Following 4 h of incubation, the antimicrobial activity was determined by measuring the MIC against the test strains.

### Protease Stability Assays

The protease stability of the nano‐short peptides was evaluated using MIC determination, 16.5% tricine‐SDS‐PAGE, and RP‐HPLC analyses, in accordance with established methods.^[^ ^30^ ^]^ Nano‐short peptides solutions were mixed with proteases (trypsin, chymotrypsin, or pepsin) at different concentrations (2, 4, 8, and 16 mg mL^−1^) in equal volumes, followed by 1 h incubation at 37 °C. The protease stability of the nano‐short peptides was preliminarily evaluated by MIC testing against *E. coli* ATCC 25922 and *S. aureus* ATCC 29213.

To further evaluate the effect of protease exposure time on peptide structure, peptides solutions were incubated with 16 mg mL^−1^ protease solutions (equal volume mixing) at 37 °C for varying durations (1, 2, 4, and 8 h). Finally, 16.5% tricine‐SDS‐PAGE and RP‐HPLC were used to detect the structural integrity of the peptides.

### Drug Resistance Assays

The potential resistance development of nano‐short peptides was evaluated through 30 serial passages of *E. coli* ATCC 25922 and *S. aureus* ATCC 29213 in sub‐MIC conditions,^[^ ^38^ ^]^ using gentamicin as control. Bacterial cultures were adjusted to 0.5–1 × 10⁶ CFU mL^−1^ in MHB prior to each MIC determination.

### Time‐Kill Kinetics Assays

The logarithmic growth phase cultures of *E. coli* ATCC 25922 and *S. aureus* ATCC 29213 were adjusted to a density of 0.5–1 × 10⁶ CFU mL^−1^ in PBS (10 × 10^−3^ m, pH 7.4) and exposed to different concentrations of nano‐short peptides (1/2 × MIC, 1 × MIC, 2 × MIC, and 4 × MIC).^[^ ^63^ ^]^ Samples were collected at pre‐determined time points post‐exposure, serially diluted, and plated on Mueller‐Hinton agar (MHA) for viable colony counting after 18–24 h incubation at 37 °C. Untreated bacterial suspensions served as controls.

### CAC Values of Nano‐Short Peptides

The self‐assembly behavior of nano‐short peptides was characterized by determining the CAC using 1,8‐ANS as a hydrophobic fluorescent probe.^[^ ^32^ ^]^ Serial peptide dilutions (2‐256 × 10^−6^ m in 50 µL deionized water) were prepared in 96‐well plates. 1,8‐ANS powder was solubilized in dimethylformamide (DMF) and added to the 96‐well plates (final concentration of 1 × 10^−6^ m), and deionized water solution containing 1,8‐ANS was used as a blank control. After incubation for 1 h, the fluorescence emission spectra were continuously monitored with a microplate reader (Tecan Infinite200 Pro, *λ* excitation = 360 nm, *λ* emission = 420–670 nm). CAC values were determined by linear fitting of representative fluorescence intensities.

### ThT Fluorescence Assay and Staining

The fluorescent dye was used for fluorescence measurements and imaging of N_4._^[^ ^48^ ^]^ N_4_ was serially diluted (4‐128 × 10^−6^ m, 50 µL, each well) in deionized water using a 96‐well plate. An equal volume of ThT solution was added to each well (final concentration: 40 × 10^−6^ m), with ThT in deionized water serving as the control. After 20 min incubation at 37 °C, fluorescence intensity was measured using a microplate reader (excitation *λ* = 450 nm, emission *λ* = 480 nm). N_4_ was stained with 40 × 10^−6^ m ThT for 30 min and observed by imaging using a fluorescence microscope.

### CD Spectroscopy Assays

CD spectroscopy (190–260 nm) was employed to probe the secondary structure of N_4_ (150 × 10^−6^ m) in water or 30 × 10^−3^ m SDS environments. Measurements were performed on a Chirascan spectrometer in the United Kingdom, with subsequent conformational analysis using Shao's method.^[^ ^65^ ^]^

### Fourier Infrared Spectroscopy

The N_4_ sample and potassium bromide were mixed and ground in a ratio of 1:100, and the samples were pressed and tested using a Nicorette iS10 FT‐IR spectrometer, with a wave number range of 400–4000 cm^−1^, a spectrometer resolution of 4 cm^−1^, and a signal to mania ratio of 50 000:1, and scanned 32 times and analyzed by infrared spectroscopy.^[^ ^32^ ^]^

### Dynamic Light Scattering and Zeta Potential Analysis

N_4_ was diluted to 16 × 10^−6^ m using deionized water and added to disposable plastic cuvettes, and the kinetic diameters was determined using a Zetasizer Nano Z90 (Malvern Instruments, Worcestershire, U.K.).^[^ ^29^ ^]^ N_4_ was diluted to 16 × 10^−6^ m using deionized water and added to the electrode cups, and the zeta potential was determined using a Zetasizer Nano Z90 (Malvern Instruments, Worcestershire, UK).^[^ ^29^ ^]^

### Negative Staining Observation

N_4_ was diluted to 16 × 10^−6^ m using deionized water and aged overnight at room temperature, and the samples were placed on copper‐plated carbon grids and negatively stained with 1% phosphotungstic acid for 20s. The samples were observed by a Hitachi H‐7800 TEM and pictures were taken.^[^ ^48^ ^]^

### Molecular Dynamics (MD) Simulations

MD simulations of protein‐ligand complexes were performed using GROMACS 2020.3 software, visualizing, analyzing, and animating Trajectories with VMD 1.9.3 and PyMOL 2.4.1. The experimental methods used in this paper were referred to previous study.^[^ ^32^ ^]^

### XPS

The X‐ray photoelectron spectrometer (Thermo escalab, 250XI) was employed to scan the C 1s narrow spectrum, utilizing a monochromatic Al Kα radiation (*hν* = 1486.6 eV) with a power setting of 150 W. Charge correction was conducted by referencing the C 1s peak of adventitious carbon at 284.8 eV.^[^ ^28^ ^]^

### IGMH Analysis

Noncovalent interactions between N_4_ molecules were characterized using IGMH analysis with Multiwfn software, and visualized by VMD software.^[^ ^66^, ^67^ ^]^

### DFT Calculations

DFT‐based binding energy calculations were performed using Orca program at the wB97M‐V/def2‐TZVPP level with RI (Resolution of Identity) acceleration, and the basis set superposition error (BSSE) was compensated using gcp (dft/tz) method.^[^ ^68^ ^]^

### LPS and LTA Binding Assays

The binding ability of nano‐short peptides to LPS and LTA was evaluated using a BODIPY‐TR‐cadaverine (BC) displacement assay.^[^ ^59^ ^]^ Test solutions containing LPS or LTA (50 µg mL^−1^) were pre‐incubated with BC dye (5 µg mL^−1^) for 4 h in the dark. Peptide solutions at varying concentrations were then mixed with the LPS or LTA, polymyxin B (final concentration 10 µg mL^−1^) was used as a positive control, and LPS or LTA mixed with dye solution was used as a negative control. The fluorescence intensity (excitation *λ* = 580 nm, emission *λ* = 620 nm) was measured. %Δ*F* (au) = [(*F*_treated_ – *F*_negative control_)/(*F*_positive control_ – *F*_negative control_)] × 100%.

### Outer Membrane Permeability Assays

The NPN fluorescent probe was used to assess the permeability of the outer membrane.^[^ ^29^ ^]^ A logarithmic phase culture of *E. coli* ATCC 25922 bacterial solution was diluted to OD_600_ = 0.2 in a 5 × 10^−3^ m HEPES buffer (pH = 7.4 with 5 × 10^−3^ m glucose). Following the addition of NPN (final concentration: 10 × 10^−6^ m), the mixture was incubated for 30 min in the dark. Subsequently, the bacterial suspension was mixed with an equal volume of nano‐short peptides solution at different concentrations. Polymyxin B (10 µg mL^−1^) was used as a positive control, while an untreated bacterial dilution served as the negative control. Fluorescence intensity was measured (excitation *λ* = 350 nm, emission *λ* = 420 nm) using a microplate reader. The percentage of outer membrane permeability was calculated as: Permeability% = [(*F*_treated_ – *F*_negative control_)/(*F*_positive control_ – *F*_negative control_)] × 100%.

### Cell Wall Permeability Assays

The NPN fluorescent probe was used to assess the permeability of cell wall.^[^ ^64^ ^]^ A logarithmic phase culture of *S. aureus* ATCC 29213 bacterial solution was diluted to OD_600_ = 0.2 in a 5 × 10^−3^ m HEPES buffer (pH = 7.4 with 5 × 10^−3^ m glucose). Following the addition of NPN (final concentration: 10 × 10^−6^ m), the mixture was incubated for 30 min in the dark. Subsequently, the bacterial suspension was mixed with an equal volume of nano‐short peptides solution at different concentrations. Ciprofloxacin (10 µg mL^−1^) was used as a positive control, while an untreated bacterial dilution served as the negative control. Fluorescence intensity was measured (excitation *λ* = 350 nm, emission *λ* = 420 nm) using a microplate reader. The percentage of outer membrane permeability was calculated as: Permeability% = [(*F*_treated_ – *F*_negative control_)/(*F*_positive control_ – *F*_negative control_)] × 100%.

### Cell Membrane Integrity

The effect of nano‐short peptides on the integrity of bacterial cell membranes was assessed using the fluorescent probe PI.^[^ ^38^ ^]^ Logarithmic growth stage *E. coli* ATCC 25922 and *S. aureus* ATCC 29213 bacterial solutions were diluted to OD_600_ = 0.2 in PBS (pH = 7.4, 10 × 10^−3^ m). The PI dye was subsequently added (at a final concentration of 10 µg mL^−1^), and untreated bacterial dilutions were used as controls. After incubation for 15 min, the fluorescence changes were continuously monitored from 0 to 1000 s using a microplate reader (excitation *λ* = 535 nm, emission *λ* = 615 nm).

### Cytoplasmic Membrane Depolarization Assays

The ability of nano‐short peptides to induce depolarization of the bacterial cytoplasmic membrane was assessed by the membrane potential‐sensitive probe DiSC_3_‐5.^[^ ^58^ ^]^ Logarithmic growth stage *E. coli* ATCC 25922 and *S. aureus* ATCC 29213 bacterial solutions were diluted to OD_600_ = 0.05 in 5 × 10^−3^ m HEPES buffer (pH = 7.4 with 20 × 10^−3^ m glucose) and incubated for 1 h with DiSC_3_‐5 (final concentration of 0.4 × 10^−6^ m). Next, a saturated KCl solution (final concentration of 100 × 10^−3^ m) was added and incubated for another 30 min at 37 °C. Finally, the incubated mixture was mixed with different concentrations of nano‐short peptides solutions in equal volumes, and then the fluorescence changes (excitation *λ* = 622 nm, emission *λ* = 670 nm) were continuously monitored from 0 to 3000 s using a microplate reader.

### Cytoplasmic Membrane Permeability Assays

The ONPG hydrolysis assay was employed to determine the effects of nano‐short peptides on permeability of *E. coli* ATCC 25922 cytoplasmic membranes. The detailed experimental procedures followed the previously published methodology.^[^ ^30^ ^]^

### Respiratory Chain Dehydrogenase Viability Assays

The inhibitory effects of nano‐short peptides on RCD activity in bacteria were evaluated using an RT reduction assay.^[^ ^58^ ^]^ Logarithmic growth stage *E. coli* ATCC 25922 and *S. aureus* ATCC 29213 bacterial solutions were diluted to OD_600_ = 0.4 in MHB liquid medium. A mixed solution was prepared by combining RT (1 mg mL^−1^), glucose (100 × 10^−3^ m), and Tris‐HCl buffer (50 × 10^−3^ m, pH 8.6) in equal volumes (1:1:1). In 96‐well microplates, serial dilutions of nano‐short peptides were prepared using the mixed solution, followed by addition of an equal volume of bacterial suspension. Following incubation at 37 °C for 1.5 h, the absorbance at 492 nm was measured using a microplate reader, with untreated bacterial suspensions serving as controls.

### Intracellular ATP Determinations

The intracellular ATP levels of *E. coli* ATCC 25922 and *S. aureus* ATCC 29213 after nano‐short peptides treatment were detected using an Enhanced ATP Assay Kit.^[^ ^59^ ^]^ Logarithmic growth stage *E. coli* ATCC 25922 and *S. aureus* ATCC 29213 bacterial solutions were diluted to OD_600_ = 0.4 in MHB liquid medium. Then, the bacterial dilutions were co‐incubated with different concentrations of nano‐short peptides at 37 °C for 1 h. After incubation, the bacterial dilutions were centrifuged (12 000 rpm, 5 min, 4 °C) to pellet the cells. The resulting bacterial pellets were processed according to the steps in the kit. Finally, the chemiluminescence was detected by a microplate reader.

### Reactive Oxygen Species Generation

ROS accumulation levels were assessed by the fluorescent probe DCFH‐DA.^[^ ^63^ ^]^ Logarithmic growth stage *E. coli* ATCC 25922 and *S. aureus* ATCC 29213 bacterial solutions were diluted to OD_600_ = 0.4 in PBS (pH = 7.4, 10 × 10^−3^ m), followed by incubation with DCFH‐DA final concentration of 10 × 10^−6^ m) for 30 min at 37 °C. Subsequently, the bacterial suspensions were then mixed with different concentrations of nano‐short peptides dilutions in equal volumes in a 96‐well plate and continued to incubate for 1 h (37 °C). Finally, the fluorescence intensity was measured using a microplate reader (excitation *λ* = 488 nm, emission *λ* = 525 nm).

### Negative Staining Imaging Observation

Logarithmic growth stage *E. coli* ATCC 25922 and *S. aureus* ATCC 29213 bacterial solutions were diluted to OD_600_ = 0.2 in PBS (pH = 7.4, 10 × 10^−3^ m). Subsequently, N_4_ (final concentration of 16 × 10^−6^ m) was added and incubated for 1 min, followed by negative staining for 10 s using 0.1% phosphotungstic acid. Following air‐drying, the bacterial samples were observed using Hitachi H‐7800 TEM and representative pictures were taken.^[^ ^48^ ^]^

### Flow Cytometry

The effect of treatment with N_4_ (final concentration of 16 × 10^−6^ m) on *E. coli* ATCC 25922 and *S. aureus* ATCC 29213 was examined by flow cytometry.^[^ ^14^ ^]^ Logarithmic growth stage *E. coli* ATCC 25922 and *S. aureus* ATCC 29213 bacterial solutions were diluted to OD_600_ = 0.2 in PBS (pH = 7.4, 10 × 10^−3^ m). The bacterial samples were incubated with N_4_ for 1 h and then incubated with the PI dye (at a final concentration of 10 µg mL^−1^) for a further 15 min. Finally, bacteria containing PI dye were detected by FACScan flow cytometer at 488 nm excitation wavelength, bacterial solutions (containing PI dye) without N_4_ treatment were used as controls.

### SEM and TEM Characterizations

Logarithmic growth stage *E. coli* ATCC 25922 and *S. aureus* ATCC 29213 bacterial solutions were diluted to OD_600_ = 0.3 in PBS (pH = 7.4, 10 × 10^−3^ m). These bacterial solutions were exposed to N_4_ (final concentration of 16 × 10^−6^ m) for 1h (37 °C). Following this, the bacterial solutions underwent centrifugation (4 °C, 1000 rpm, 10 min), and the bacterial deposits were fixed overnight at 4 °C with 2.5% (w/v) glutaraldehyde. The subsequent examination of *E. coli* ATCC 25922 and *S. aureus* ATCC 29213 membranes for morphological was conducted using SEM (Hitachi su‐8010, Japan) and TEM (Hitachi H‐7650, Japan), following the procedures from the prior publication.^[^ ^14^ ^]^

### Live/Dead Staining Assays

The effect of N_4_ on *E. coli* ATCC 25922 and *S. aureus* ATCC 29213 was further assessed by fluorescent probes PI and SYTO 9.^[^ ^69^ ^]^ Logarithmic growth stage *E. coli* ATCC 25922 and *S. aureus* ATCC 29213 bacterial solutions was diluted to OD_600_ = 0.3 in PBS (pH = 7.4, 10 × 10^−3^ m), then N_4_ was mixed with bacterial solutions for 1 h, followed by the addition of SYTO 9 and PI dyes for another 30 min. Finally, a fluorescence microscope (EVOS FL Auto, Massachusetts, USA) was used to observe the fluorescence images of the bacteria under different background colors and representative pictures were taken.

### Bacterial Agglutination

The bacterial agglutination assay was performed as previously described, with minor modifications.^[^ ^64^ ^]^ Logarithmic growth stage *E. coli* ATCC 25922 and *S. aureus* ATCC 29213 bacterial solutions was diluted to OD_600_ = 0.2 in PBS (pH = 7.4, 10 × 10^−3^ m), and then bacterial solutions and different volumes of N_4_ were added to washed cuvettes. At different time points (0.5, 1, 2, 4 h), the supernatant in the cuvette was aspirated and evenly spotted on MHA solid medium for overnight incubation. Representative photographs of bacterial aggregates were taken (1 h).

### In Vivo Toxicity of N_4_

Animal experiments were conducted in accordance with the Guide for the Care and Use of Laboratory Animals at Northeast Agricultural University (NEAU‐[2011]‐9). The experimental subjects comprised female ICR mice, aged 6 to 8 weeks and weighing 19 ± 1.1 g, sourced from Liaoning Changsheng Biotechnology Co. Thirty‐two mice were randomly divided into four groups after a 3‐day pre‐feeding period, followed by intraperitoneal injections of N_4_ at different doses (5, 10, and 20 mg kg^−1^) to assess its toxicity, with saline treatment serving as the control group. Following injection, behavioral alterations were observed over a brief duration (0.5, 1, 4, and 8 h), and then body weight changes were monitored for 3 days. On the third day, the mice were euthanized via ether inhalation anesthesia, and blood samples were collected from the orbital vein for biochemical analysis, which was used to determine the levels of ALT, ALP, BUN, CREA, and TBIL. Subsequently, the liver, spleen, and kidneys were taken to determine organ coefficients. The relative organ weights were calculated using the formula: relative organ weight (%) = (organ weight/mouse body weight) × 100%. These organs were then fixed in 4% paraformaldehyde, following established protocols, for subsequent histological examination.^[^ ^70^ ^]^

### Metabolism of N_4_ in Serum

N_4_ was incubated with serum isolated from mice for different durations (0, 1, 2, 4, 8, 12 h), followed by RP‐HPLC analyses to determine the retention rate.^[^ ^48^ ^]^

### In Vivo Peritonitis–Sepsis Model

The mice used in the peritonitis‐sepsis model were consistent with those employed in the in vivo toxicity assessment. Twenty‐four mice were randomly divided into three groups after a 3‐day pre‐feeding period. The mice in the control group received intraperitoneal injections of 100 µL of saline, followed by an additional 100 µL of saline 1 h later. The mice in the other two groups were administered intraperitoneal injections of 100 µL of a saline‐diluted *E. coli* suspension (OD_600_ = 0.6), and subsequently received either 100 µL of saline (*E. coli*‐infected group) or 10 mg kg^−1^ of N_4_(N_4_‐treated group) 1 h later. 14 h post‐infection, the mice were euthanized via ether inhalation anesthesia, and blood samples were collected from the orbital vein for the analysis of inflammatory factors (IL‐1β, IL‐6, TNF‐α, and IL‐10).^[^ ^71^ ^]^ In addition, peritoneal fluid was aseptically collected via peritoneal lavage with sterile saline. The liver, spleen, and kidneys were harvested, weighed, and homogenized. Homogenized samples were plated on Mueller‐Hinton agar (MHA) solid medium for overnight incubation, and bacterial load was quantified the next day. Sections of the liver, spleen, and kidneys were obtained for histological analysis employing H&E staining. Besides, the spleens were specifically selected for immunofluorescence analysis to assess the expression levels of IL‐10, TNF‐α, CD206, and CD86.

### *MRSA*‐Mediated Skin Infection

The mouse skin infection model was based on previous research with some modifications.^[^ ^62^ ^]^ The mice utilized in the peritonitis‐sepsis model were consistent with those employed in the in vivo toxicity assessment. Forty‐eight mice were randomly divided into three groups after a 3‐day pre‐feeding period, including control, N_4_‐treated and *MRSA*‐infected groups. Initially, the dorsal regions of the mice were depilated (Day ‐1). On the following day (Day 0), circular incisions were made on the dorsal surfaces, and 10 µL of saline‐diluted MRSA suspension (OD_600_ = 0.2) was applied to the wounds of the mice in N_4_‐treated and *MRSA*‐infected groups, while an equivalent volume of saline was administered to the wounds in the control mice. After 24 h (Day 1), the N_4_‐treated mice received 10 µL of saline containing N_4_ (5 mg mL^−1^) applied to their skin wounds, whereas the infected and control groups of mice were treated with an equal volume of saline, and two treatments were carried out on the same day. The above treatments were repeated after 2 days (Day 3). Throughout the experimental period, representative images of the wounds were captured.

Mice were euthanized on Days 5 and 9 post‐infection, and skin tissue homogenates were quantified for bacterial load. Subsequently, the supernatants of skin tissue homogenates from mice on Day 9 were selected for the detection of inflammatory factors, and wound tissues were collected for histological, Masson and immunofluorescence analyses.

### Statistical Analysis

All in vitro tests were performed with at least three independent replicates and analyzed as well as plotted using GraphPad Prism 9.5.0 software. Differences between two groups were analyzed by *t*‐test. Differences between multiple groups were analyzed by one‐way ANOVA, and Tukey's method was used for multiple comparisons when the differences were significant. *p* > 0.05 was considered as nonsignificant and *p* < 0.05 was considered as significant (^*^ *p* < 0.05, ^**^ *p* < 0.01, ^***^ *p* < 0.001), and different letters (a, b, c, d, e, etc.) indicate significant differences (*p* < 0.05). The data were expressed as mean ± standard deviation (SD).

## Conflict of Interest

The authors declare no conflict of interest.

## Author Contributions

X.Y. and Y.L. contributed equally to this work. X.Y., Y.L., and A.S. formulated and planned this work. X.Y., Y.L., J.R., Y.Z., and L.Z. conducted the primary experimental assays. X.Y. completed the main writing of the manuscript. A.S. provided supervision and revisions to the final manuscript. All the authors have reviewed and approved the final version of the manuscript.

## Supporting information



Supporting Information

## Data Availability

The data that support the findings of this study are available from the corresponding author upon reasonable request.
