# Supplementary material for: Utilizing Proteolytic‐Resistant Nano‐Short Peptide Based on Naphthyl Tail‐Anchored to Combat Bacterial Infections
Source: Adv Sci (Weinh). 2025 Sep 15;12(45):e08854. doi: 10.1002/advs.202508854 (PMC12677699; doi:10.1002/advs.202508854)
Supplement: Supplementary file 1 — Supporting Information [file ADVS-12-e08854-s001.docx]

Supporting Information

**Utilizing Proteolytic-resistant Nano-short Peptide Based on Naphthyl Tail-anchored to Combat Bacterial Infections**

*Xi Yan†,* *Yinfeng Lyu†, Yi Liu, Jianping Ren, Yue Zhao, Licong Zhang,* *and Anshan Shan^*^*

College of Animal Science and Technology, Northeast Agricultural University, Harbin, 150030, The People’s Republic of China

^*^Address correspondence: [asshan@neau.edu.cn](mailto:asshan@neau.edu.cn)

Funding: This work was supported by the National Natural Science Foundation of China (32030101, 32272914 and U21A20252), the National Key R&D Program of China (2022YFD1300700), China Agriculture Research System (CARS-35) - Feeding management and breeding environment control.

†: *Xi Yan* and *Yinfeng Lyu* contributed equally to this work.

**Introduction:**

The Supporting Information consist of supporting text and supporting figures. The supporting text is used to describe the cell lines and bacterial strains utilized in the study. The supporting figures are used to illustrate content not detailed in the main text. Their primary components are as follows: Figure S1-S4 and Table S1 (Characterization of nano-short peptides), Figure S5 (The biological activity of nano-short peptides), Figure S6-S9 (In vitro barrier penetration and antimicrobial properties of nano-short peptides against *S. aureus* ATCC 29213), Figure S10-S12 and Table S2-S3 (Further nano-characterization analysis of N_4_), Figure S13-S15 (Antimicrobial mechanism of nano-short peptides against *S. aureus* ATCC 29213), Figure S16 (Half-Life Analysis of N_4_).Details are provided as follows:

Figure S1. Reverse high performance liquid chromatography (HPLC) spectra of nano-short peptides (P1-10); Figure S2. Mass spectrometry (MS) of nano-short peptides (P11-15); Figure S3. ANS fluorescence intensity changes and 3D structure diagram of nano-short peptides (P15-16); Figure S4. CAC values of nano-short peptides in aqueous solution (P17); Figure S5.GM of IC_50_, HC_20_ and GM of SI values for nano-short peptides (P17-19); Figure S6. The serum and salt ion stability of nano-short peptides against *S. aureus* ATCC 29213 (P19); Figure S7. The protease stability of nano-short peptides against *S. aureus* ATCC 29213 (P19-20); Figure S8. Time-kill kinetic curves of *S. aureus* ATCC 29213 after treatment with different concentrations of N_4_ (P20-21); Figure S9. The development of resistance to N_4_ (P21); Figure S10. Rg, SASA, RMSD and the hydrogen bonds number of N_4_ in 100 ns (P21-22); Figure S11. Tht fluorescence imaging of N_4_ at a concentration of 16 μM (P22-23); Figure S12. ANS fluorescence intensity changes of N_4_ in aqueous solution containing SDS (P23); Figure S13. The antibacterial mechanism of the nano-short peptides against *S. aureus* ATCC 29213 (P23-28); Figure S14. Effect of A_4_, B_4_, and N_4_ on the cytoplasmic membrane permeability of *E. coli* ATCC 25922 (P28-29); Figure S15. Effect of A_4_, B_4_, and N_4_ on ROS accumulation in *E. coli* ATCC 25922 and *S. aureus* ATCC 29213 (P29); Figure S16. RP-HPLC analysis of N_4_ after incubation with serum isolated from mice for different periods of time (P30). Table S1. Sequence and key physicochemical parameters of nano-short peptides (P31); Table S2. Calculation of the binding energy of the N_4_ intermolecular force (P31-32); Table S3. The amide I band in the FTIR spectrum of N_4_ analysis (P32).

**Supporting Text:**

Cell culture and bacterial strains

Mouse macrophages (RAW 264.7, RRID: CVCL_0493), human embryonic kidney cells (HEK 293 T, RRID: CVCL_0063) and intestinal porcine epithelial cells (IPEC-J2, RRID: CVCL_2246) were purchased in October 2022 from FanO Bio Inc.

The bacterial strains Escherichia coli (*E. coli*) ATCC25922, *E. coli* K88, *E. coli* K99, *E. coli* 078, *E. coli* UB1005 Pseudomonas aeruginosa (*P. aeruginosa*) ATCC 27853, *P. aeruginosa* PAO1, *S. typhimurium* C7731, Salmonella typhimurium (*S. typhimurium*) ATCC14028. Staphylococcus aureus (*S. aureus*) ATCC 29213, *S. aureus* ATCC 25923, *MRSA* 43300, Enterococcus faecalis (*E. faecalis*) ATCC29212, Staphylococcus epidermidis (*S. epidermidis*) ATCC12228, were obtained from the College of Veterinary Medicine, Northeast Agricultural University. Acinetobacter baumannii (*A. baumannii*) 1901, *A. baumannii* 1902, *P. aeruginosa* 25349, *S. aureus* 11011 *S. pullorum* C7913 were preserved through cryopreservation by the College of Animal Science and Technology at Northeast Agricultural Univers

**Supporting Figures:**

The HPLC spectra of all nano-short peptides are shown in Figure S1. The chromatograms demonstrate their respective retention times (RT), and the corresponding peak area percentages exceeding 95% confirm their high purity.


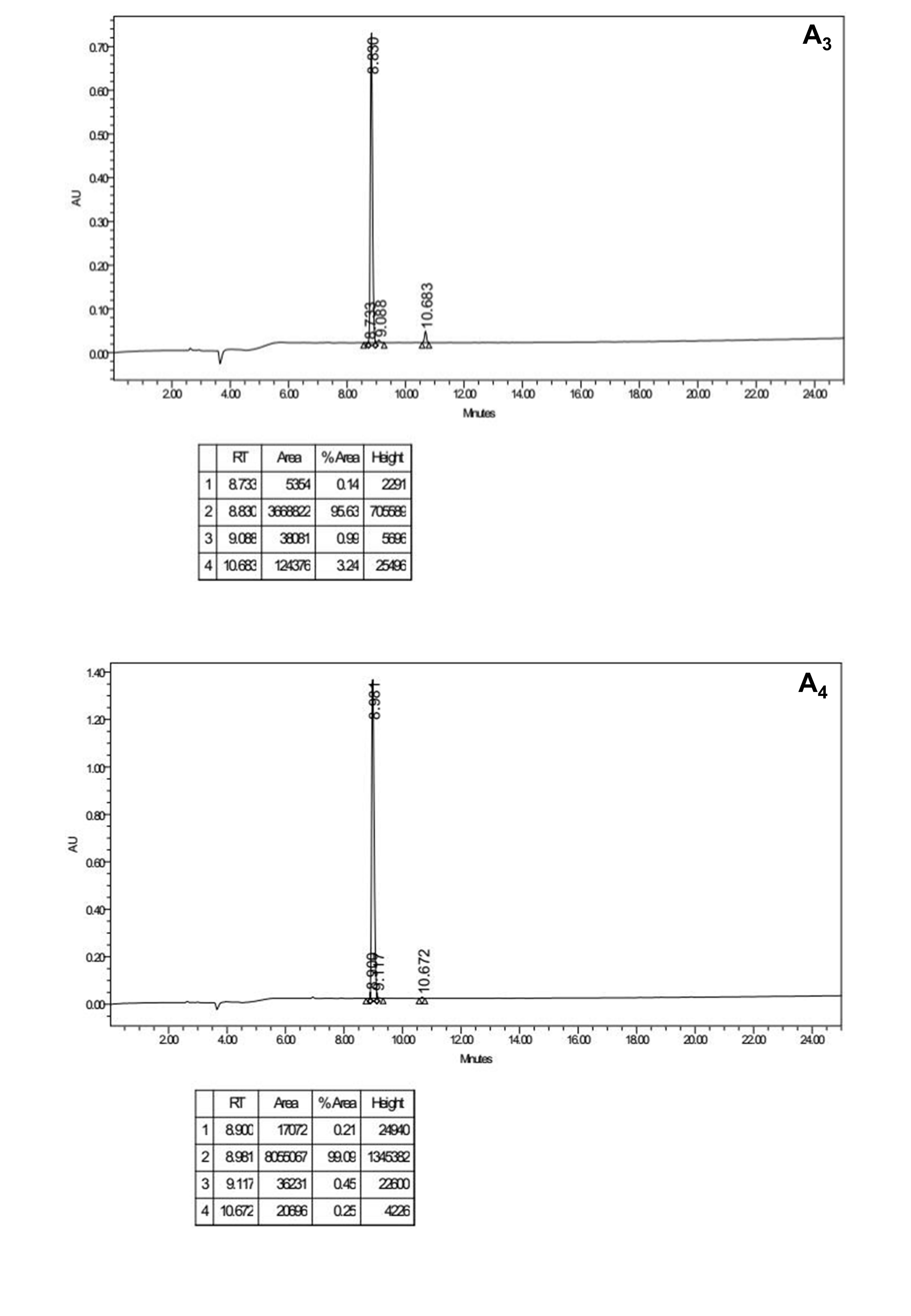


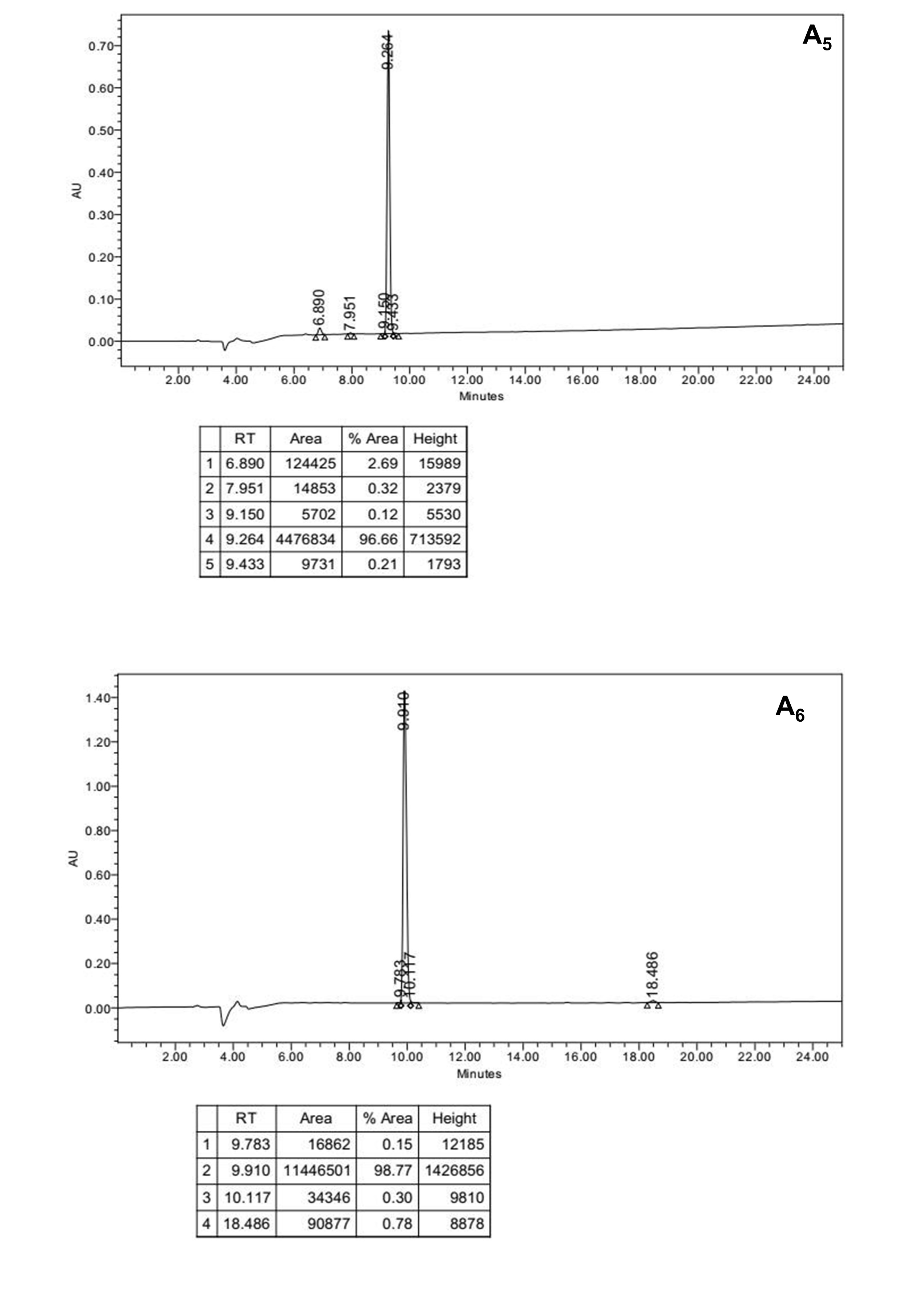


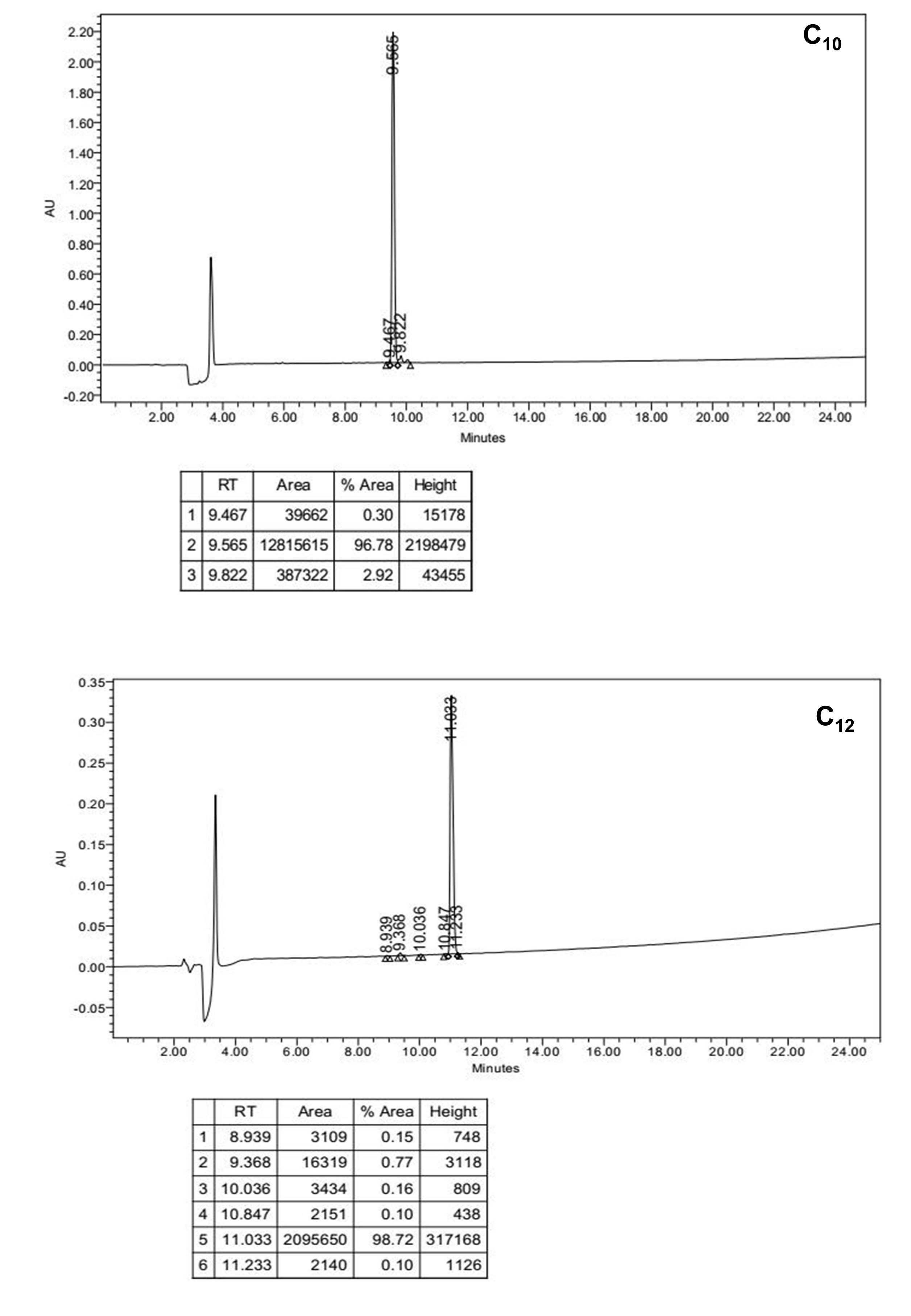


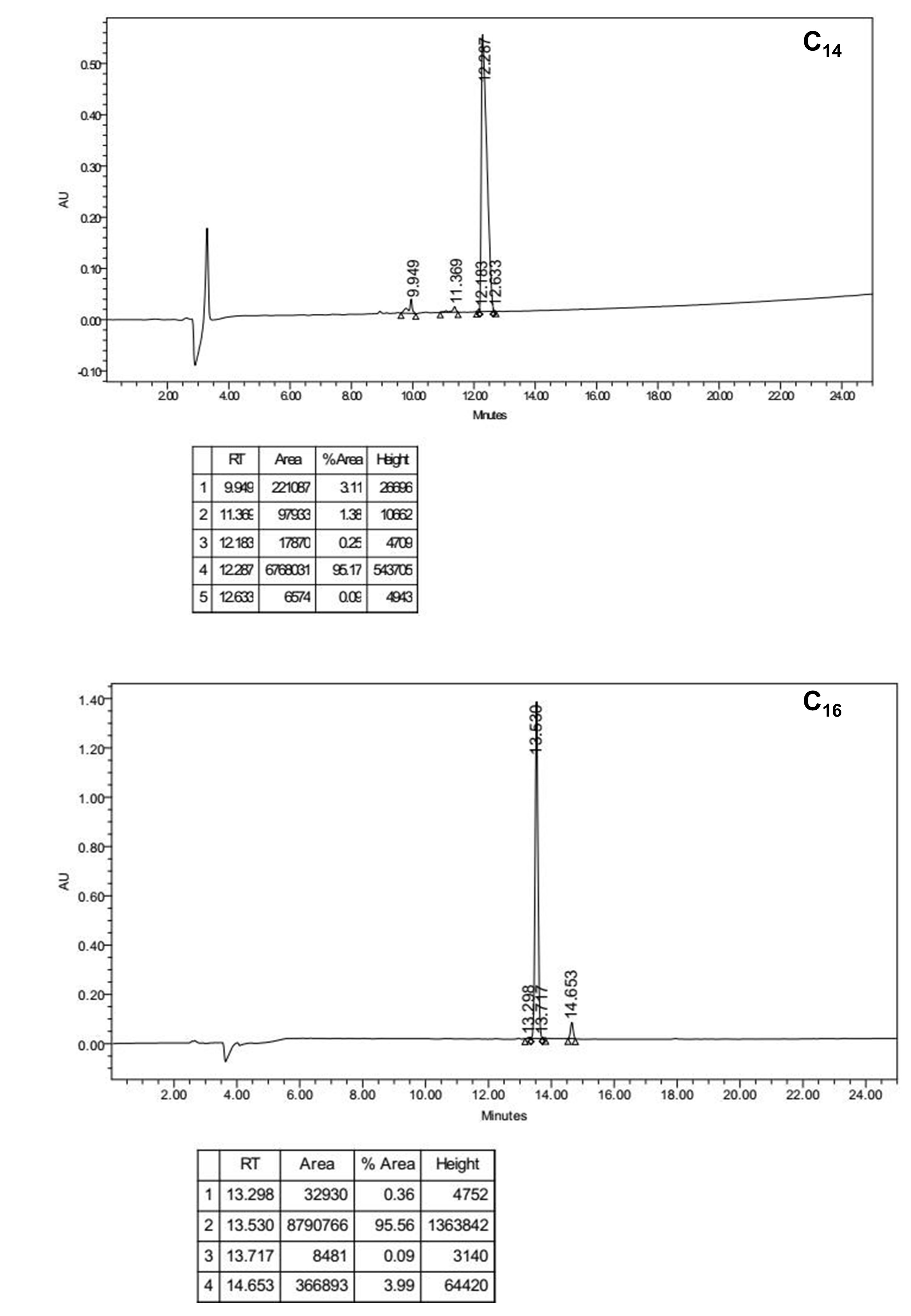


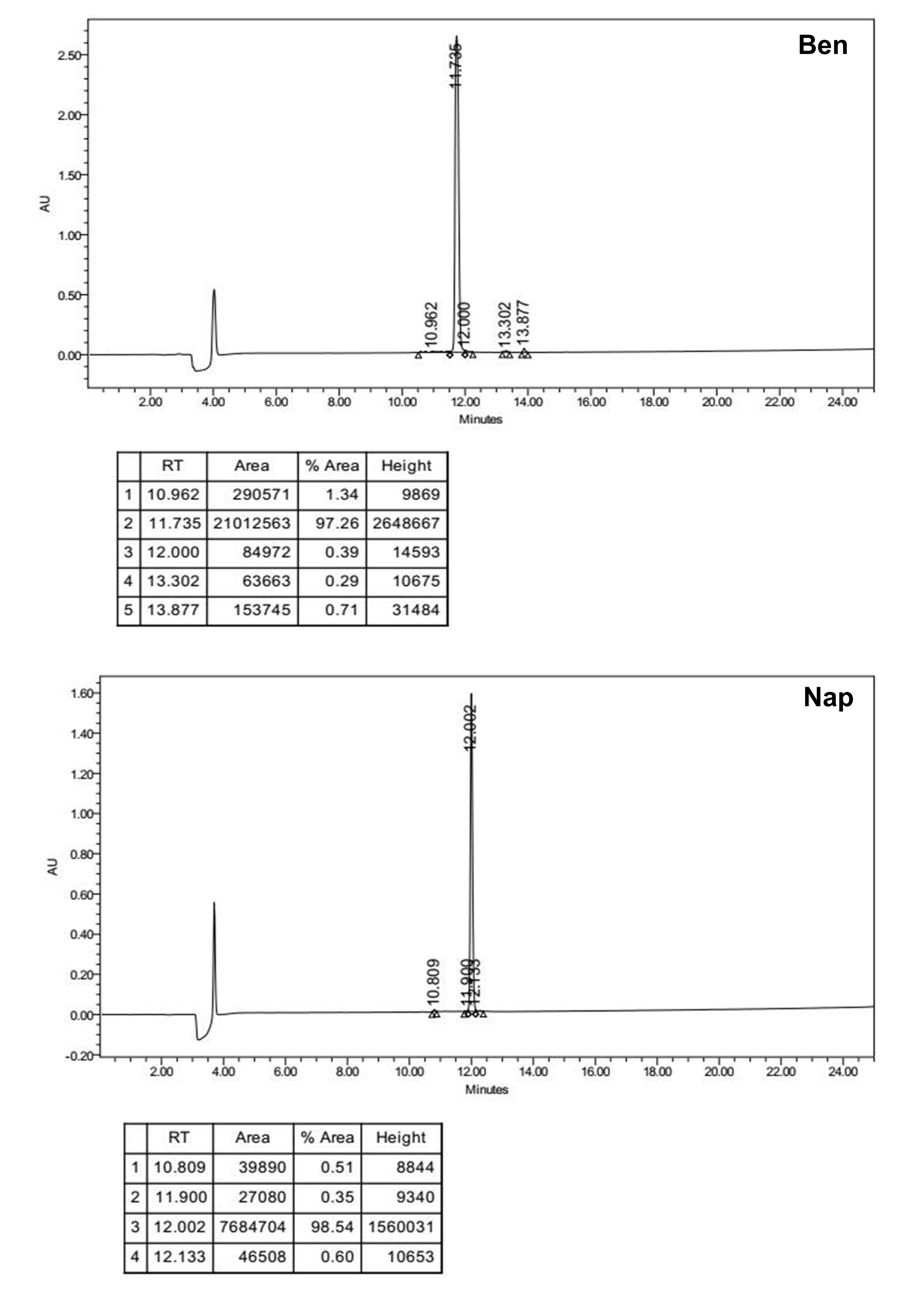


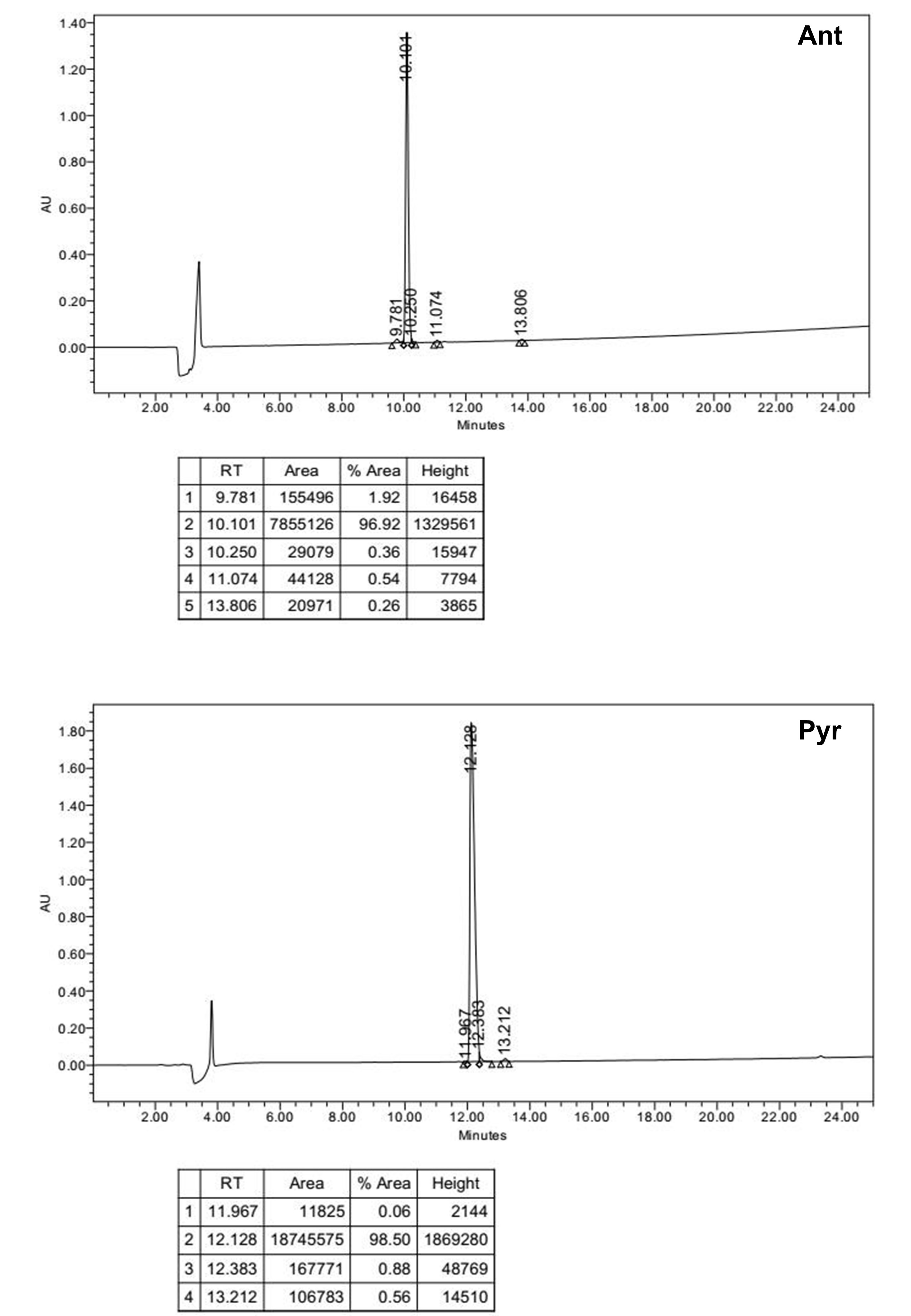


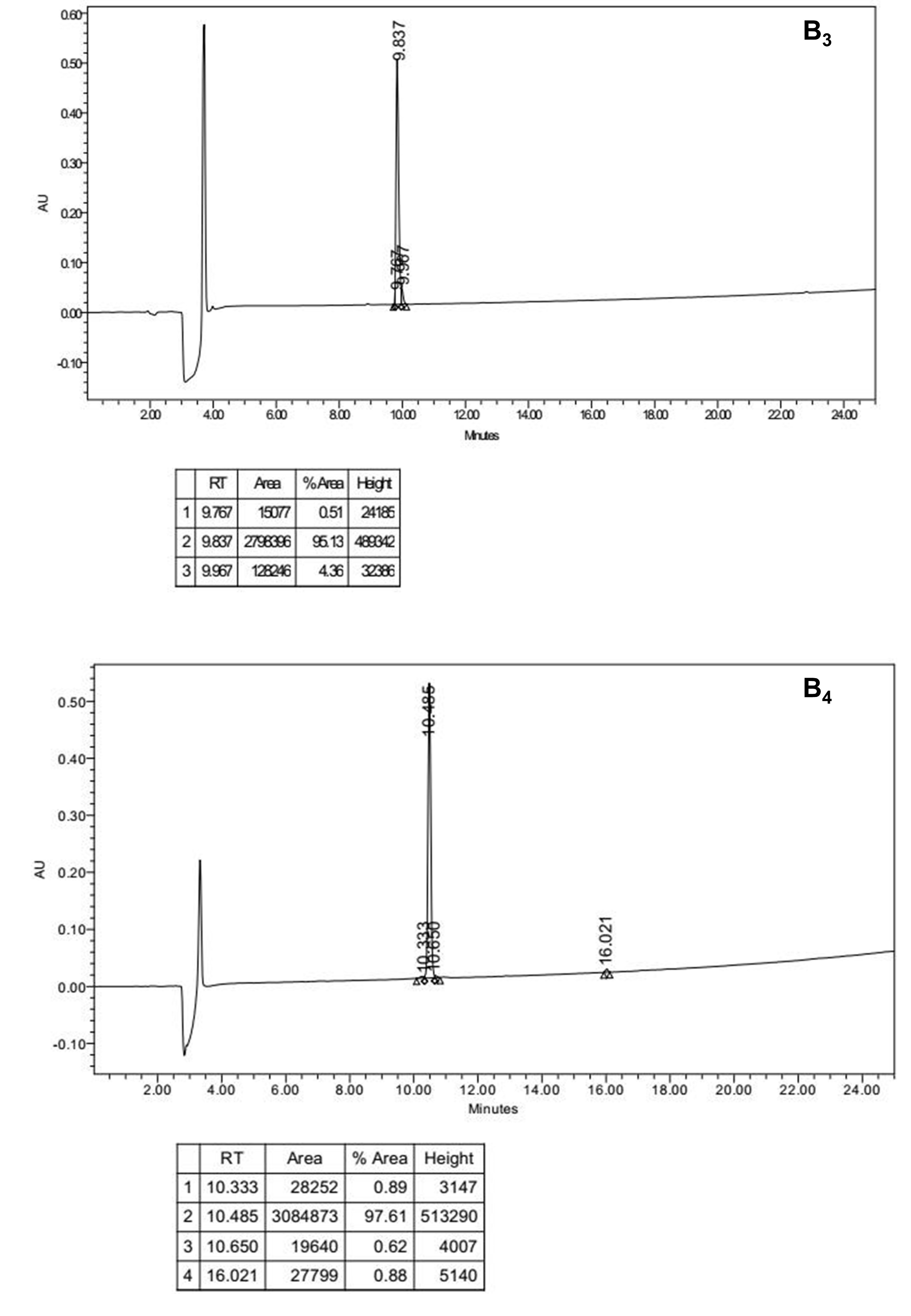


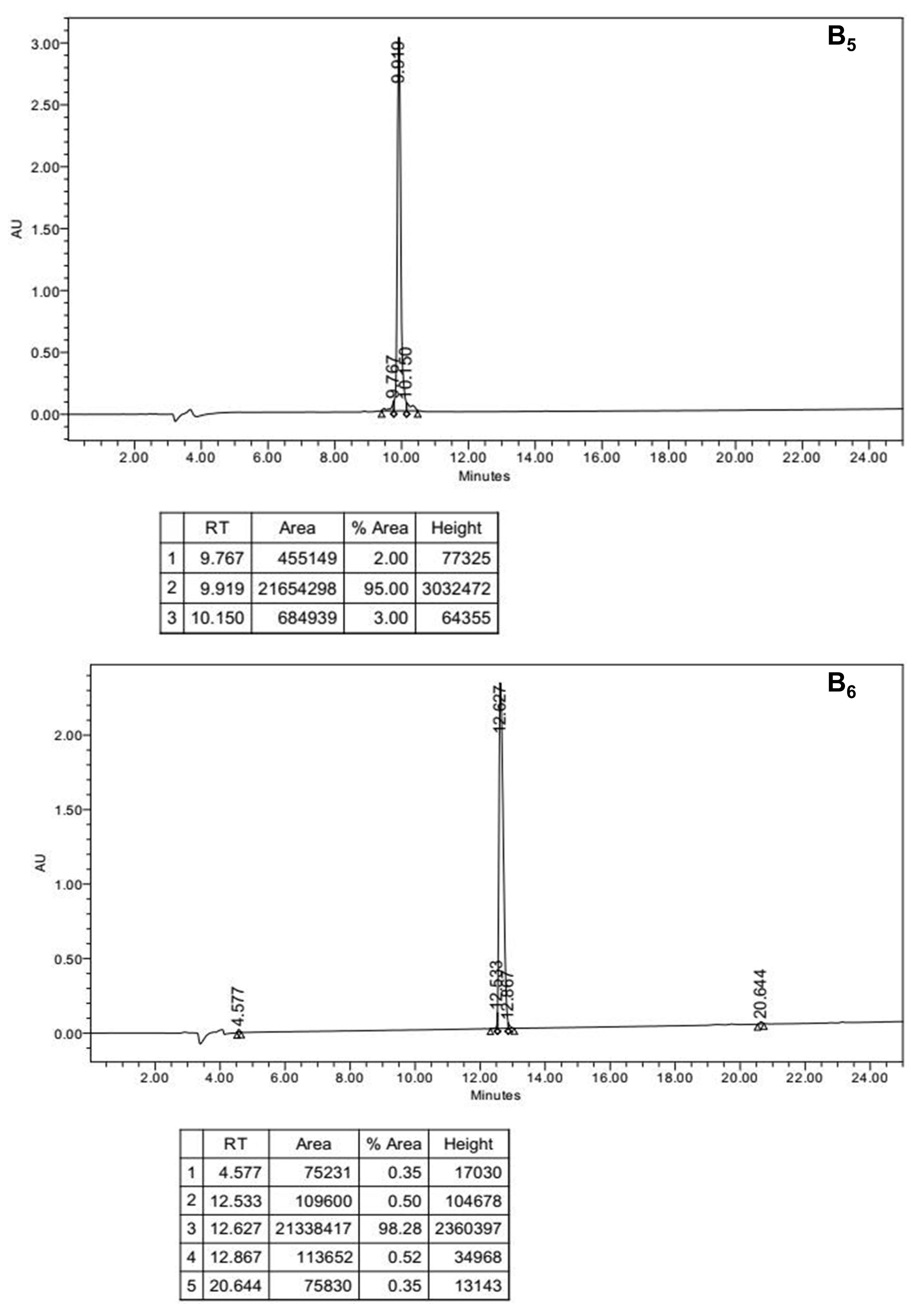


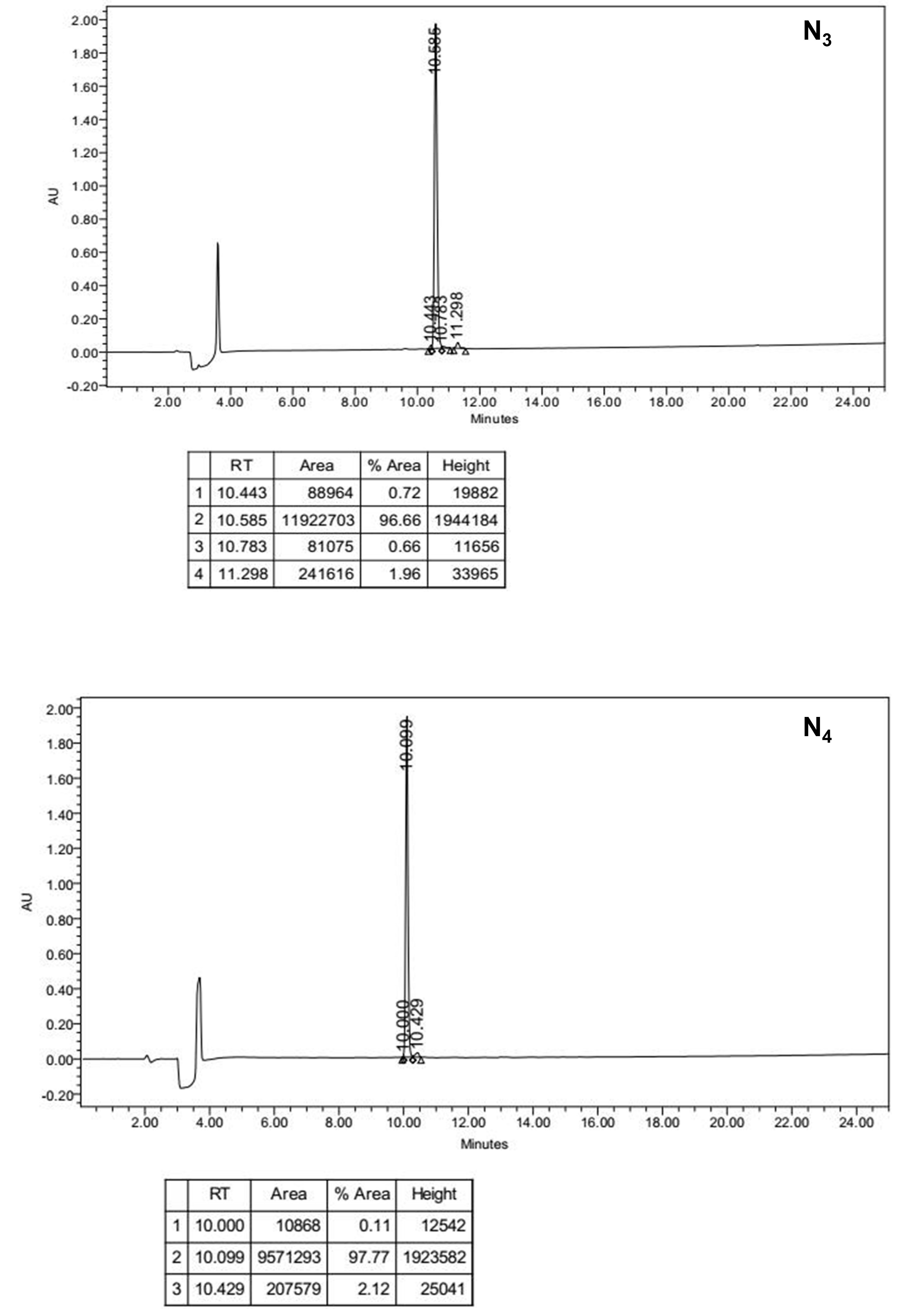


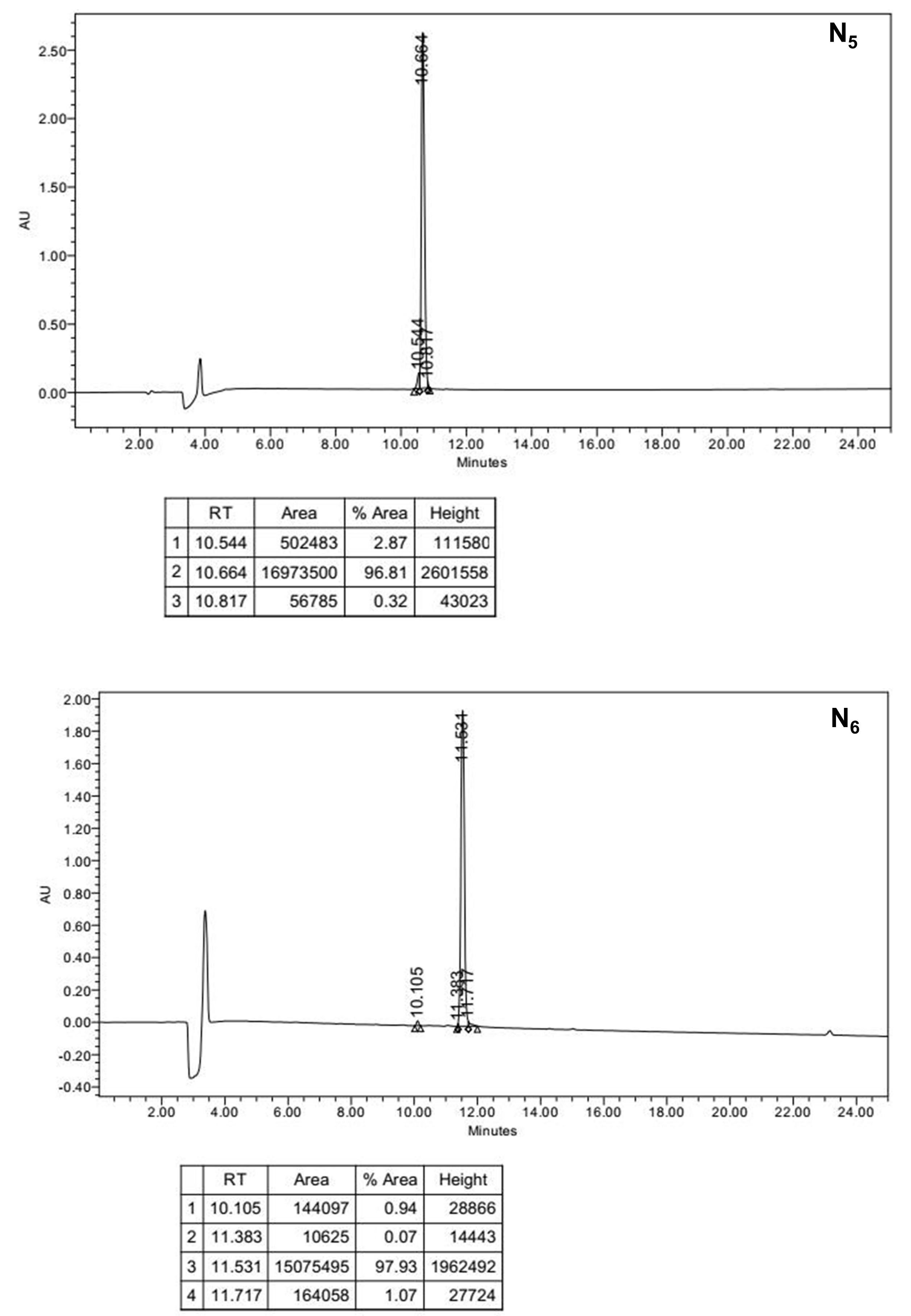


Figure S1. Reverse high performance liquid chromatography (HPLC) spectra of nano-short peptides.

The MS spectra (Figure S2) of nano-short peptides reveal their relative molecular weights. Hydrophobic modifications with different substituents confer varying molecular weights to the nano-short peptides: A_n_ series (797.34-1011.6), fatty acid substitutions (737.86-822.5), aromatic hydrophobic groups substitutions (688.26-812.26), B_n_ series (966.42-1348.74), and N_n_ series (1176.48-1768.98). Their detailed molecular weights and physicochemical parameters are shown in Table S1.


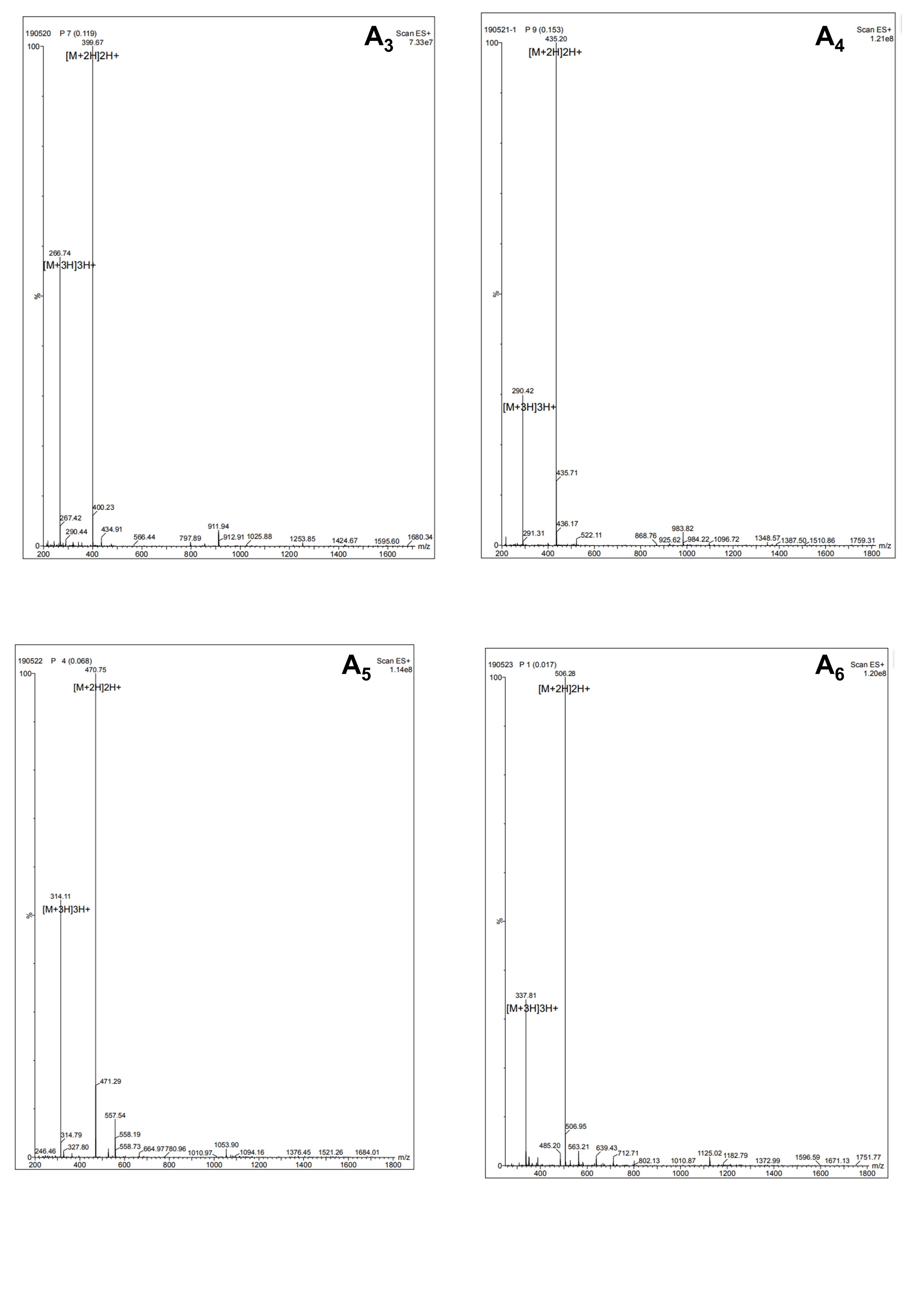

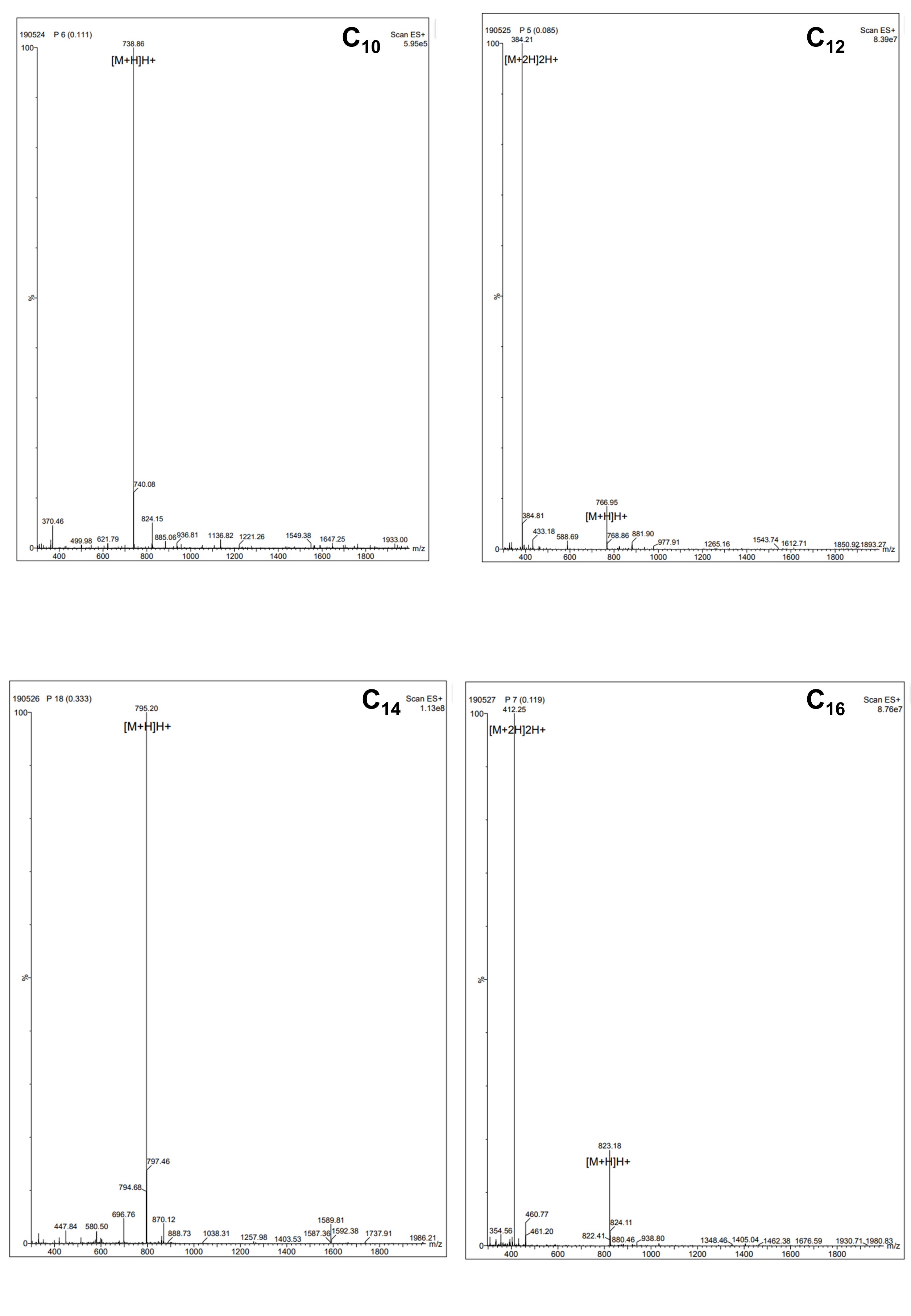


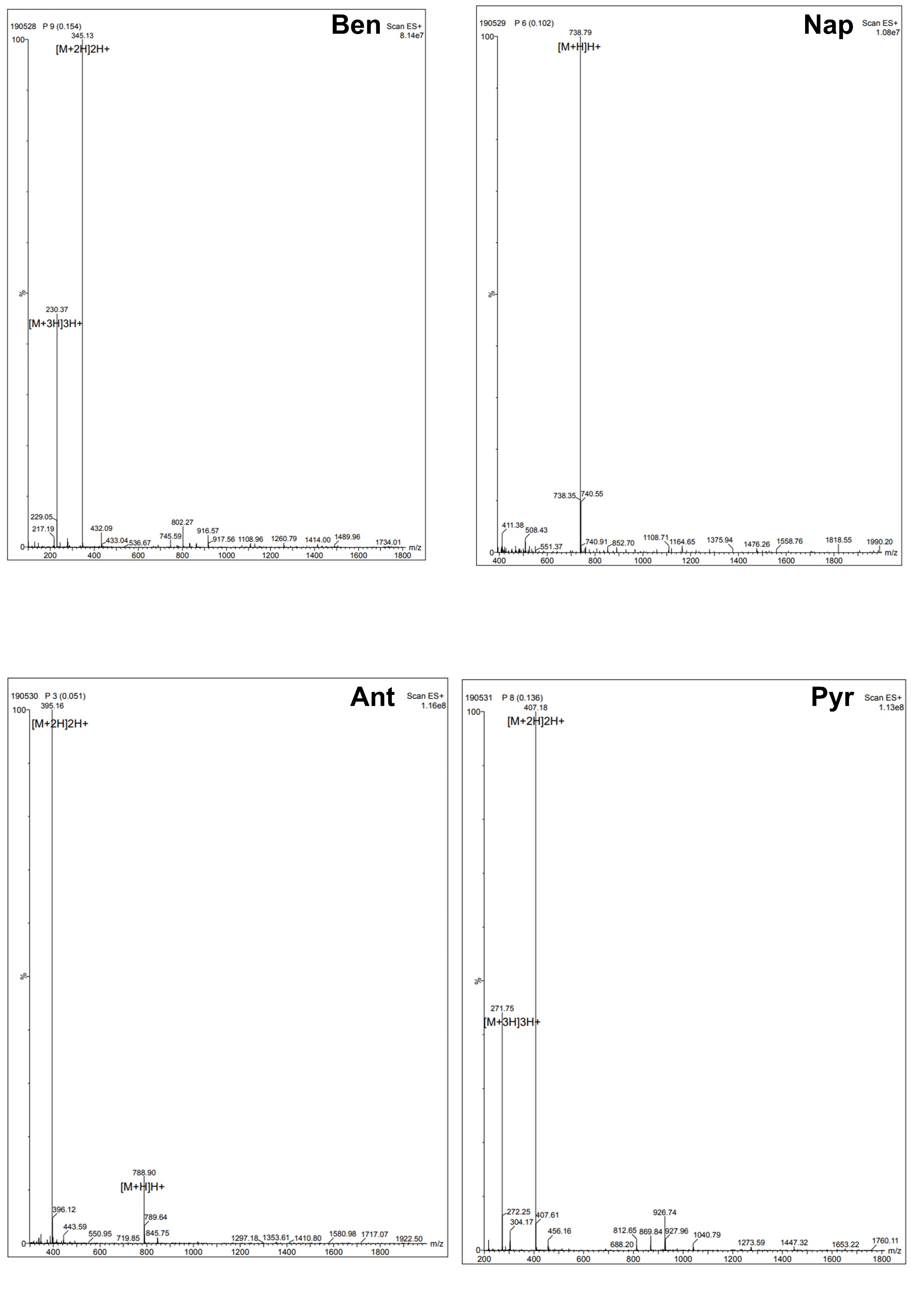


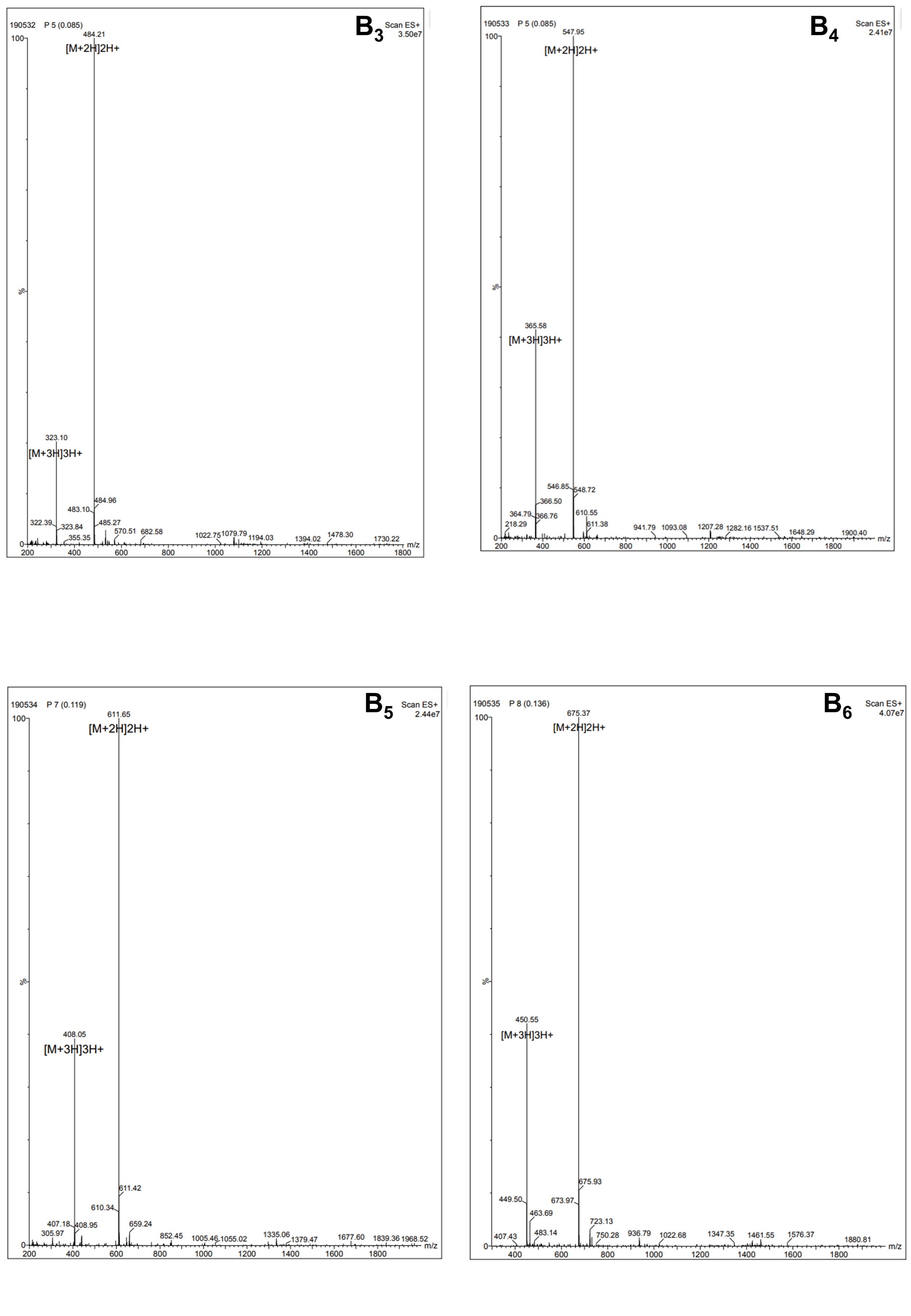


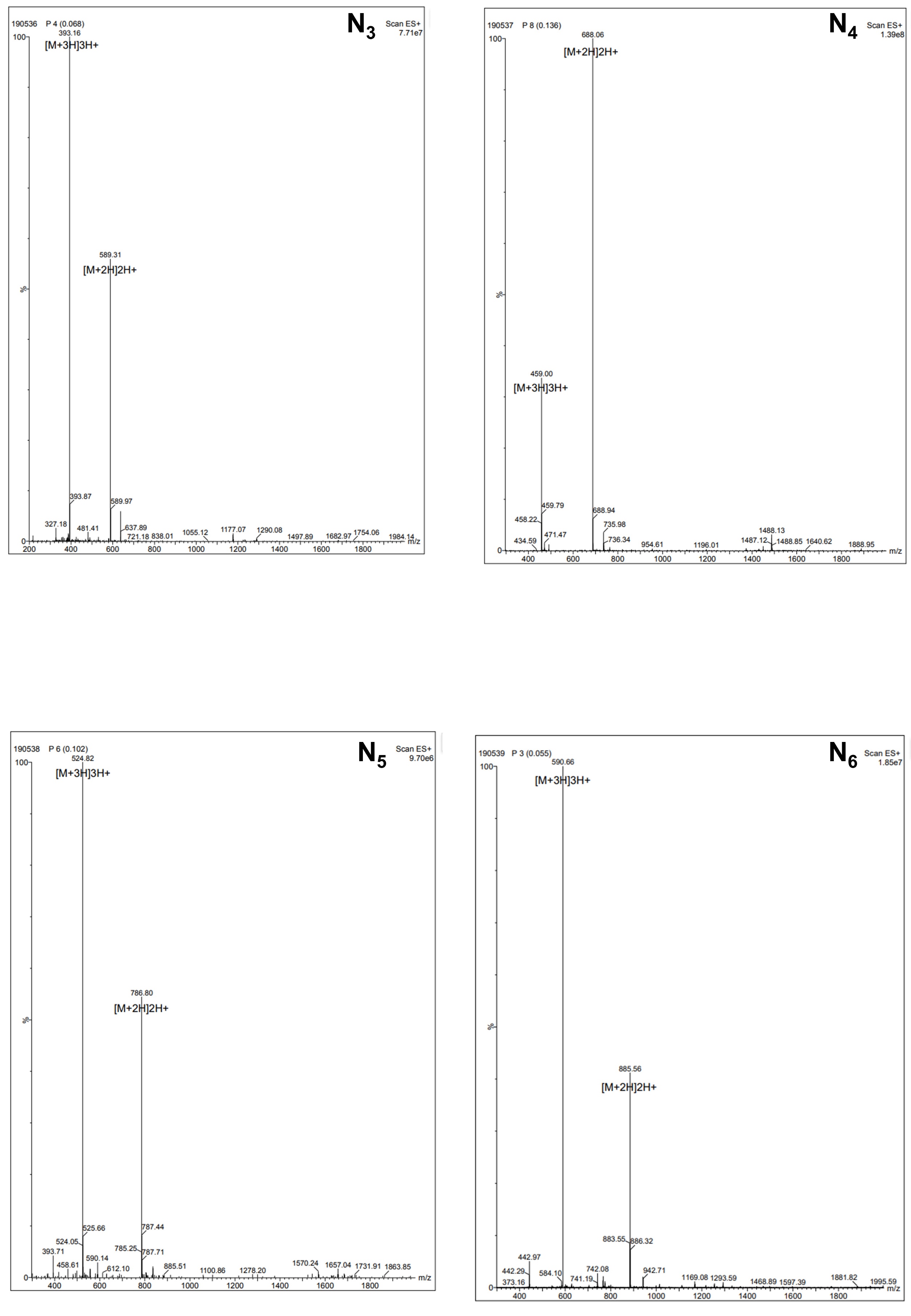


Figure S2. Mass spectrometry (MS) of nano-short peptides.

The ANS fluorescence spectra and 3D structural diagrams of the nano-short peptides are shown in Figure S3, revealing the potential relationship between the hydrophobic groups and their ability to induce self-assembly. The nano-short peptides displayed in the figure did not exhibit a concentration-dependent fluorescence surge, indicating that these hydrophobic groups did not promote nano-structural propensity.


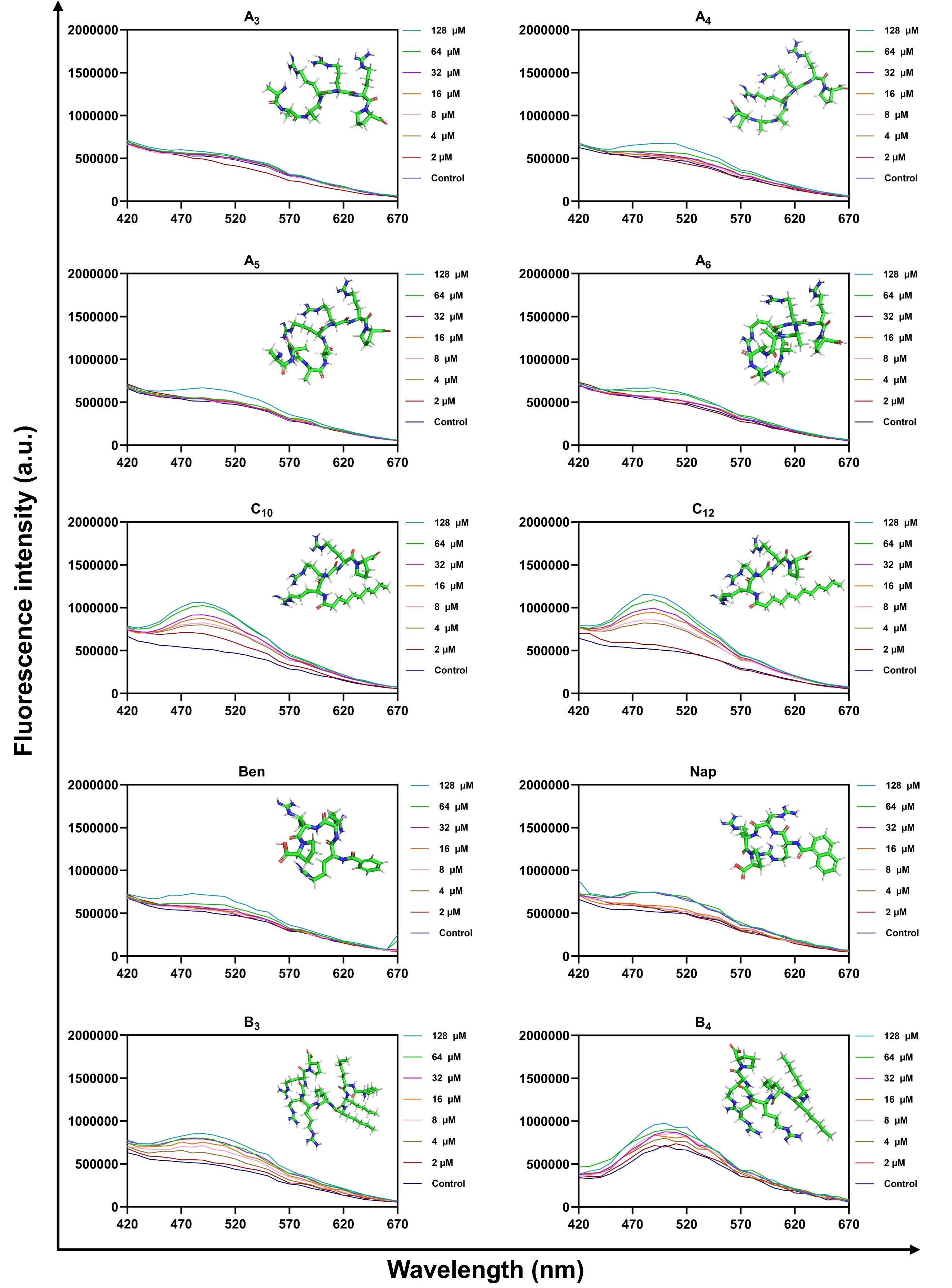


Figure S3. ANS fluorescence intensity changes and 3D structure diagram of nano-short peptides.

Furthermore, as shown in Figure S4, linear fitting of the representative fluorescence intensities indicated no detectable critical aggregation concentration (CAC) for these nano-short peptides (as presented in Figure S3), further supporting this observation.


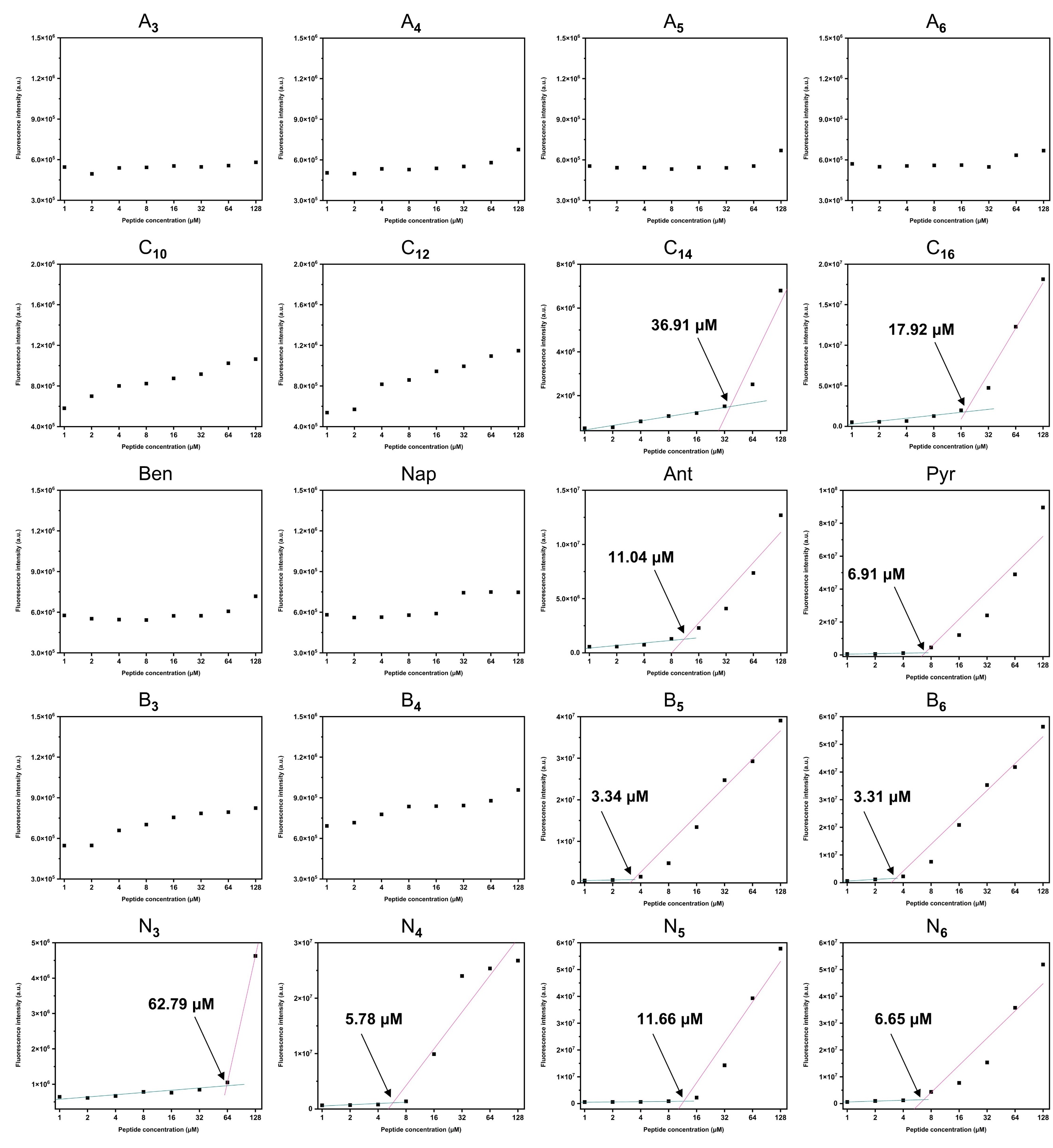


Figure S4. CAC values of nano-short peptides in aqueous solution.

The geometric mean of IC_50_ values (the lowest peptide concentration required to induce at least 50% cell death) for the nano-short peptides against three tested mammalian cell lines is shown in Figure S5A. The introduction of all hydrophobic groups did not lead to a significant increase in cytotoxicity. At the highest tested concentration (128 μM), none of the nano-short peptides induced more than 50% mortality in the three cell types, confirming their excellent biocompatibility. Figure S5B displays the HC_20_ values (the lowest peptide concentration required to induce 20% hemolysis of erythrocytes) of the nano-short peptides. The incorporation of long-chain fatty acids compromised the selectivity of the nano-short peptides, with C_16_ causing more than20% hemolysis at a concentration of 128 μM. Studies have shown that excessive hydrophobicity introduced by long-chain fatty acids can cause damage to mammalian cells, leading to a loss of selectivity in antimicrobial peptides (AMPs) ^1^. Subsequently, based on IC_50_, HC_20_, and GM_MIC_ values, we calculated the geometric mean of the selectivity index (SI), as shown in Figure S5C. The SI of the nano-short peptides improved to varying degrees after hydrophobic modification-mediated nanoengineering. Using twice the value of the template peptide (the GM_SI_ values of A_n_ series of template peptides is 1) as the baseline, C_12_ (GM_SI_ = 2.52), C_14_(GM_SI_ = 4.81), C_16_ (GM_SI_ = 2.8), B_4_ (GM_SI_ = 9.19), N_3_ (GM_SI_ = 16), and N_4_ (GM_SI_ = 50.8) were selected for subsequent studies.


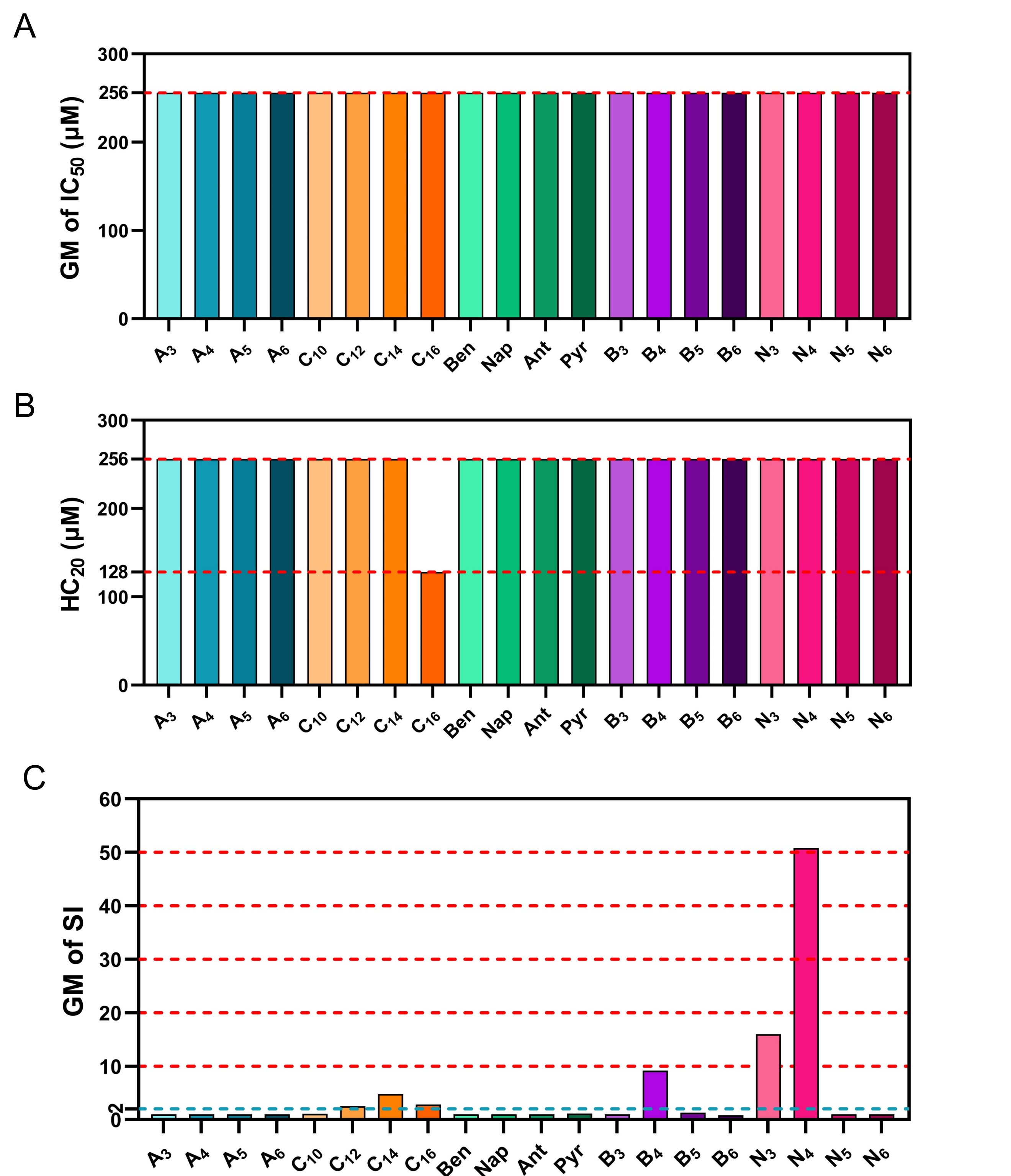


Figure S5. GM of IC_50_, HC_20_ and GM of SI values for nano-short peptides. (A)Geometric mean of IC_50_ values for nano-short peptides for different cells. (B) HC_20_ values of nano-short peptides. For HC_20_ or IC_50_calculations, if no 20% hemolysis or 50% cell death was detected at > 128 μM, 256 μM was used. (C) Geometric mean of SI for nano-short peptides. SI: selectivity index = HC_20_ or IC_50_ / GM_MIC_.

Figure S6 demonstrates the antibacterial activity of the nano-short peptides against *S. aureus* ATCC 29213 in the presence of physiological concentrations of salt ions and high concentrations of serum. Similar to the trend observed for against *E. coli* ATCC 25922, the presence of Na^+^ and Ca^2+^ reduced the antibacterial activity of the nano-short peptides, while serum caused the fatty acid-substituted C_n_ series peptides to lose activity under the tested conditions (MIC > 128 μM). As mentioned in the main text, the charge-shielding effect of Na⁺ and the competitive cationic interaction of Ca²⁺ with the peptides interfere with the ability of AMPs to recognize and bind to negatively charged components of the bacterial membrane^2, 3^. The inhibitory effect of serum on the Cₙ series peptides may be attributed to serum albumin-mediated competitive binding^4-6^. In contrast, the superior antibacterial activity of the Nₙ series peptides under simulated physiological conditions benefits from the enhanced hydrophobicity and the formation of nanostructures induced by the incorporation of naphthyl groups.


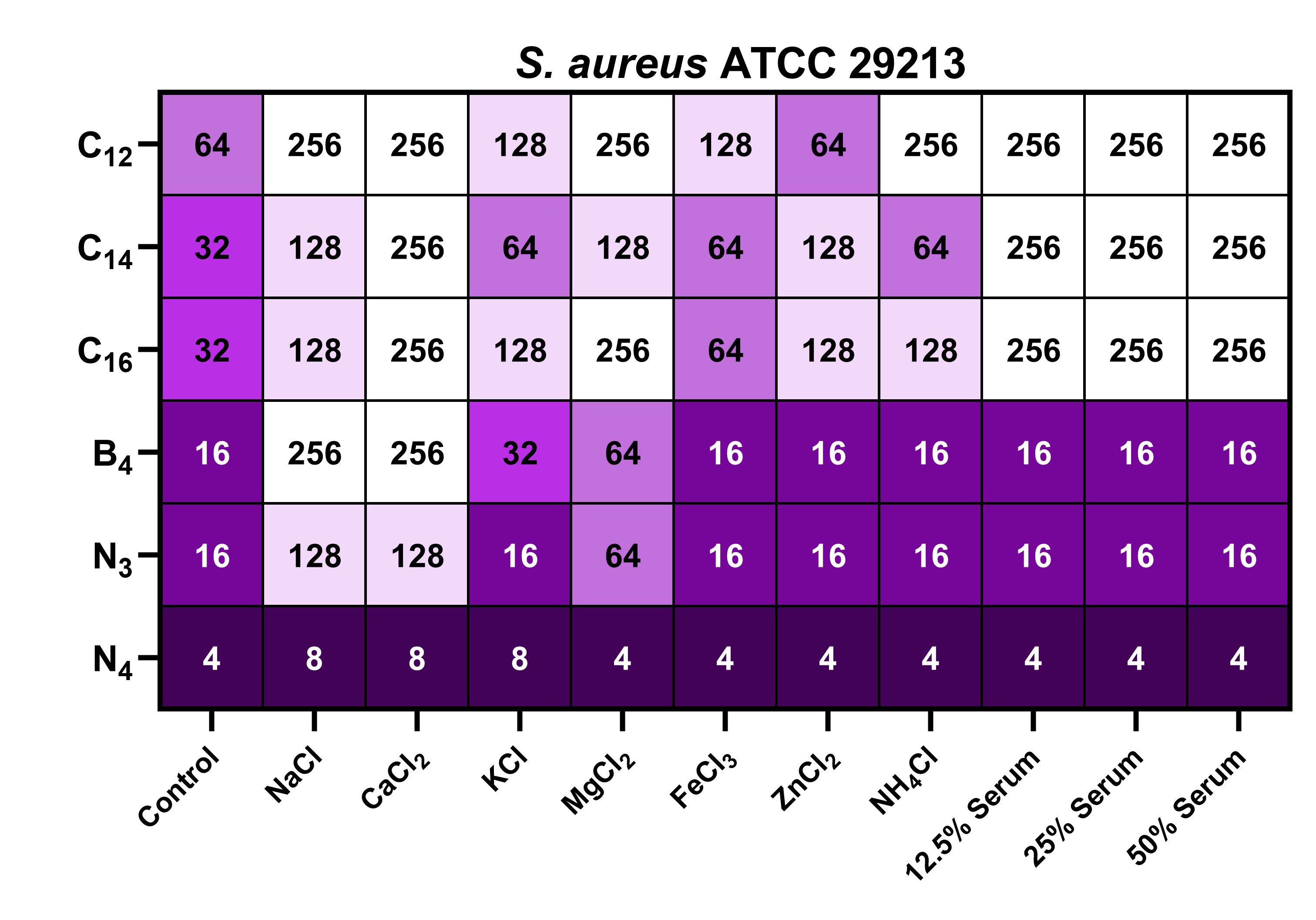


Figure S6. The serum and salt ion stability of nano-short peptides against *S. aureus* ATCC 29213. The MIC values of nano-short peptides against *S. aureus* ATCC 29213 in the presence of salt ion and serum A value of 256 indicates no detectable antimicrobial activity under the test conditions (n=3).

Figure S7 demonstrates the MIC values of nano-short peptides against *S. aureus* ATCC 29213 after incubation with different concentrations (1, 2, 4, 8 mg mL^-1^) of various proteases. In the complete replacement strategy, the replacement of alanine with fatty acids endowed the nano-short peptides with excellent protease resistance (MIC values change fold ≤ 2). In contrast, the tail-anchoring strategy, which retains natural amino acids, introduces the risk of protease recognition and degradation. Butyl-anchored B_4_ exhibited only limited resistance to low concentrations of pepsin (1 and 2 mg mL^-1^), with the MIC values change fold ≤ 2. In contrast, the naphthyl-anchored N_3_ and N_4_ exhibited exceptional protease stability. As mentioned in the main text, the enhanced stability of N_3_ and N_4_ may be attributed to two potential mechanisms. First, the bulky, cyclic structure of the naphthyl group may create a steric hindrance that blocks proteases from recognizing and cleaving the peptide^7^. Besides, the propensity of N_3_ and N_4_ to form nanostructures by self-assembly could shield protease recognition sites.


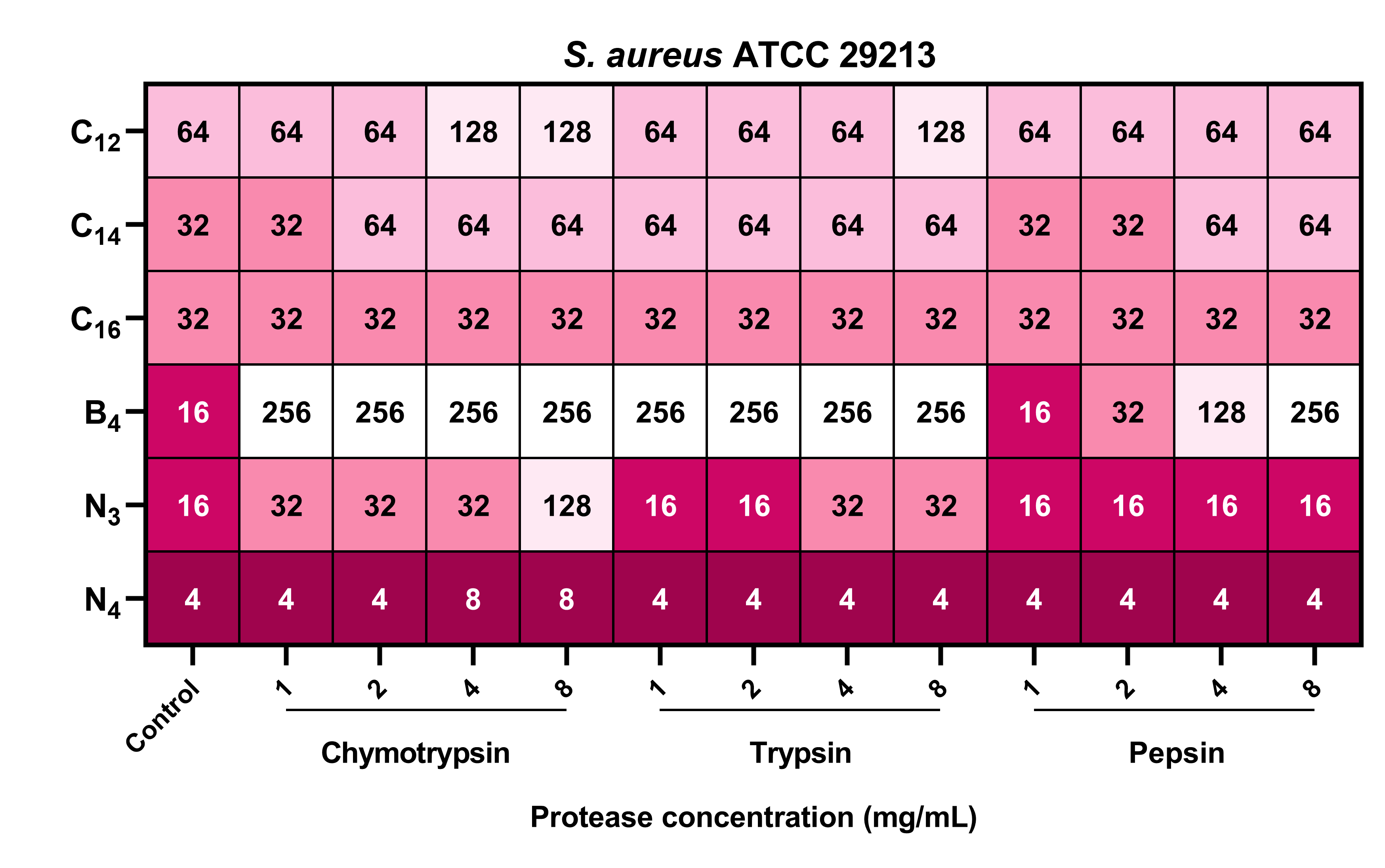


Figure S7. The protease stability of nano-short peptides against *S. aureus* ATCC 29213. The MIC values of nano-short peptides against *S. aureus* ATCC 29213 after incubation with different concentrations (1, 2, 4, 8 mg/mL) of various proteases for 1h A value of 256 indicates no detectable antimicrobial activity under the test conditions (n=3).

Time-kill kinetic curves of *S. aureus* ATCC 29213 after treatment with different concentrations of N_4_ were shown in Figure S8. N_4_ exhibited time- and dose-dependent bactericidal effects against *S. aureus* ATCC 29213. At a concentration of 1 × MIC, N4 eliminated all *S. aureus* ATCC 29213 cells within 3000 s, while at 4× MIC, it achieved complete elimination within 1200 s, demonstrating its potent time- and dose-dependent bactericidal activity.


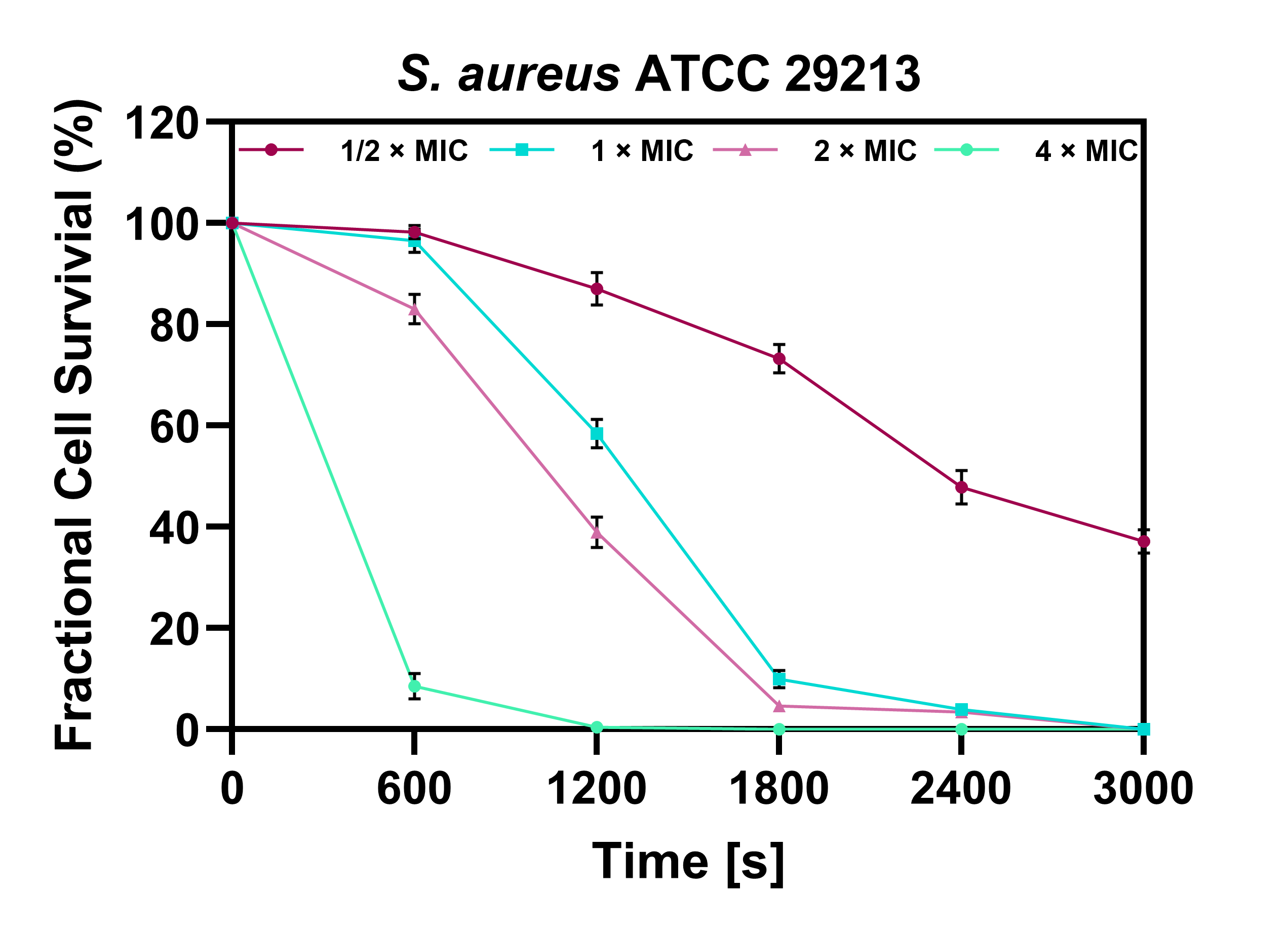
 7

Figure S8. Time-kill kinetic curves of *S. aureus* ATCC 29213 after treatment with different concentrations of N_4._ Data are the mean ± SD; n = 3.

As shown in Figure S9, after 30 successive passages at sub-MIC concentrations, the MIC values for N_4_ against *S. aureus* ATCC 29213 remained largely unchanged. In contrast, the antibiotic control, gentamicin, exhibited a 128-fold increase in MIC values by day 17, suggesting that N_4_ exhibits a lower propensity for resistance development.


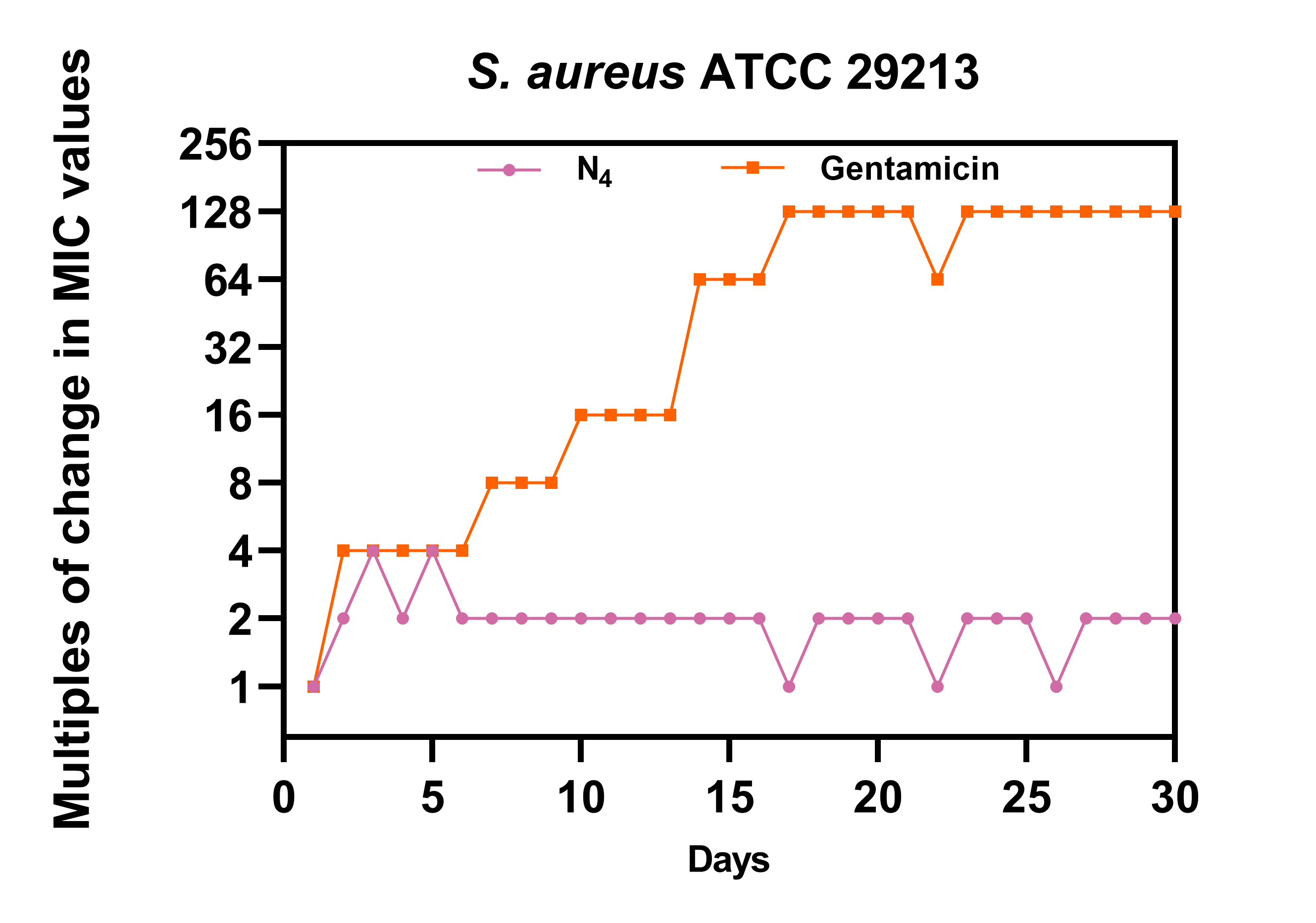


Figure S9. The development of resistance to N_4_. The drug resistance curves of *S. aureus* ATCC 29213 to N_4_ and gentamicin during a 30-day continuous induction period.

As shown in Figure S10, the radius of gyration (RG) and solvent-accessible surface area (SASA) values gradually decreased and stabilized during the simulation, while the number of hydrogen bonds steadily increased, indicating a progressively tighter self-assembled structure^8^. The eventual stabilization of the root mean square deviation (RMSD) further confirmed low structural fluctuations in the self-assembled system^9^. These results collectively demonstrate that N_4_ rapidly assembles into a stable multimeric structure.

_
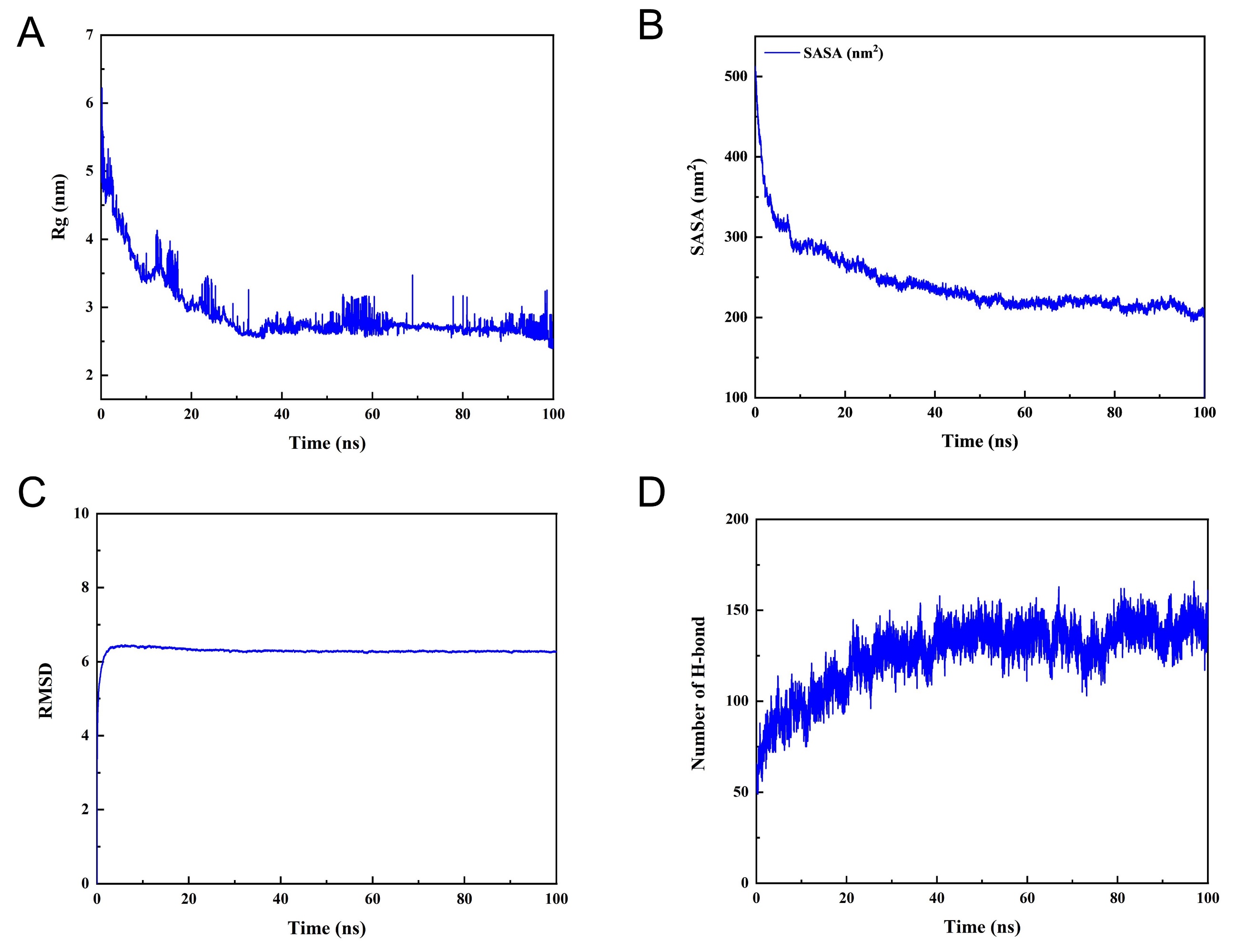
_

Figure S10. Rg, SASA, RMSD and the hydrogen bonds number of N_4_ in 100 ns. (A) The radius of gyration (Rg) of N_4_ in 100 ns was calculated using GROMACS. (B) The solvent-accessible surface area (SASA) of N_4_ in 100 ns was calculated using GROMACS. (C) The root mean square deviation (RMSD) of N_4_ in 100 ns was calculated using GROMACS. (D) The hydrogen bonds number of N_4_ in 100 ns was calculated using GROMACS.

Thioflavin T (ThT) can interact with structures exhibiting amyloid-like fibrillar characteristics^10^. As shown in Figure S11, compared to the control group, 16 μM N_4_ treated with ThT displayed clearly visible amyloid fibril structures under microscopy, providing evidence for the self-assembly of N_4_.


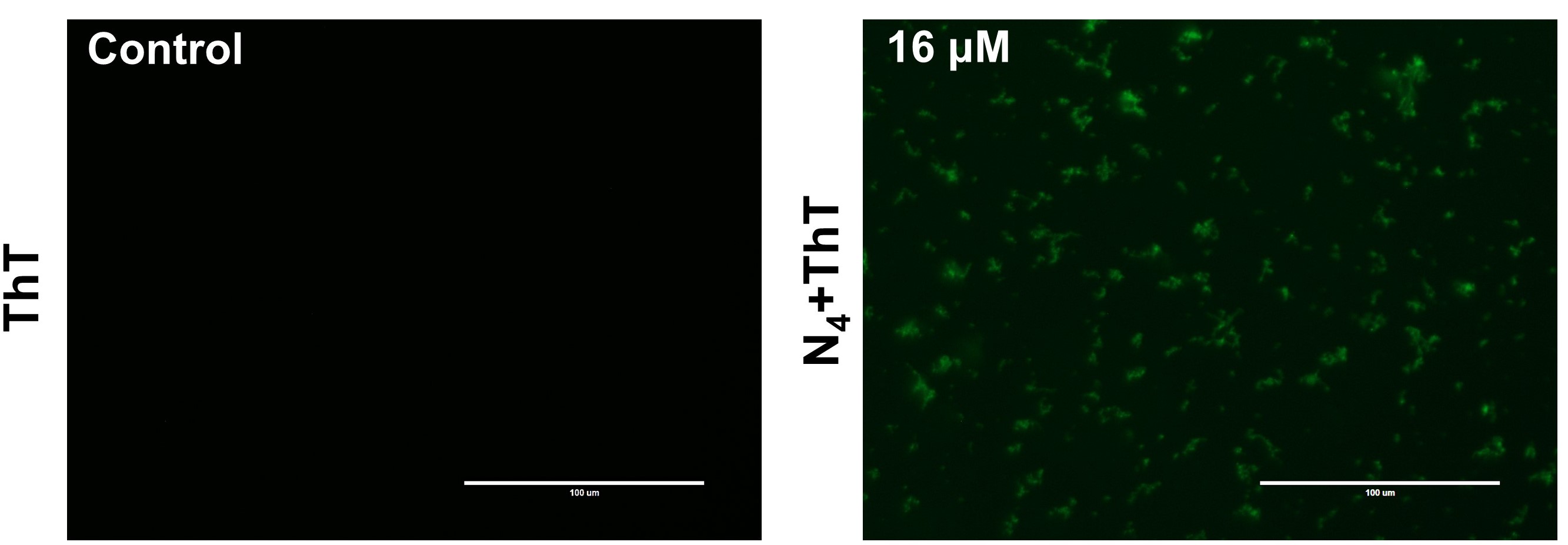


Figure S11. Tht fluorescence imaging of N_4_ at a concentration of 16 μM. Scale bars, 100 μm.

To verify the critical role of hydrophobic interactions in N_4_ self-assembly, we characterized its fluorescence changes in SDS-containing aqueous solutions, where SDS serves as a well-established hydrophobic disruptor^11^. As shown in Figure S12, N_4_ exhibited no concentration-dependent fluorescence surge in its emission spectra, indicating the absence of molecular aggregation behavior in solution. The result confirms that SDS disrupted hydrophobic interactions, thereby abolishing N4's self-assembly capability.


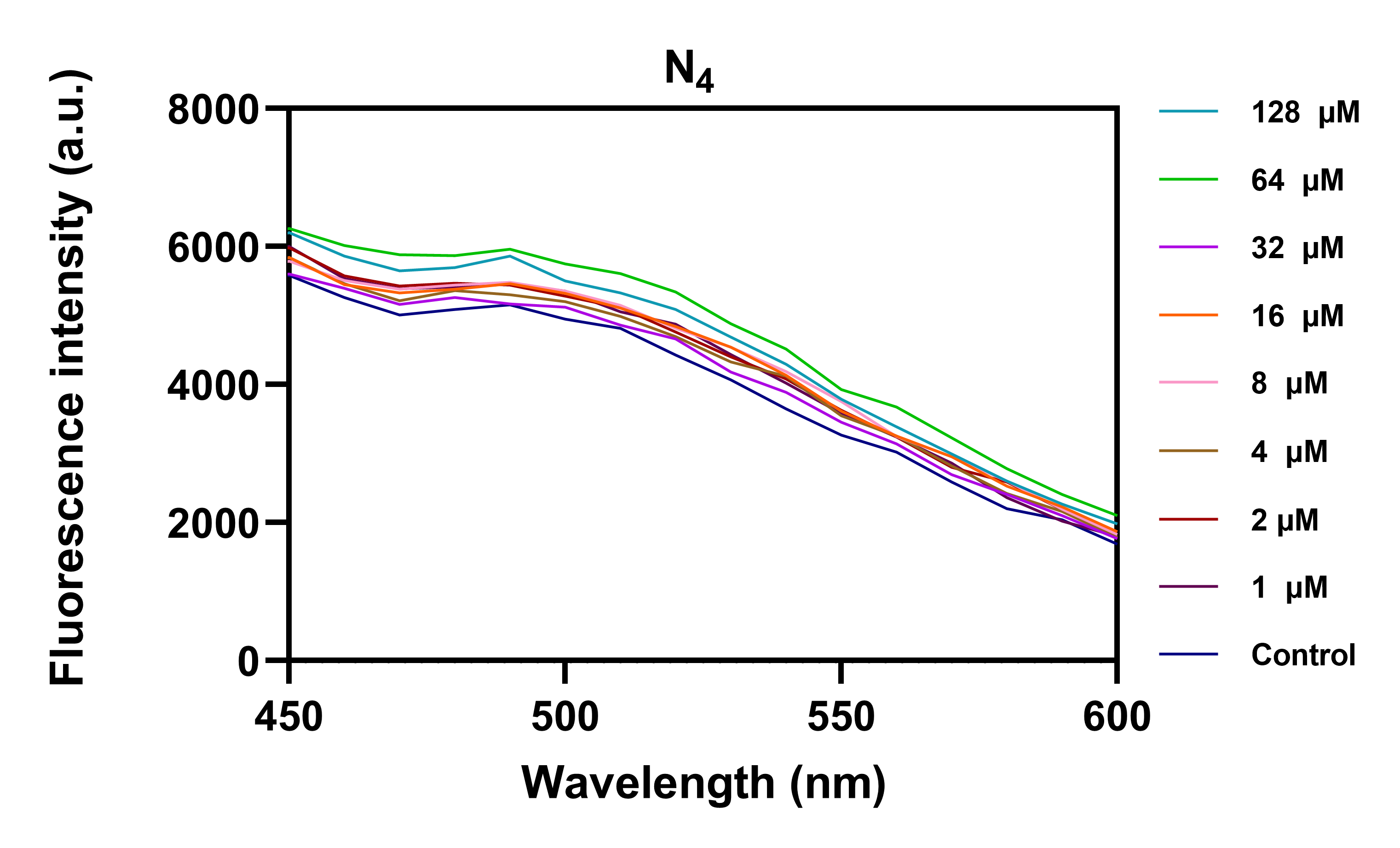


Figure S12. ANS fluorescence intensity changes of N_4_ in aqueous solution containing SDS.

Figure S13 illustrates the investigation into the antibacterial mechanism of the nano-short peptides against *S. aureus* ATCC 29213. As mentioned in the main text, consistent with established mechanisms of cationic amphiphilic antimicrobials, the initial bacterial membrane targeting of nano-short peptides may be achieved through electrostatic interactions with negatively charged ionic components, such as LPS or LTA, on the bacterial surface^12^. We first examined the affinity of the nano-short peptides for LTA in *vitro*. As shown in Figure S13A, B_4_ demonstrated enhanced LTA-binding affinity relative to N_4_, with its binding capacity attaining 85.77% and 96.79% of the antibiotic control at concentrations of 8 μM and 16 μM, respectively, while the corresponding values for N_4_ were 65.60% and 73.08%. The presence of nonpolar hydrophobic glycolipid chains in LTA suggested that hydrophobic interaction may be a potential mode of binding to LTA in addition to electrostatic attraction^13^. Thus, the anchoring of aliphatic butyl could have increased the lipophilicity of B_4_, thereby enhancing its ability to bind LTA through hydrophobic interactions. Subsequently, we investigated the permeabilizing effect of the nano-short peptides on the cell wall of *S. aureus* ATCC 29213 using the hydrophobic fluorescent probe N-phenyl-1-naphthylamine (NPN). As shown in Figure S13B, compared to the control antibiotic ciprofloxacin, B_4_ induced permeabilization rates of 19.49% and 37.80% at concentrations of 8 μM and 16 μM, respectively. In comparison, N_4_ exhibited significantly higher permeabilization, reaching 48.48% and 110.54% at the same concentrations. The superior disruptive efficacy of N_4_ over B_4_ may be attributed to the larger molecular size of the naphthyl group within the N_4_ sequence and the enhanced local concentration effects facilitated by its self-assembly properties.

Based on these demonstrated effects of the nano-short peptides on disrupting cell wall at 8 and 16 μM, we further explored their underlying membrane-perturbing mechanisms. As shown in Figure S13C, both B_4_ and N_4_ induced a time-dependent increase in propidium iodide (PI) fluorescence intensity in *S. aureus* ATCC 29213 within 1000 s, indicating gradual disruption of the bacterial membrane structure that allowed PI to penetrate and stain intracellular DNA^14^. Furthermore, compared with 8 μM, a more pronounced fluorescence enhancement was induced at 16 μM, thereby corroborating their dose-dependent characteristics. Subsequently, the fluorescent probe DiSC3-5 was used to evaluate the effect of the peptides on the cytoplasmic membrane (CM) potential of bacteria. As shown in Figure S13D, both B_4_ and N_4_ significantly increased the relative fluorescence intensity of DiSC_3_-5 in *S. aureus* ATCC 29213 within 1800 s. Consistent with the membrane integrity results, both peptides exhibited dose-dependent effects, and N_4_ exhibited a stronger depolarizing effect at equivalent concentrations. This finding underscored the ability of B_4_ and N_4_ to alter potential and disrupt ionic homeostasis by inducing CM depolarization^15^.

Inspired by the remarkable membrane-disrupting effects of the nano-short peptides, we further investigated their impact on bacterial respiration, as the respiratory chain is embedded within the cell membrane. The red tetrazolium (RT) dye can be reduced to a red-colored product by respiratory chain dehydrogenases (RCD)^16^, and was therefore used to evaluate RCD activity of *S. aureus* ATCC 29213cells treated with nano-short peptides. As observed in Figure S13E, treatment with B_4_ and N_4_ induced a significant reduction in the absorbance at 420 nm in *S. aureus* ATCC 29213. Specifically, at a concentration of 16 μM, B_4_ reduced the absorbance to 34.12%, while N_4_ decreased it to 18.84%. These results suggest that the nano-short peptides interfere with bacterial respiration by impairing RCD activity following their action on the cell membrane, potentially leading to disruptions in energy metabolism. Given the observed inhibition of bacterial respiration, we further examined changes in intracellular ATP levels, as ATP is a critical product of the respiratory process. As shown in Figure S13F, both B_4_ and N_4_ significantly reduced ATP production in *S. aureus* ATCC 29213. At a concentration of 16 μM, B_4_ decreased the ATP level to 71.18%, while N4 reduced it to 10.8%.

The above results confirm the potent bactericidal effect of N_4_ at a concentration of 16 μM. We further visualized its direct impact on bacteria at this concentration using microscopic imaging techniques. Negative TEM staining of *S. aureus* ATCC 29213 treated with N_4_ at a concentration of 16 μM (Figure S16G) revealed that N_4_ adhered to the cell surface in the form of fibrous aggregates, confirming a preliminary mechanism of membrane interaction. As shown in Figure S16H, scanning electron microscopy (SEM) analysis indicated that the *S. aureus* ATCC 29213 cell in control group exhibited a smooth membrane surface, whereas treatment with 16 μM N_4_ induced irregular vesicular protrusions and leakage of cellular contents. As demonstrated in Figure S16I, transmission electron microscopy (TEM) imaging further confirmed that untreated control cells maintained a dense internal structure with intact cellular contents. In contrast, exposure to 16 μM N_4_ led to compromised membrane integrity, leakage of intracellular material, and the formation of hollow cavities in *S. aureus* ATCC 29213. The results of the live/dead staining assay are presented in Figures S16J. Under fluorescence microscopy, the presence of chartreuse fluorescence indicated merging of the red signal from PI with the green nucleic acid stain SYTO 9, further confirming membrane damage induced by N_4_. Quantitative fluorescence analysis using ImageJ determined the red (PI) / green (SYTO 9) fluorescence ratio at various N_4_ concentrations to quantify bacterial mortality rates. Specifically, treatment with 16 μM N_4_ resulted in fluorescence overlap rates of 90.74% for *S. aureus* ATCC 29213, while at 256 μM, the overlap rates increased to 98.65%.

Additionally, treatment with 256 μM N_4_ induced agglutination of *S. aureus* ATCC 29213. Peptide-based nanostructures have been demonstrated to achieve effective bacterial eradication through agglutination^8, 17, 18^. As shown in Figure S16K, in contrast to the homogeneous bacterial suspensions observed in both the control and 16 μM N4-treated groups, exposure to 256 μM N4 resulted in rapid sedimentation of *S. aureus* ATCC 29213 within 1 h, yielding visibly clarified supernatants. Moreover, the reduction in bacterial load in the supernatant confirmed that the bactericidal efficacy was enhanced at elevated N_4_ concentrations via aggregation-mediated mechanisms (Figure S16L). As shown in Figure S16M, flow cytometry analysis demonstrated the strong membrane-disrupting activity of N_4_: treatment with 16 μM increased the proportion of PI-positive *S. aureus* ATCC 29213 cells from 1.23% to 36.7%.


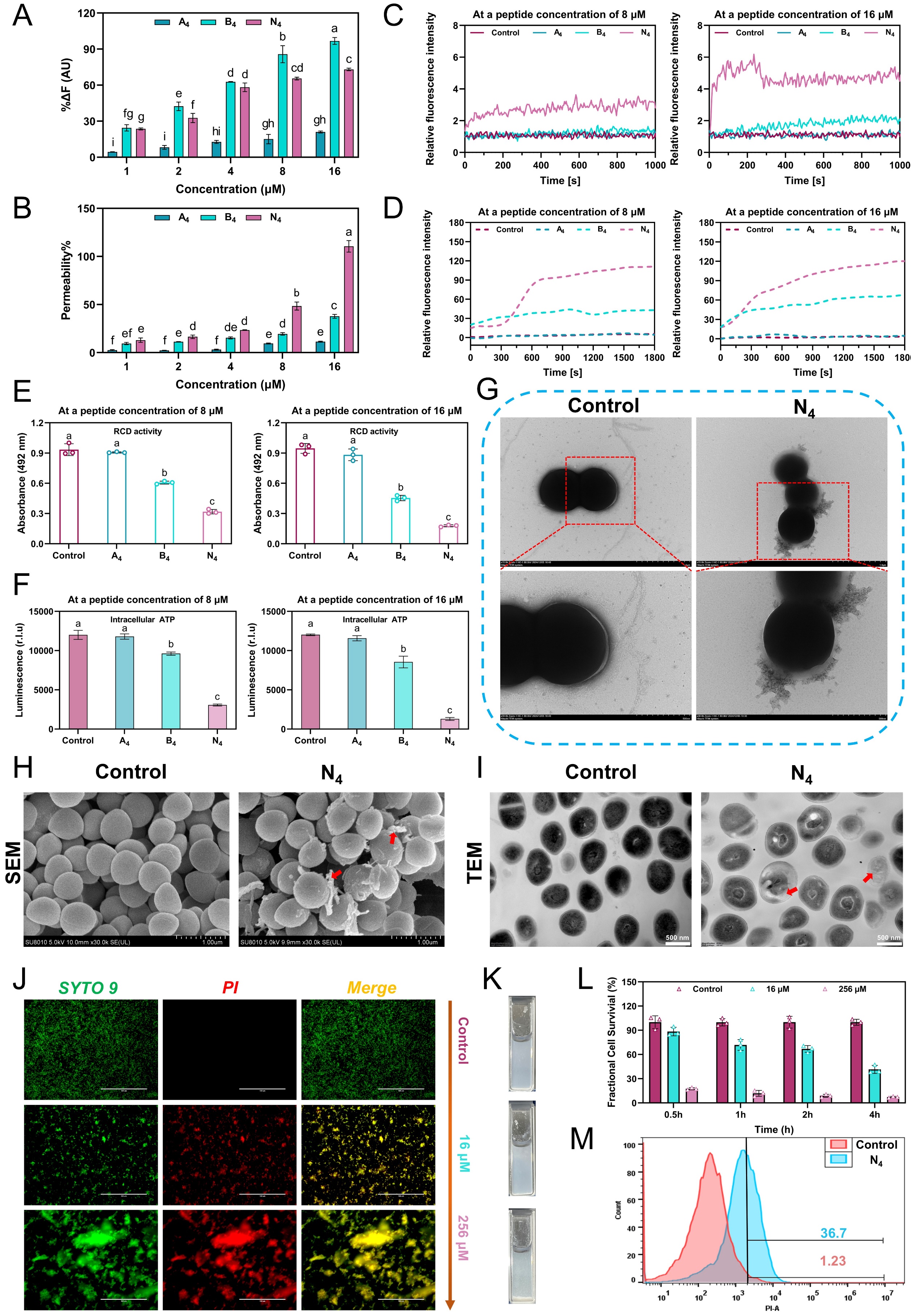


Figure S13. The antibacterial mechanism of the nano-short peptides against *S. aureus* ATCC 29213. (A) LTA binding affinities of A_4_, B_4_, and N_4_. (B) Effect of A_4_, B_4_, and N_4_ on the cell wall permeability of *S. aureus* ATCC 29213. (C) Effect of A_4_, B_4_, and N_4_ on the cell membrane integrity of *S. aureus* ATCC 29213 at different concentrations. (D) Depolarization ability of A_4_, B_4_, and N_4_ on cytoplasmic membrane of *S. aureus* ATCC 29213 at different concentrations. (E) Inhibition on respiratory chain dehydrogenase activity of *S. aureus* ATCC 29213 by A_4_, B_4_, and N_4_ at different concentrations. (F) Intracellular ATP content in *S. aureus* ATCC 29213 cells after treatment with different concentrations of A_4_, B_4_, and N_4_ Differences between groups in (A), (B), (E) and (F) were analyzed by one-way ANOVA followed by Tukey’s multiple comparisons tests. Values with different superscripts (a, b, c, and …g) indicate a significant difference (*p <* 0.05). Data are the mean ± SD; n = 3. (G) TEM negative staining images of *S. aureus* ATCC 29213 treated with N_4_. Scale bars, 500 nm and 1 μm (H) SEM images of *S. aureus* ATCC 29213 treated with N_4_ Scale bars, 1μm. (I)TEM images of *S. aureus* ATCC 29213 treated with N_4_. Scale bars, 500 nm (J) Live/dead fluorescence imaging of *S. aureus* ATCC 29213 after N_4_ treatment at different concentrations. Scale bars, 100 μm. (K) The agglutination of *S. aureus* ATCC 29213 in cuvette induced by different concentrations of N_4._ (L) Bacterial content of the *S. aureus* ATCC 29213 supernatant in cuvettes treated with different concentrations of N_4_. (M) Flow Cytometry imaging of *S. aureus* ATCC 29213 after N_4_ treatment. N_4_ was used for detection at a concentration of 16 μM in (G), (H), (I) and (M).

The effect of the nano-short peptides on the cytoplasmic membrane (CM) integrity of *E. coli* ATCC 25922 was assessed using β-galactosidase-mediated hydrolysis of o-Nitrophenyl β-D-galactopyranoside (ONPG)^19^. As shown in Figure S14, only N_4_ induced a significant increase in absorbance at 420 nm: approximately 2-fold at 8 μM and 5-fold at 16 μM within 1800 s, demonstrating its rapid ability to disrupt the CM of *E. coli* ATCC 25922. In contrast, B_4_ exhibited limited membrane-disrupting efficacy, likely due to its reduced capacity to penetrate the double-membrane structure characteristic of Gram-negative bacteria.


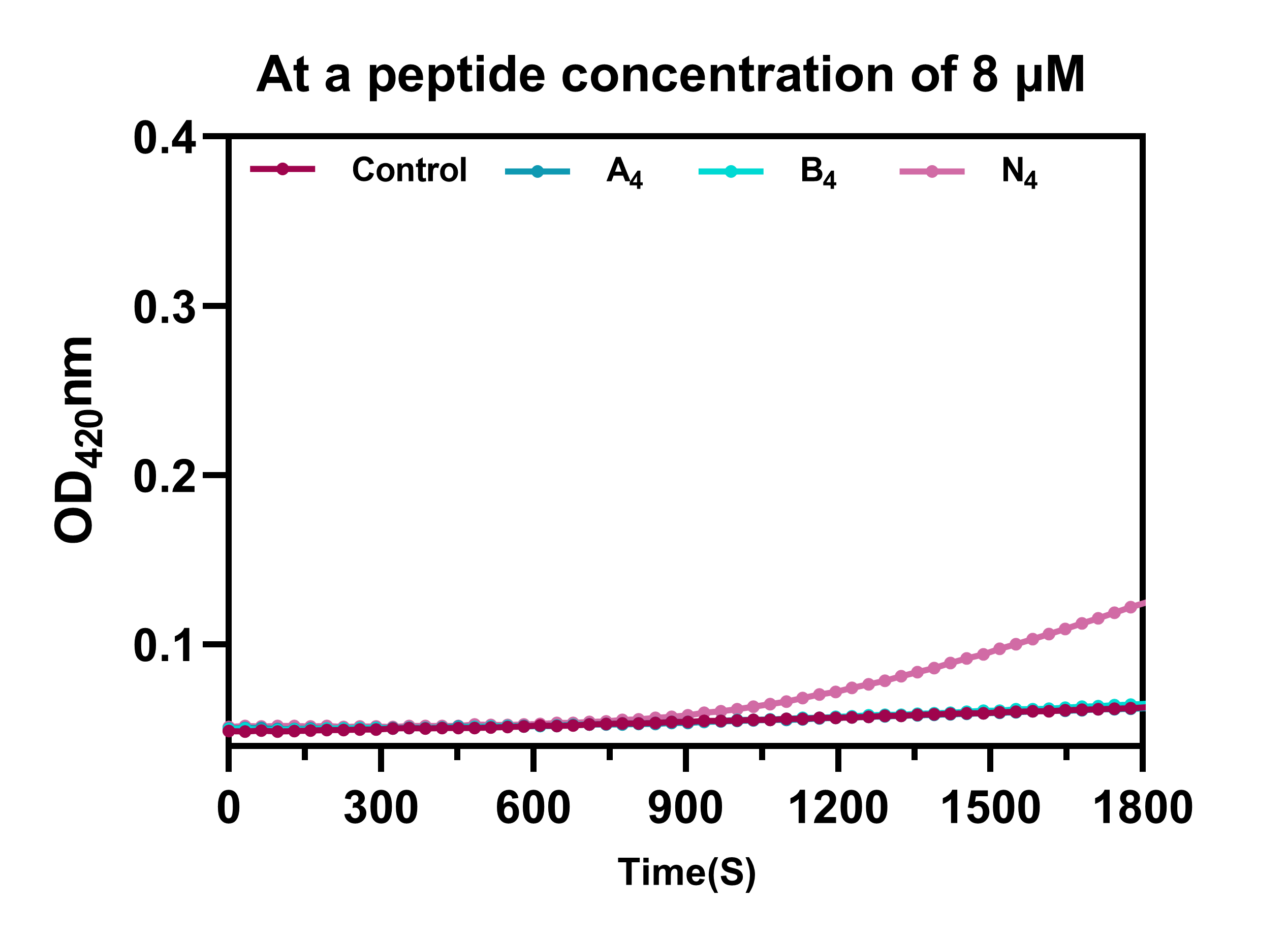

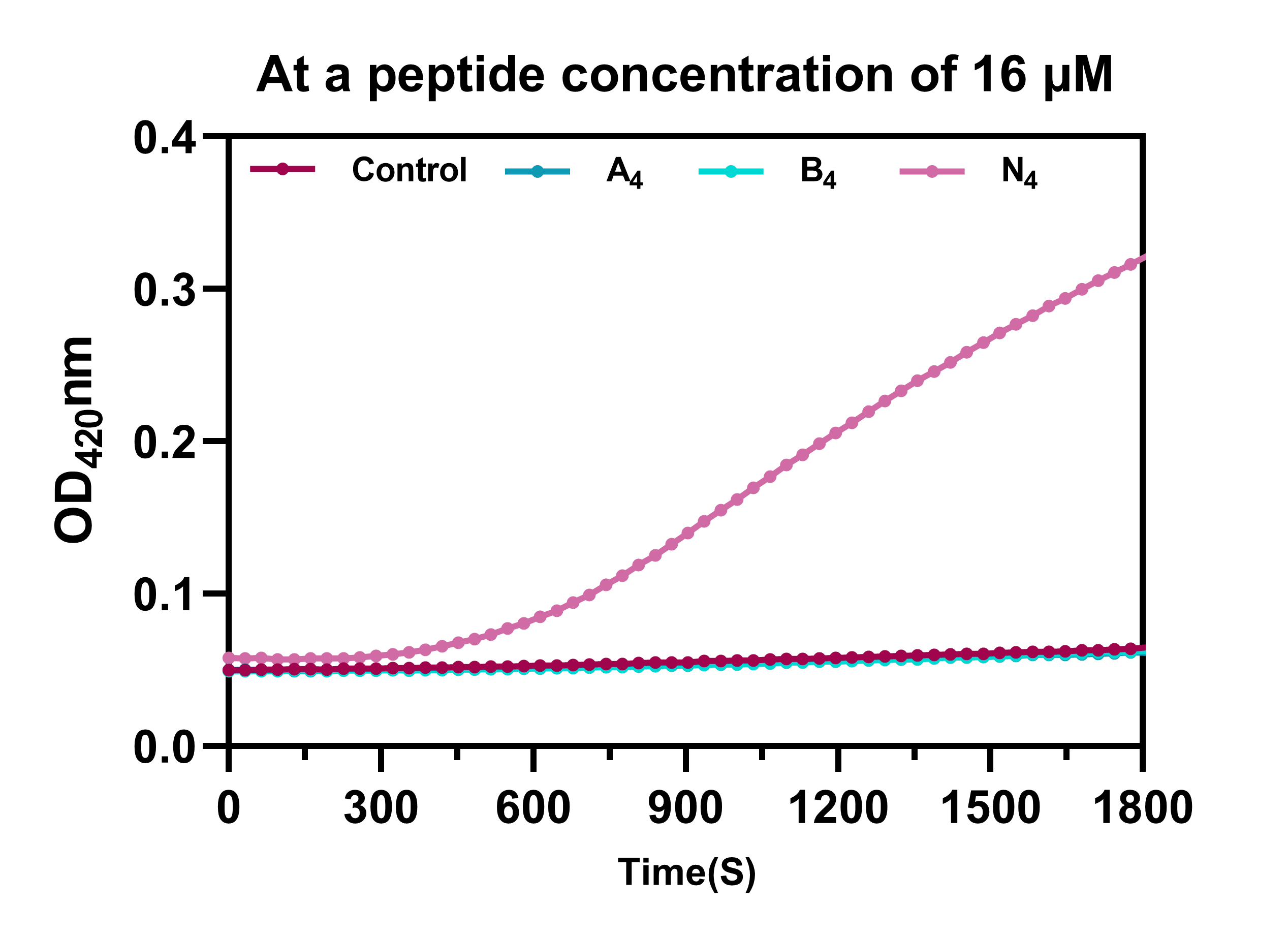


Figure S14. Effect of A_4_, B_4_, and N_4_ on the cytoplasmic membrane permeability of *E. coli* ATCC 25922.

ROS accumulation levels were assessed by the fluorescent probe DCFH-DA^20^.Compared to untreated bacterial samples, at a concentration of 16 μM, B_4_ and N_4_ increased the ROS levels in *E. coli* ATCC 25922 by 1.51-fold and 2.26-fold (Figure S15A), respectively, and in *S. aureus* ATCC 29213 by 1.38-fold and 1.83-fold, respectively (Figure S15B).


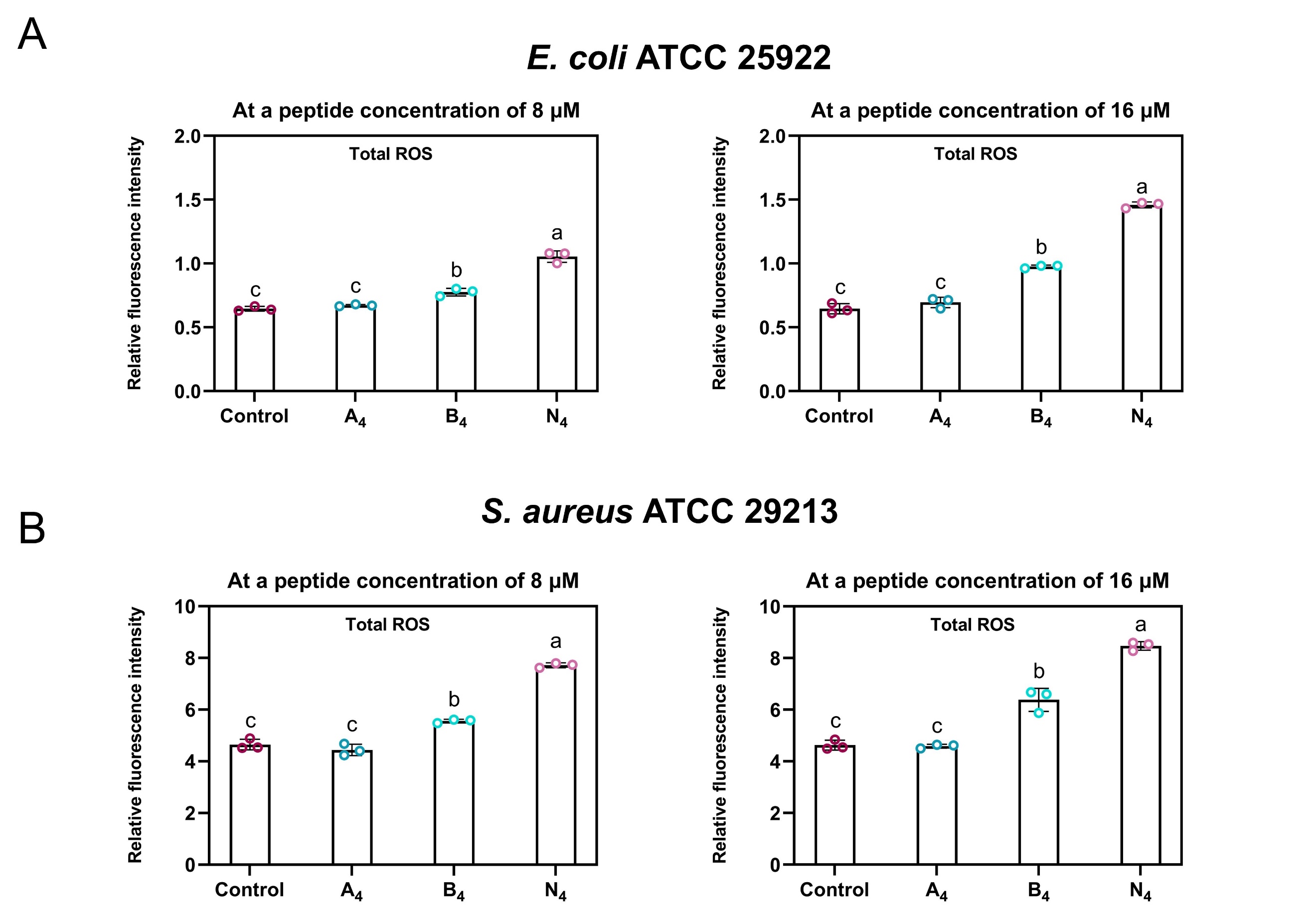


Figure S15. Effect of A_4_, B_4_, and N_4_ on ROS accumulation in *E. coli* ATCC 25922 and *S. aureus* ATCC 29213. Differences between groups were analyzed by one-way ANOVA followed by Tukey’s multiple comparisons tests. Values with different superscripts (a, b, and c) indicate a significant difference (*p* < 0.05). Data are the mean ± SD; n = 3

Figure S16 shows the retention profile of N_4_ after incubation with serum isolated from female ICR mice for varying durations. During the 12-hour monitoring period, N_4_ exhibited time-dependent progressive degradation in mouse serum, with over 50% remaining after the initial 4 hours. Notably, 8.1% of N_4_ persisted undegraded even after 12-hour serum exposure, demonstrating its remarkable stability in physiological conditions.


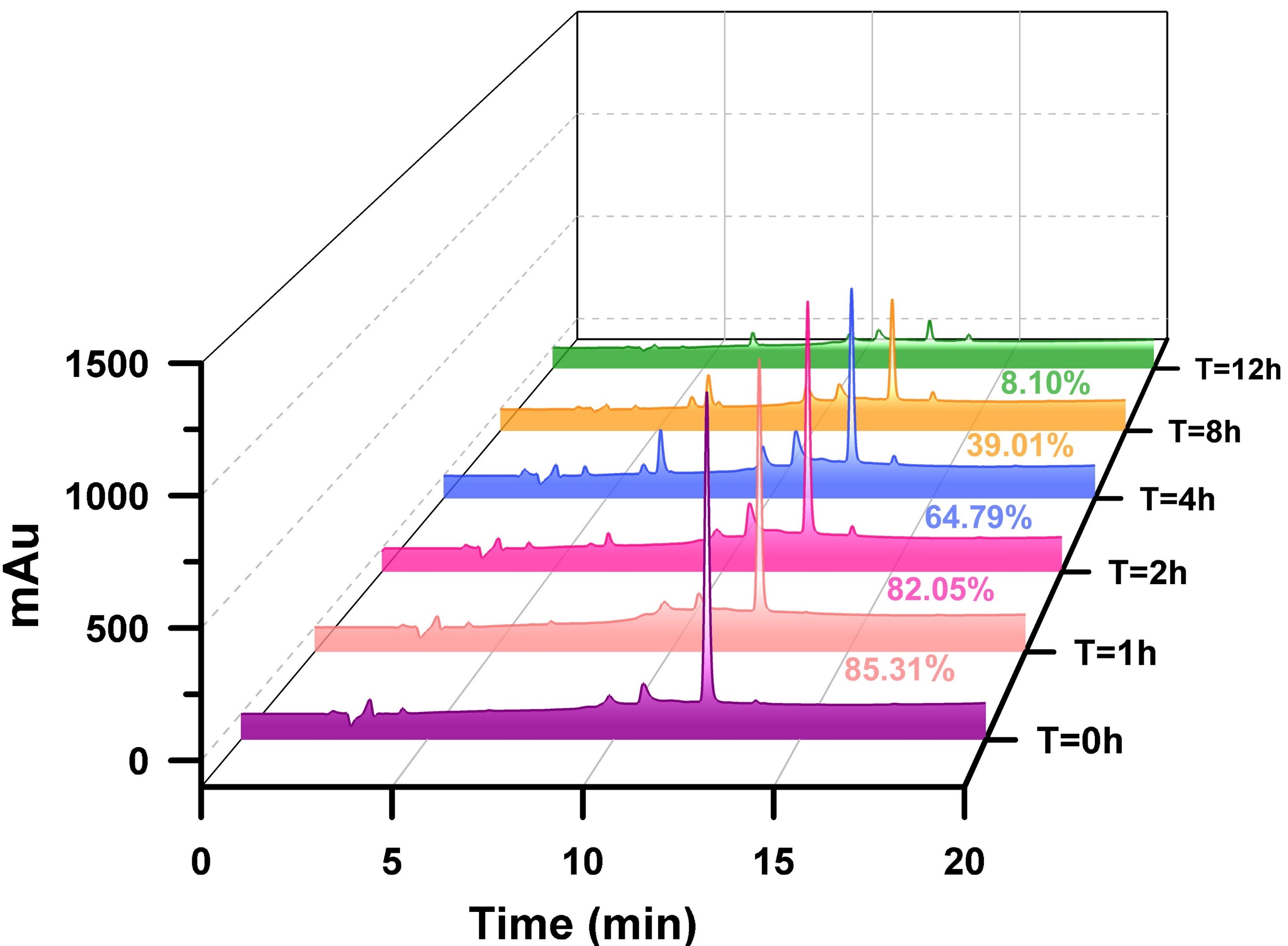


Figure S16. RP-HPLC analysis of N_4_ after incubation with serum isolated from mice for different periods of time.

Table S1 lists the sequences and physicochemical parameters of the nano-short peptides.

**Table S1** Sequence and key physicochemical parameters of nano-short peptides

| Peptide | Sequence | TMW*^a^*  g mol^-1^ | MMW*^b^*  g mol^-1^ | Purity  (%) | Charge number  (No.) |
| --- | --- | --- | --- | --- | --- |
| A_3_ | AAAR^D^RRP | 796.91 | 797.34 | 95.63 | 3 |
| A_4_ | AAAAR^D^RRP | 867.97 | 868.4 | 99.09 | 3 |
| A_5_ | AAAAAR^D^RRP | 939.05 | 939.5 | 96.16 | 3 |
| A_6_ | AAAAAAR^D^RRP | 1010.12 | 1011.6 | 98.77 | 3 |
| C_10_ | C_10_-R^D^RRP | 737.94 | 737.86 | 96.78 | 3 |
| C_12_ | C_12_-R^D^RRP | 766.01 | 766.48 | 98.72 | 3 |
| C_14_ | C_14_-R^D^RRP | 794.05 | 793.2 | 95.17 | 3 |
| C_16_ | C_16_-R^D^RRP | 822.10 | 822.5 | 95.56 | 3 |
| Ben | Ben-R^D^RRP | 687.80 | 688.26 | 97.26 | 3 |
| Nap | Nap-R^D^RRP | 737.86 | 736.79 | 98.54 | 3 |
| Ant | Ant-R^D^RRP | 787.92 | 788.32 | 96.62 | 3 |
| Pyr | Pyr-R^D^RRP | 811.94 | 812.26 | 98.50 | 3 |
| B_3_ | BuaBuaBua-R^D^RRP | 966.01 | 966.42 | 95.13 | 3 |
| B_4_ | BuaBuaBuaBua-R^D^RRP | 1093.44 | 1093.90 | 97.61 | 3 |
| B_5_ | BuaBuaBuaBuaBua-R^D^RRP | 1220.88 | 1221.3 | 95.00 | 3 |
| B_6_ | BuaBuaBuaBuaBuaBua-R^D^RRP | 1348.32 | 1348.74 | 98.28 | 3 |
| N_3_ | NalNalNal-R^D^RRP | 1176.18 | 1176.48 | 96.66 | 3 |
| N_4_ | NalNalNalNal-R^D^RRP | 1373.68 | 1374.12 | 97.77 | 3 |
| N_5_ | NalNalNalNalNal-R^D^RRP | 1571.18 | 1571.46 | 96.81 | 3 |
| N_6_ | NalNalNalNalNalNal-R^D^RRP | 1768.68 | 1768.98 | 97.93 | 3 |

^a^ Theoretical molecular weight. ^b^ Measured molecular weight.

As shown in Table S2, quantitative DFT calculations revealed binding energies of -5.96 kcal mol^-1^ for cation–π interactions, -2.77 kcal mol^-1^ for hydrogen bonding and -7.29 kcal mol1^-1^ for π–π stacking.

**Table S2** Calculation of the binding energy of the N_4_ intermolecular force

|  | Cation-π  kcal mol^-1^ | Hydrogen bonding  kcal mol^-1^ | π-π stacking  kcal mol^-1^ |
| --- | --- | --- | --- |
| Complex | -827.42 | -942.44 | -889.62 |
| Stru1 | -503.76 | -343.16 | -425.15 |
| Stru2 | -323.65 | -599.27 | -464.45 |
| deltaE in hartree | -0.01 | 0.00 | -0.01 |
| deltaE in kcal mol^-1^ | -5.96 | -2.77 | -7.29 |

Table S3 lists the detailed secondary structure composition of N_4_ within the amide I band at different wavelengths.

**Table S3** The amide I band in the FTIR spectrum of N_4_ analysis.

| Range (cm^-1^) | Attribution of the corresponding peaks | Proportion |
| --- | --- | --- |
| 1618-1640 | parallel β-sheet | 22.37% |
| 1640-1650 | random coil | 14.44% |
| 1650-1660 | α-helix | 16.38% |
| 1660-1670 | β-turn | 19.30% |
| 1670-1690 | antiparallel β-sheet | 27.51% |

References

(1) Zhu, Y.; Akhtar, M. U.; Li, B.; Chou, S.; Shao, C.; Li, J.; Shan, A. The design of cell-selective tryptophan and arginine-rich antimicrobial peptides by introducing hydrophilic uncharged residues. *Acta Biomaterialia* **2022**, *153*, 557-572.

(2) Friedrich, C.; Scott, M. G.; Karunaratne, N.; Yan, H.; Hancock, R. E. Salt-resistant alpha-helical cationic antimicrobial peptides. *Antimicrobial Agents and Chemotherapy* **1999**, *43* (7), 1542-1548.

(3) Huang, J.; Hao, D.; Chen, Y.; Xu, Y.; Tan, J.; Huang, Y.; Li, F.; Chen, Y. Inhibitory effects and mechanisms of physiological conditions on the activity of enantiomeric forms of an α-helical antibacterial peptide against bacteria. *Peptides* **2011**, *32* (7), 1488-1495.

(4) Ruankham, W.; Phopin, K.; Pingaew, R.; Prachayasittikul, S.; Prachayasittikul, V.; Tantimongcolwat, T. In silico and multi-spectroscopic analyses on the interaction of 5-amino-8-hydroxyquinoline and bovine serum albumin as a potential anticancer agent. *Sci Rep* **2021**, *11* (1), 20187.

(5) Hirose, S.; Hioki, Y.; Miyashita, H.; Hirade, N.; Yoshitake, J.; Shibata, T.; Kikuchi, R.; Matsushita, T.; Chikazawa, M.; Itakura, M.; et al. Apolipoprotein e binds to and reduces serum levels of DNA-mimicking, pyrrolated proteins. *The Journal of Biological Chemistry* **2019**, *294* (28), 11035-11045.

(6) van der Vusse, G. J. Albumin as fatty acid transporter. *Drug Metab Pharmacokinet* **2009**, *24* (4), 300-307.

(7) Lai, Z.; Yuan, X.; Chen, H.; Zhu, Y.; Dong, N.; Shan, A. Strategies employed in the design of antimicrobial peptides with enhanced proteolytic stability. *Biotechnol Adv* **2022**, *59*, 107962.

(8) Yu, W.; Guo, X.; Li, Q.; Li, X.; Wei, Y.; Shao, C.; Zhang, L.; Wang, J.; Shan, A. Revolutionizing antimicrobial biomaterials: Integrating an enzyme degradation-resistant sequence into self-assembled nanosystems to overcome stability limitations of peptide-based drugs. *Advanced Fiber Materials* **2024**.

(9) Yu, W.; Zhao, M.; Guo, X.; Wang, X.; Wang, J.; Lyu, Y.; Shan, A. Proteolytic-resistant self-assembling peptide nanofibers combat specific bacterial infections via trap and kill. *Sci Adv 11* (29), eadx0153.

(10) Li, G.; Deng, H.; Xu, W.; Chen, W.; Lai, Z.; Zhu, Y.; Zhang, L.; Shao, C.; Shan, A. Combating antibiotic-resistant bacterial infection using coassembled dimeric antimicrobial peptide-based nanofibers. *ACS Nano* **2025**, *19* (3), 3155-3171.

(11) He, J.; Wang, R.; Feng, W.; Chen, Z.; Wang, T. Design of novel edible hydrocolloids by structural interplays between wheat gluten proteins and soy protein isolates. *Food Hydrocolloids* **2020**, *100*, 105395.

(12) Wu, W.; Song, J.; Li, T.; Li, W.; Wang, J.; Wang, S.; Dong, N.; Shan, A. Unlocking antibacterial potential: Key-site-based regulation of antibacterial spectrum of peptides. *Journal of Medicinal Chemistry* **2024**, *67* (5), 4131-4149.

(13) Han, J.; Zhao, X.; Zhao, X.; Li, P.; Gu, Q. Insight into the structure, biosynthesis, isolation method and biological function of teichoic acid in different gram-positive microorganisms: A review. *International Journal of Biological Macromolecules* **2023**, *253* (Pt 5), 126825.

(14) Zhu, Y.; Shao, C.; Li, G.; Lai, Z.; Tan, P.; Jian, Q.; Cheng, B.; Shan, A. Rational avoidance of protease cleavage sites and symmetrical end-tagging significantly enhances the stability and therapeutic potential of antimicrobial peptides. *Journal of Medicinal Chemistry* **2020**, *63* (17), 9421-9435.

(15) Cabrini, G.; Verkman, A. S. Potential-sensitive response mechanism of dis-c3-(5) in biological membranes. *J Membr Biol* **1986**, *92* (2), 171-182.

(16) Yu, W.; Guo, X.; Li, X.; Wei, Y.; Lyu, Y.; Zhang, L.; Wang, J.; Shan, A. Novel multidomain peptide self-assembly biomaterials based on bola structure and terminal anchoring: Nanotechnology meets antimicrobial therapy. *Mater Today Bio* **2024**, *28*, 101183.

(17) Fan, Y.; Li, X.-D.; He, P.-P.; Hu, X.-X.; Zhang, K.; Fan, J.-Q.; Yang, P.-P.; Zheng, H.-Y.; Tian, W.; Chen, Z.-M.; et al. A biomimetic peptide recognizes and traps bacteria in vivo as human defensin-6. *Sci Adv* **2020**, *6* (19), eaaz4767.

(18) Li, Q.; Li, J.; Yu, W.; Wang, Z.; Li, J.; Feng, X.; Wang, J.; Shan, A. De novo design of a ph-triggered self-assembled β-hairpin nanopeptide with the dual biological functions for antibacterial and entrapment. *Journal of Nanobiotechnology* **2021**, *19* (1), 183.

(19) He, S.; Yang, Z.; Li, X.; Wu, H.; Zhang, L.; Shan, A.; Wang, J. Boosting stability and therapeutic potential of proteolysis-resistant antimicrobial peptides by end-tagging β-naphthylalanine. *Acta Biomaterialia* **2023**, *164*, 175-194.

(20) He, S.; Yang, Z.; Li, X.; Wu, H.; Zhang, L.; Wang, J.; Shan, A. Optimized proteolytic resistance motif (dabw)-based u1-2wd: A membrane-induced self-aggregating peptide to trigger bacterial agglutination and death. *Acta Biomaterialia* **2022**, *153*, 540-556.
